# Bacterial communities of wild bee species and the western honey bee (*Apis mellifera*) (Hymenoptera: Apoidea): Alpine insights

**DOI:** 10.1093/jisesa/ieaf095

**Published:** 2025-11-07

**Authors:** Fabian P Royer, Julia S Schlick-Steiner, Thomas Klammsteiner, Timotheus Kopf, Birgit C Schlick-Steiner, Florian M Steiner

**Affiliations:** Department of Ecology, Faculty of Biology, University of Innsbruck, Innsbruck, Austria; Department of Ecology, Faculty of Biology, University of Innsbruck, Innsbruck, Austria; Department of Ecology, Faculty of Biology, University of Innsbruck, Innsbruck, Austria; Department of Ecology, Faculty of Biology, University of Innsbruck, Innsbruck, Austria; Department of Ecology, Faculty of Biology, University of Innsbruck, Innsbruck, Austria; Department of Ecology, Faculty of Biology, University of Innsbruck, Innsbruck, Austria

**Keywords:** Apidae, insect pollinator decline, intestinal microbiome, spillover, 16S rDNA metabarcoding

## Abstract

Wild bees are decreasing in species diversity and populations due to human impact. The abundance of the western honey bee (*Apis mellifera* L.) experiences an inverse trend, enhancing competition with wild bees and the probability of microbiome exchange. Addressing this exchange, we studied the gut microbiome composition of wild and honey bees, focusing on patterns indicating honey bee influence. Three solitary wild bee species (large scabious mining bee [*Andrena hattorfiana* F.], grey-backed mining bee (*Andrena vaga* Panzer), and European orchard bee [*Osmia cornuta* Latreille]) as well as bumble bees as representatives of eusocial wild bees (*Bombus* spp. Latreille) and honey bees were sampled in the Austrian Alps. Subsequent 16S ribosomal DNA sequencing revealed the composition of the bacterial communities. The bee groups differed concerning their bacterial composition, with honey bees having the least variation among individuals and a low number of exclusive bacterial taxa and bumble bees the highest bacterial diversity. High honey bee densities corresponded with lower bacterial diversity in wild bees and a higher bacterial similarity between wild and honey bees. Some bacterial taxa were found for the first time in the studied bee groups. Furthermore, the composition of bacterial communities differed between solitary and social bees. We found the first hints that high honey bee density negatively impacts wild bees through alterations of wild bee microbiomes. Future studies should focus on understanding microbiome transmission mechanisms and their consequences for wild bees. Suggestions on how to consider wild bee fitness are indispensable in halting the biodiversity crisis.

## Graphical abstract

**Graphical Abstract:**
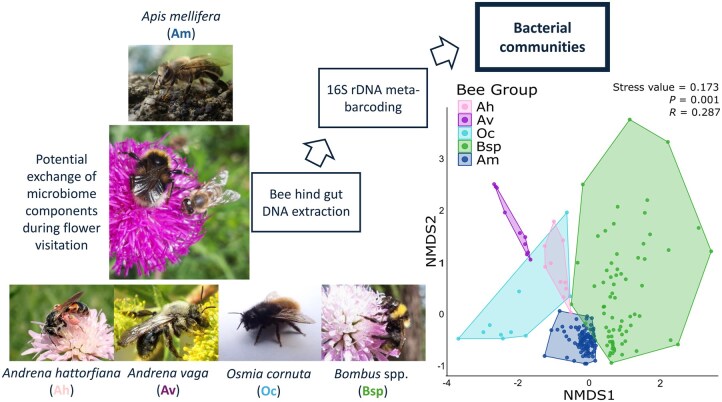

## Introduction

Wild bees and the western honey bee (*Apis mellifera* L., Hymenoptera: Apidae) play a key role in natural and agricultural ecosystems, as they are responsible for pollination of many wild plants and crops (Khalifa et al. 2021). In Austria, 702 species of wild bees are recorded (Wiesbauer 2020), nearly half of them being endangered (Westrich 2019, Müller and Praz 2024) and facing decreases in population sizes, mostly because of anthropogenically induced environmental changes, above all land use change and pesticides (Dicks et al. 2021). In parallel to the decline of wild pollinators, the density of the managed honey bee is increasing as beekeeping is becoming ever more popular; first findings indicate competition for the floral resources nectar and pollen between wild bees and honey bees (Mallinger et al. 2017, Wojcik et al. 2018, Osterman et al. 2021, Iwasaki and Hogendoorn 2022 [including studies on Central Europe and Austria], FAOSTAT, http://faostat.fao.org [Austria]). Furthermore, high honey bee density may increase the probability of pathogen infections and generally the exchange of microbes between honey and wild bees during flower visitation (Fürst et al. 2014, Zhang and Zheng 2022). This exchange could have adverse effects for the bees through alterations of their microbiome, that is, all bacteria, fungi, viruses, and protists in and on an organism (Nguyen and Rehan 2023).

The relevance of the microbiome for the health and fitness of bees is increasingly being unveiled (Voulgari-Kokota et al. 2019). At least in honey bees, it is fundamental for digestion, immune response, defense from pathogens (Zhang and Zheng 2022), and adaptation to environmental conditions; in wild bees, these mechanisms are still rather unclear (Hammer et al. 2021). Regarding the adaptation to environmental conditions, rapid human interventions in the landscape and land use impact the microbiome and thus the fitness of bees (Nguyen and Rehan 2023). A better understanding of the structure and plasticity of microbial communities in wild bees is therefore crucial for assessing the risk potential of these anthropogenic environmental changes and implementing effective conservation measures. This plasticity is enabled by various transmission mechanisms, dependent on the ecology and sociality of bee species (Voulgari-Kokota et al. 2019). Besides eusocial bees such as honey bees and bumble bees, the majority of wild bees are solitary (Westrich 2019). In eusocial bees, transmission occurs mostly through social activity, like nurse bees feeding larvae and interaction of adults (Lignon et al. 2024). Because of these constant interactions within the nests of eusocial bees, a core microbiome is established in each nest (Nguyen and Rehan 2023). In solitary bees, the mother transfers microbes indirectly through saliva to pollen provisions and nesting material in the brood cell and thus to the offspring (Lignon et al. 2024). After leaving the nest, the environment is the main source for their microbiota (Nguyen and Rehan 2023). Foraging exposes both eusocial and solitary bees to various microbes, as flowers act as a hub for multidirectional transmissions among flowers and various pollinators (Lignon et al. 2024). Due to a complex causal network of varying interactions and environmental conditions, the microbial community structure seems to be diverse and fluctuating, particularly in wild bees (Voulgari-Kokota et al. 2019). Furthermore, for wild bees, knowledge regarding the role of the microbiome and the exchange mechanisms is only based on a few species and thus quite sparse.

Bacteria constitute the most dominant part of the microbiome (bacteriobiota) (Nguyen and Rehan 2023) and are therefore the focus of this study. Moreover, they are more readily accessed than fungi and viruses because of greater ease in molecular identification, allowing a broad characterization of the bacterial structure in bees within the study area. We aimed to characterize (i) the diversity of the bacterial community of wild bees and honey bees, (ii) patterns of their exclusive and shared bacteria, and (iii) a potential relationship between honey bee density and bacterial diversity of wild bees and honey bees. To address these questions, we sampled wild bees and honey bees (*A. mellifera*) in and around the city of Innsbruck in the European Alps. The wild bees sampled comprised 3 solitary species: large scabious mining bee (*Andrena hattorfiana* F., Hymenoptera: Andrenidae), grey-backed mining bee (*Andrena vaga* Panzer, Hymenoptera: Andrenidae), and European orchard bee (*Osmia cornuta* Latreille, Hymenoptera: Megachilidae), as well as bumble bees as representatives of eusocial wild bees (*Bombus* spp. Latreille, Hymenoptera: Apidae). Wild bee and honey bee individuals were sampled simultaneously at various sampling sites, including altitudinal transects. This allowed for a range of honey bee densities and, through these, for a range of probabilities of contact between wild bees and honey bees.

## Materials and Methods

### Bee Species Studied

The study organisms were honey bees, *A. mellifera* (Am), and the wild bees large scabious mining bee *A. hattorfiana* (Ah), grey-backed mining bee *A. vaga* (Av), European orchard bee *O. cornuta* (Oc), and non-parasitic *Bombus* spp. (Bsp). Ah and Av nest in the ground (Westrich 2019) and are oligolectic bee species visiting nearly exclusively field scabious (*Knautia arvensis* L.) and small scabious (*Scabiosa columbaria* L.) (Ah) and willows (*Salix* spp. L.) (Av), respectively (Westrich 2019). Oc nests in various cavities, like plant stalks and old perforated walls; it is synanthropic, preferring the mild microclimate of settlements (Westrich 2019). It is polylectic just like Am and Bsp (Supplementary Table S1). Bsp is represented by 45 species in Austria (Gokcezade et al. 2023). Bumble bees mostly nest in the ground, for example, in already existing cavities like mouse holes; nest sizes differ, depending on species and time of the season, between 30 and 600 individuals (Amiet and Krebs 2019).

### Sites and Sampling Concept

This study was conducted in and around Innsbruck, a city of ca. 130,000 inhabitants in Austria, in the Eastern European Alps. It is located in the west-east-oriented Inn Valley, formed by the river Inn, with a temperate climate. In the north, it is bordered by the Karwendel mountain range (limestone Alps, up to 2,749 m above sea level [m a.s.l.]) with the Nordkette as the nearest mountain chain; to the south, the Tux Alps border (gneiss rock, up to 2,886 m a.s.l.); a side valley of the Inn Valley is the north-south oriented, inner alpine Ötz Valley (gneiss rock, up to 3,768 m a.s.l.); its entrance lies 40 km west of Innsbruck (Kompass Wanderkarte 2025).

The sampling year 2022 was characterized by a new mean temperature record for Tyrol (4.5 °C [+2.4 °C compared with long-term mean]) and an exceptionally dry and sunny spring and summer; especially March was very dry (10 mm [−80 mm]) and sunny (198 h [+70 h]); exceptions of this trend between January and August are only high precipitation rates in February (88 mm [+21 mm]) and June (196 mm [+34 mm]); there were some (∼6 to 9) days in the beginning of March and April with temperatures below average (Hiebl and Orlik 2023).

**Fig. 1.**
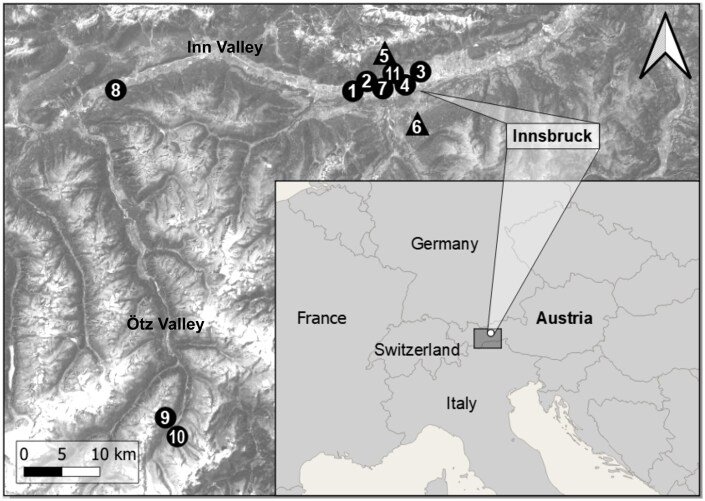
Study area in Tyrol (Austria) with sampling sites 1 to 11. *Andrena hattorfiana* was sampled at sites 7 and 8 (5 individuals per site); *Andrena vaga* at 1 and 2 (5); *Osmia cornuta* at 3 and 4 (5); *Bombus* spp. at 5 (31), 6 (30), 9 and 10 (5); *Apis mellifera* at all sites: 1 to 4 (5), 5 and 6 (25), 7 to 10 (5), 11 (10). Sites 5 and 6 were altitudinal transects. For more information, see Supplementary Table S1. Made with QGIS (Main map based on Esri (2025): Sources: Esri, DigitalGlobe, GeoEye, i-cubed, USDA FSA, USGS, AEX, Getmapping, Aerogrid, IGN, IGP, swisstopo, and the GIS User Community. Smaller overview map: Made with Natural Earth. Free vector and raster map data @ naturalearthdata.com.).

From April 11 to 10 August 2022, bees were sampled at 11 sites, 9 of them in the Inn Valley and 2 in the Ötz Valley (Fig. 1). The sites differed in the intensity and type of land use as well as in ecosystem covers; additionally, sites 5, 6 (altitudinal transects), 9, and 10 (including different elevations) showed differences within the same sites (Supplementary Material). For every wild bee individual, a corresponding honey bee individual was sampled in close spatial and temporal proximity. Workers of Am were sampled at all sites, females of Ah, Av, and Oc each at 2 sites, and workers of Bsp at 4 sites (Fig. 1, Supplementary Table S1).

### Sampling of Bumble Bees on Genus Level

Bumble bees were sampled at the genus level to ensure covering the elevational transects. Determination on the species level was not possible for all individuals directly in the field. Furthermore, several elevations, especially in forest-dominated areas, harbored very few bumble bees, which made targeted selection of a certain species impossible. After sampling, the bacterial communities of all individuals of the 12 bumble bee species collected were analyzed together to achieve the sample size needed.

### Sampling Procedure

Bees were caught using an insect net. In the case of bumble bees, the body length was measured, and only individuals smaller than 15 mm were sampled to avoid sampling the larger queens or females of parasitic bumble bee species (Wiesbauer 2020). The bees were then transferred to sterile 50 ml vials and anesthetized with carbon dioxide. Using a sterile plastic forceps, the bees were put into smaller tubes containing the conservation liquid RNAprotect (Qiagen, Hilden, Germany), keeping out air bubbles with sterile gauze balls. The tubes were immediately chilled in a cold box. For every bee individual sampled, GPS coordinates, elevation, and, as a measure for honey bee density, the number of Am individuals observed simultaneously (n-*A. mellifera*-simultaneous) were recorded (Supplementary Table S1).

In the laboratory, bumble bees were measured more precisely, and using bee species length information (Wiesbauer 2020), it was double-checked that all individuals sampled were workers. Bumble bee individuals were determined at the species level (Amiet et al. 2017, Gokcezade et al. 2023). For optimal conservation, all bees were punctured using a sterile insect needle. The head was punctured once or twice and the thorax and abdomen at least 3 times each, according to the body size of the respective bee individual. The punctured bees were cooled at 4 °C for 48 h and then stored at −20 °C until DNA extraction.

### DNA Extraction

Bees were handled as cold (on ice, shortest possible time outside the freezer) and as sterile (sterile pulp as underlay, heat-sterilized forceps, under UV-C hood [UVC/T-AR, BioSan, Riga, Latvia], sterile single-use scalpels) as possible. Prior to DNA extraction, bees were washed with sterile MilliQ water and observed for possible symptoms of diseases like malformation (all bees lacked visible symptoms). Abdomina were homogenized in a TissueLyser (Qiagen, Hilden, Germany) at 30 Hz for 3 min with 1 metal bead (diameter 5 mm, Qiagen, Hilden, Germany) and a pinch of glass beads (diameter 0.5 mm, Sigma-Aldrich, St. Louis, MO, United States) each. Additionally, 20 µl of Proteinase-K were added (incubation 10 min, 56 °C, 400 rpm). Because of the higher amount of tissue in bumble bee abdomina, 375 µl of buffer RLT was added additionally. Apart from this, DNA extraction and purification followed the protocol of the AllPrep DNA/RNA Mini Kit (Qiagen, Hilden, Germany). DNA concentrations were measured with Implen NanoPhotometer N60 UV/Vis-Spectrophotometer (Fisher Scientific, Schwerte, Germany), and no-template controls were checked with Invitrogen Qbit 3.0 Fluorometer (Fisher Scientific, Schwerte, Germany).

### 16S Ribosomal DNA Metabarcoding

DNA extracts in sufficient quantity and quality were sent to a commercial provider (Novogene, Cambridge, UK) for targeted 16S rRNA gene sequencing. The V4 region was amplified using the universal primer pair 515F (5′-GTGCCAGCMGCCGCGGTAA-3′) and 806R (5′-GGACTACHVGGGTWTCTAAT-3′) (Caporaso et al. 2011), using a 2×300 base pairs approach. Sequencing was performed on the Illumina NovaSeq 6000 platform (Illumina, San Diego, CA, United States). Briefly, paired-end reads were assigned to samples using their unique barcodes with subsequent cutting of barcodes and primer sequences (Python v3.6.13, cutadapt v3.3 [Martin 2011]) and were merged, obtaining raw tags (FLASH v1.2.11 [Magoc and Salzberg 2011]). The raw tags were quality filtered (fastp v0.23.1 [Bokulich et al. 2013]), and chimaeras were removed by comparison with a reference database (Silva v. 138 Database, https://www.arb-silva.de/ [Quast et al. 2012]; UCHIME [Edgar et al. 2011]). The obtained effective tags (vsearch v2.16.0 [Edgar et al. 2011]) were clustered and assigned to Operational Taxonomic Units (OTUs) applying a  ≥97% similarity threshold (Uparse v7.0.1001 [Edgar 2013]). These OTUs were taxonomically assigned using the Silva Database (Quast et al. 2012) and the Mothur algorithm (Schloss et al. 2009). Besides this, Novogene used R version 4.0.3 (R Core Team 2024) and QIIME version 1.9.1 (Caporaso et al. 2010).

### Statistics

All statistical analyses were performed in R version 4.3.3 (R Core Team 2024). A phyloseq (McMurdie and Holmes 2013) object was created, and data were cleaned by removing Archaea, mitochondria, and chloroplasts. Rare OTUs were filtered by keeping only OTUs that existed in at least 1 sample twice or more often and in least in 2.5% of all samples. These filtered data were normalized using the method of scaling with ranked subsampling and the package SRS (Beule and Karlovsky 2020). The most abundant bacterial phyla were identified and visualized in a bar plot with ggplot2 (Wickham 2016), phyla of relative abundance less than 0.5% were excluded, and the number of OTUs (alpha diversity index “observed”) was computed. With the package microbiome (Lahti and Shetty 2019), alpha diversity indices were calculated, and a boxplot with the index “observed” (number of observed OTUs) was produced with ggplot2 (Wickham 2016). The phyloseq object was transformed into an ampvis object. Beta diversity of the bacterial communities among study species was assessed by non-metric multidimensional scaling (NMDS) with Bray–Curtis distance measure, computed with ampvis2 (Andersen et al. 2018), and Linear discriminant analysis Effect Size (LEfSe), computed with microbiomeMarker (Cao et al. 2022). Analysis of Similarities (ANOSIM) was done with vegan (Oksanen et al. 2001). Furthermore, a heatmap and a Sankey plot showing the 15 most abundant bacterial genera were produced using ampvis2 (Andersen et al. 2018) and ggplot2 (Wickham 2016) with ggalluvial (Brunson and Read 2023), respectively. With the package MicEco (Russel 2021), Venn diagrams were generated both with weighted OTU abundances and with unweighted OTU abundances. Furthermore, the effect of n-*A. mellifera*-simultaneous on alpha diversity (“observed”) and dissimilarity (differences in bacterial diversity between corresponding honey bee and wild bee), respectively, was tested for significance with linear regressions (alpha = 0.05). The values of n-*A. mellifera*-simultaneous were log-transformed. Bray–Curtis dissimilarities were computed for corresponding bee pairs using vegan (Oksanen et al. 2001). Samples from sites 9 to 11 were excluded because no corresponding bee pairs could be formed given that the honey bees sampled were not caught in spatial nor temporal proximity to the wild bees (sites 9 and 10) or only honey bees were sampled (site 11).

## Results

In total, 201 bee individuals were caught, that is, 100 honey bees (Am) and 101 wild bees. Except for the altitudinal transects at sites 5 and 6, 5 honey bee and 5 wild bee individuals were sampled per site. There were 10 individuals each of Ah, Av, and Oc. The total number of Bsp individuals was 71, representing 12 species (Fig. 1 caption, Supplementary Fig. S1). The 16S metabarcoding produced sufficient data for all samples, resulting in 12,652 reads per bee and 997 OTUs after filtering and cleaning (Supplementary Table S2).

**Fig. 2.**
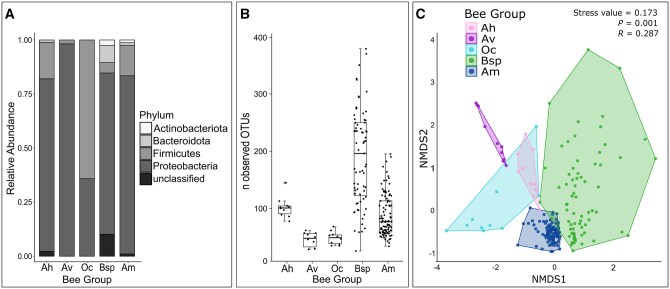
Bacterial diversity of bee groups *Andrena hattorfiana* (Ah, *n* = 10), *Andrena vaga* (Av, *n* = 10), *Osmia cornuta* (Oc, *n* = 10), *Bombus* spp. (Bsp, *n* = 71), and *Apis mellifera* (Am, *n* = 100). A). Relative abundance of most common bacterial phyla with abundance >0.5%. B). Alpha diversity, expressed as number of observed bacterial Operational Taxonomic Units (OTUs); boxes represent second and third quartiles; whiskers are based on 1.5 ×interquartile ranges; bold lines represent median; dots represent bee individuals. C). Non-metric multidimensional scaling (NMDS), distance measure = Bray–Curtis; *P*- and *R*-value obtained by Analysis of Similarities; dots represent bee individuals.

At the bacterial phylum level, the diversity and structure of the bacterial communities clearly differed across the 5 bee groups (Fig. 2A). Proteobacteria were the most abundant phylum for all bee groups except for Oc, in which Firmicutes dominated; the latter was present also in all other bee groups, albeit with much lower abundance. The group Bsp had the most diverse composition of bacterial phyla, in that the number of phyla was the same as in Am, but the evenness of OTUs was higher (Pielou’s evenness J: J_Am_ = 0.20, J_Bsp_ = 0.31).

Alpha diversity, expressed as the number of OTUs observed, differed among the bee groups (Fig. 2B, Kruskal–Wallis rank sum test: χ^*2*^ = 98.41, *df *= 4, *P *< 0.001). The median values ranged from 48 OTUs in Av to 196 OTUs in Bsp. Among Bsp samples, the number of OTUs observed differed widely; in contrast, Am samples clustered more densely, although they formed the largest group with 100 bee individuals, compared with 10 Ah, 10 Av, 10 Oc, and 71 Bsp individuals, respectively (Fig. 2C).

The large variety among Bsp samples (Fig. 2B and C) was not only interspecific but also intraspecific for some species. There was an even higher variety of OTUs within some Bsp species than among Am samples (Supplementary Figs S1 and S2).

Beta diversity analysis revealed a slight separation of the bee groups (Fig. 2C), which was confirmed by ANOSIM (*R *= 0.287, *P *= 0.001). The 100 individuals of Am, which accounted for half of all samples, clustered very densely. Moreover, Oc had a larger variation than Ah and Av, although it consisted of 10 individuals as well.

**Fig. 3.**
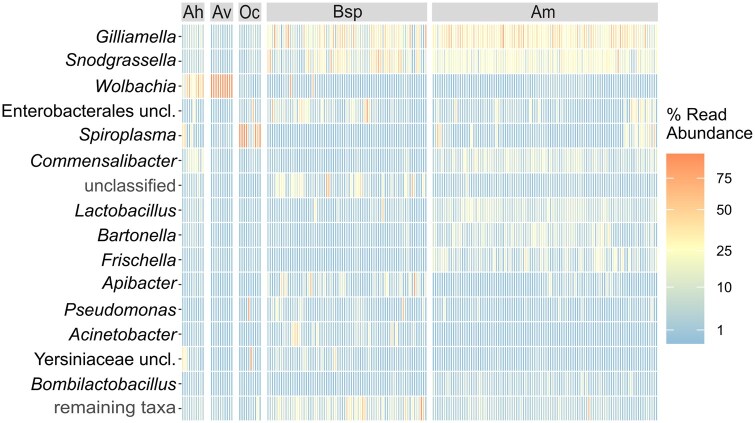
Distribution of the 15 most abundant bacterial taxa and the summed up abundance of all remaining taxa (last row) across bees. Abundances are the relative abundances of the bacterial taxa reads (“read abundance”). Bee samples (*n* = 201), represented by columns, are arranged in 5 groups: *Andrena hattorfiana* (Ah, *n* = 10), *Andrena vaga* (Av, *n* = 10), *Osmia cornuta* (Oc, *n* = 10), *Bombus* spp. (Bsp, *n* = 71), and *Apis mellifera* (Am, *n* = 100).

**Fig. 4.**
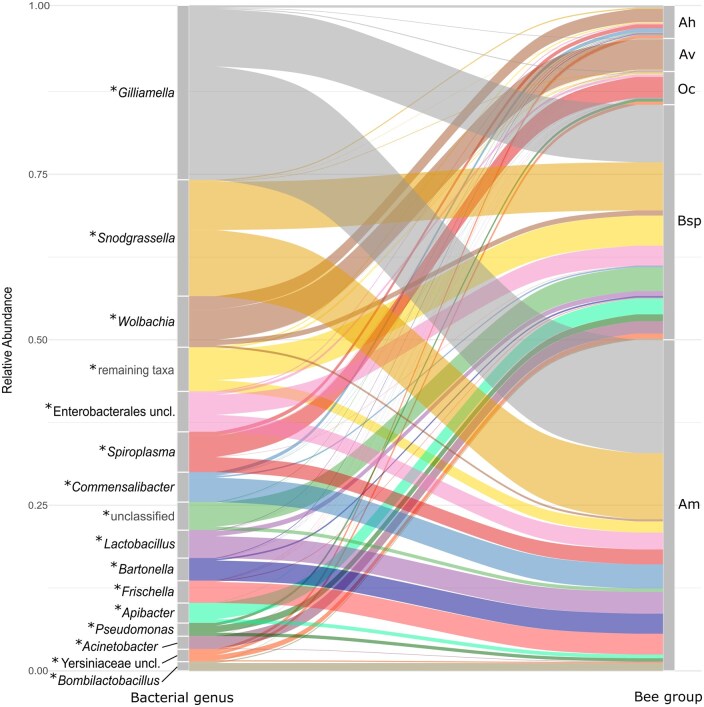
Distribution of 15 most abundant bacterial genera (left) over bee groups (right). Bee samples (*n* = 201), represented by columns, are arranged in 5 groups: *Andrena hattorfiana* (Ah, *n* = 10), *Andrena vaga* (Av, *n* = 10), *Osmia cornuta* (Oc, *n* = 10), *Bombus* spp. (Bsp, *n* = 71), and *Apis mellifera* (Am, *n* = 100). The * next to genus names indicates Kruskal–Wallis *P*-values < 0.005.

Considering the bacterial genera dominant in the bee groups, Ah had high proportions of *Wolbachia*, *Spiroplasma*, and *Commensalibacter*; Av a very high proportion of *Wolbachia*, and most samples of Oc had a very high proportion of *Spiroplasma*. Bsp and Am both had high proportions of *Gilliamella* and *Snodgrassella*, but differed concerning the other taxa; a genus in which a lot of differentiation occurred in Bsp was *Apibacter* (Figs 3 and 4).

The sequencing data also included OTUs of unclassified bacteria that could not be assigned to a phylum or, in the case of Enterobacterales and Yersiniaceae, to a family or genus, respectively. Besides the 15 most abundant taxa, all 354 remaining ones were combined in 1 group. These and most of the taxa mentioned above, which could not be assigned to a bacterial genus, had the highest abundances in Bsp individuals (Figs 3 and 4).

Twelve of the 15 most abundant bacterial taxa were also the ones that best differentiated the bee groups (calculated with LEfSe, Supplementary Fig. S3). Further important genera that were characteristic of one of the 5 bee groups were *Asaia* in Ah, *Alkanindiges* and *Pedobacter* in Bsp, and *Stenotrophomonas* in Oc. All these genera had logarithmic discriminant analysis scores greater than 3, meaning that these genera differed across bee groups.

Analysis of shared and exclusive OTUs (Venn diagrams) revealed 662 (of a total of 997) OTUs shared between Am and wild bee samples (Ah, Av, Oc, Bsp), 28 OTUs exclusive to Am, and 347 OTUs exclusive to wild bees. Considering the relative abundance of the OTUs, the shared OTUs accounted for 98.6%, the exclusive OTUs of Am accounted for < 0.1%, and the exclusive OTUs of wild bees accounted for 1.4%, indicating very low abundances of exclusive OTUs. Excluding Ah, Av, and Oc resulted in a similar relation (97.9% shared, 0.1% Am, 2.0% wild bees), while excluding Bsp resulted in fewer differences between Am and wild bees (Ah, Av, Oc; 99.8% shared, 0.1% Am, < 0.1% wild bees).

Linear regressions indicated a significant negative relationship between the number of *A. mellifera* individuals observed simultaneously with the respective bee sample (n-*A. mellifera*-simultaneous) and elevation (*n *= 201, *P *< 0.001, *R^2^* = 0.27, *y *= −0.01*x* + 19.19), but no significant correlation when only testing Bsp or altitudinal transects. There was also a significant relationship between the (log-transformed) n-*A. mellifera*-simultaneous and the alpha diversity expressed as the number of OTUs observed per bee sample. This relationship was negative and consistent when testing for all bee samples (*n *= 201, *P *< 0.001, *R^2^* = 0.16, *y *= −2.08*x* + 133.79), exclusively Am samples (*n *= 100, *P *< 0.001, *R^2^* = 0.25, *y *= −0.99*x* + 96.43), and exclusively wild bees (Ah, Av, Oc, Bsp, *n *= 101, *P *< 0.001, *R^2^* = 0.16, *y *= −3.67*x* + 168.38). However, there was no clear correlation between elevation and alpha diversity. Although there were significant correlations for all bees (*n *= 201, *P *< 0.001, *R^2^* = 0.14, *y *= 0.05*x* + 60.07) and for Bsp at site 5 (*n *= 31, *P *= 0.009, *R^2^* = 0.19, *y *= 0.09*x* + 86.34), other subgroups were not significant. Altitudinal transects merely showed a slight increase of Shannon diversity with elevation (*n *= 111, *P *= 0.008, *R*  ^2^ = 0.05, *y *= 0.0004*x* + 1.66) but no significant increase in OTU numbers. Furthermore, there was a significant negative relationship between n-*A. mellifera*-simultaneous and the Bray–Curtis dissimilarity of the corresponding Am and wild bee samples (both bees sampled as close to each other as possible) regarding the bacterial diversity. The more *A. mellifera* individuals there were, the more similar was the bacterial diversity of the bees (*n *= 201, *P *= 0.001, *R^2^* = 0.12, *y *= −0.05*x* + 0.57).

## Discussion

Using a Central-European Alpine study system, we aimed to characterize (i) the diversity of the bacteriobiota of selected wild bees (*A. hattorfiana*, *A. vaga*, *O. cornuta*, *Bombus* spp.) and the honey bee (*Apis mellifera*), (ii) patterns of their exclusive and shared bacteria, and (iii) a potential relationship between honey bee density and bacterial diversity of wild bees and honey bees. Using 16S rDNA metabarcoding, we explored the bacterial composition of wild bees and simultaneously caught honey bee individuals.

Pursuing Aim 1, to characterize the bacteriobiota of wild bees and the honey bee, we found the bacterial community of Bsp to be the most diverse, followed by that of Am. In contrast, the non-social bee groups Ah, Av, and Oc harbored fewer bacterial taxa. The dominant bacteria phyla within the bee groups (relative abundance) are common gut microbiota (Voulgari-Kokota et al. 2019, Hammer et al. 2021, Nguyen and Rehan 2023).

The dominant genera in Bsp and Am, *Gilliamella* and *Snodgrassella*, are a characteristic part of the core microbiome of social bees (Hammer et al. 2021). Both mainly colonize the ileum and interact with each other by cross-feeding (Yang et al. 2024). *Gilliamella* degrades pectin, which is the major component of pollen cell walls (Kwong and Moran 2016). Furthermore, it has the potential to offset mannose toxification and thus is part of the immune response (Yang et al. 2024). In turn, *Snodgrassella* utilizes fermentation products of *Gilliamella* (Kwong and Moran 2016) and plays a major role in the immune system by increasing antimicrobial peptide expression (Yang et al. 2024). Some species of *Snodgrassella*, such as *S. alvi* in Am, are susceptible to glyphosate (Yang et al. 2024). These 2 genera had low abundances in some bee individuals, especially in Bsp. It is known that bumble bees sometimes lose their core microbes due to age and environmental stressors like pathogens (Hammer et al. 2021) and pesticides (Yang et al. 2024). However, we did not find any correlation between land use intensity (expressed as elevation) and the absence of the core bacteria in Bsp. As described in previous studies (Kwong and Moran 2016), our findings indicate that honey bees experience fewer losses of core microbes and are therefore less susceptible to environmental impacts.

Targeting Aim 2, characterizing patterns of exclusive and shared bacteria, the OTUs shared by wild and honey bees emerged as much more abundant (98.6%) than the exclusive ones (1.4% wild bees, <0.1% honey bees). Considering the low number of OTUs exclusive to Am (28) and the high abundance of OTUs shared by Bsp and Am (97.9%), this may reflect that the by far most abundant social bee groups of Bsp (71 bee individuals) and Am (100 bee individuals) shared their characteristic genera *Gilliamella* and *Snodgrassella*. It is known that bumble bees and honey bees share OTUs and thus bacterial species, but not necessarily the same strains of these species; different strains of 1 bacterial species co-evolve with their host bees; differentiation at the strain level could therefore reveal different patterns, as already known for, amongst others, *Snodgrassella* (Powell et al. 2016). As both *Gilliamella* and *Snodgrassella* are widespread in social bees, co-evolution and differentiation of strains could happen in these hosts to a particular extent. Looking only at the exclusive OTUs, there were many more of them in wild bees (347) than in Am (28). This might be because the group of wild bees consisted of 15 species, each with a different ecology, which in turn results in different bacterial communities; moreover, the sociality of bumble bees additionally facilitates specialization of gut microbes as co-evolution is promoted by favorable conditions within the nest, including assured host availability and various transmission routes (Kwong et al. 2017). The high OTU counts for Bsp also indicate this.

The microbial community structures were diverse, also within species, thus partly reducing species-specificity (Voulgari-Kokota et al. 2019). The number of OTUs observed was most variable in Bsp, followed by Am. These 2 bee groups also had the greatest sample sizes. For Bsp, the variability in OTU counts is in line with the large distances among individuals in the NMDS plot (Fig. 2C). Beyond this, the variety within some Bsp species was higher than among different Bsp species and even among Am samples (Supplementary Fig. S1). This justifies the analysis of Bsp at the genus level. Furthermore, even some individuals of the same sampling sites and thus same foraging habitats differed clearly in the NMDS plot (Supplementary Fig. S2). For Am, the large variability of the number of OTUs observed is not in line with the compact cluster of all Am individuals in the NMDS plot, which indicates their similarity in terms of bacteriobiota profile (Fig. 2C). Probably, all Am share their dominant bacteria species, but some Am individuals have some additional rare bacteria species. The reason for the generally lower bacterial diversity in Am compared with Bsp could be different breeding and overwintering conditions that differ across bumble bee nests; even within 1 nest, workers have different foraging preferences (Amiet and Krebs 2019). Depending on their role in the colony, they might share their gut microbes with some selected offspring through their social stomach. Contrasting this, different honey bee hives provide very similar conditions (Copeland et al. 2022). This is additionally illustrated by 2 further bacterial genera that were characteristic for Bsp: *Alkanindiges* is known to inhabit ants (Koto et al. 2020) and occurs in the phyllosphere of plants (Li et al. 2024). It is therefore not surprising that ground-nesting bumble bees also acquire it. Nevertheless, it had not been found in bumble bees before. Another genus specific to Bsp was *Pedobacter*, which is also a novel find for bumble bees. *Pedobacter* also lives in the phyllosphere of plants (Humphrey et al. 2014).

Compared with Bsp and Am, the bee groups Ah, Av, and Oc had fewer observed OTUs, possibly due to a lack of core microbiome in these non-social bees (Voulgari-Kokota et al. 2019) and the subsequent dominance of a few genera in most individuals. The high proportion of Firmicutes in Oc and their presence in Ah and Am were mainly due to *Spiroplasma*, the most abundant genus in Oc, also present in Ah and Am individuals. While it is known for Oc (Hettiarachchi et al. 2023), it had not been known for *A. hattorfiana*. In many insects, *Spiroplasma* is an important endosymbiont, just as *Wolbachia* (Eleftherianos et al. 2013, Hettiarachchi et al. 2023), which occurred dominantly in Ah and Av. In Andrenidae, *Wolbachia* potentially may play a crucial role in evolution and even speciation (McLaughlin et al. 2023). Consistent with our findings, *Wolbachia* has been known from *A. vaga* and *A. hattorfiana* but not from *O. cornuta* (Gerth et al. 2015, Hettiarachchi et al. 2023). Both *Wolbachia* and *Spiroplasma* can dominate the microbiome and simultaneously deliver the same positive effects as the outcompeted bacteria before (Hedges et al. 2008). Nevertheless, it might expose bees to a higher risk of disturbance due to the dominance of one group instead of a more diverse and thus stable bacterial composition.

For Oc, in addition to *Spiroplasma*, our data also revealed *Stenotrophomonas* as exclusive. Surprisingly, it is known to be important for health in larvae of honey bees and an antagonist of foulbrood (Ye et al. 2021). Also in *O. cornuta*, it was common (Hettiarachchi et al. 2023) and potentially plays an important role in the immune response there, too. Also, for Ah, we found another exclusive genus that might play an important role as an endosymbiont: *Asaia* is an emerging symbiont in bees (Crotti et al. 2010), but it had not been known from *Andrena*.

Regarding aim 3, identifying a potential relationship between honey bee density and bacterial diversity of wild bees and honey bees, *Commensalibacter* shows interesting patterns. It is also an important endosymbiont in insects, known among others from *Drosophila* and bees generally but not yet from Ah (Yang et al. 2024). Most of the 10 Ah individuals harbored this genus as well as many Am individuals. Exchange of *Commensalibacter* may have occurred between Ah and other insects in their environment, potentially including Am. This could also be a reason for the higher numbers of OTUs observed in Ah compared with Av and Oc. The oligolectic foraging of Ah on *K. arvensis*, which was one of the dominant blossoms at the time of sampling, enhanced the probability that the very abundant Am visited the same flower as Ah and transmitted their microbes there. Nevertheless, different foraging behaviors of the polylectic Oc, Bsp, and Am and the oligolectic Ah and Av seem to impact much less the bacterial composition than sociality. For instance, *Apibacter* (Bacteroidota) was present in Bsp and with lower abundance also in Am but not in Ah, Av, nor Oc.

Considering all wild bees together, our data revealed a significant, negative correlation between honey bee density and bacterial diversity of wild bees. Additionally, corresponding Am and wild bees became more similar with higher abundances of *A. mellifera* in close proximity. A possible explanation for this may be the transfer of the Am microbiome to wild bees, thereby reducing the diversity of wild bee gut microbes, a scenario, which is more likely in areas with high Am density. High honey bee densities have been known to negatively impact wild bees. Possibly, this not only applies to competition for floral resources (Mallinger et al. 2017, Wojcik et al. 2018, Iwasaki and Hogendoorn 2022) and pathogen transfer (Zbrozek et al. 2023) but may also affect the diversity and stability of wild bee microbiomes.

Concluding our study of bacteriobiota of wild bees and honey bees in an Alpine study system, we found some bacterial genera, which, to our knowledge, had not been recorded for *A. hattorfiana* (eg *Spiroplasma*), *A. vaga* (eg *Asaia*), *O. cornuta* (eg *Pseudomonas*), and even the well-studied *Bombus* spp. (eg *Alkanindiges*). Concerning honey bee competition, we found the first hints that high honey bee densities may correlate with diminished diversity of wild bee microbiomes. Clearly, from this starting point, additional analyses will be needed for more robust inferences and deeper insights.

Future analyses of bacteriobiota in this Alpine study system and beyond should include (i) characterizing microbes based on their functions in the host (Hammer et al. 2021, Peterson et al. 2024, Yang et al. 2024) to validate the usefulness of OTUs to resolve microbial communities; (ii) testing a larger sample per bee species over longer periods for more robust inferences than was possible here, particularly for the bumble bee species that had to be pooled (Supplementary Fig. S1); (iii) measuring density of bees generally and of honey bees especially in a way that integrates over longer periods of time in a habitat given, a methodological hurdle currently still to be taken; (iv) focusing on experimental honey bee exclusion or sampling of (high-elevation) areas where honey bees are naturally absent to achieve a stronger honeybee density gradient than achieved here; and (v) a broad monitoring of environmental factors like pathogens and pesticides in bees and their habitats, as well as a detailed observation of simultaneously foraging wild and honey bees and their interactions, to underpin correlational findings as in our study by mechanistic insight.

From a conservation-biological point of view, bumble bees as a social bee genus on the one hand occupy a similar niche as honey bees and are therefore most at risk (Wojcik et al. 2018). On the other hand, bumble bees’ socially transmitted core microbiome is more stable than that of solitary wild bees, and the intraspecific variation of microbiome composition is an advantage when facing environmental stressors. A less diverse microbiome, in turn, may lose its stability (Mockler et al. 2018). The co-evolution of bees and their gut microbes lead to specialization on different bacteria that partly play important roles in the immune response and thus the fitness of bees (Zhang and Zheng 2022). Landscape and land use, including beekeeping, may disturb these roles (Nguyen and Rehan 2023). To counter the biodiversity crisis, it is indispensable to consider wild bee fitness in landscaping, agriculture, and beekeeping.

## Supplementary Material

ieaf095_Supplementary_Data

## References

[ieaf095-B1] Amiet F , Krebs A. 2019. Bienen mitteleuropas. Gattungen, lebensweise, beobachtung. 3rd ed. Haupt Verlag.

[ieaf095-B2] Amiet F , Müller A, Praz C. 2017. Apidae 1—allgemeiner teil, gattungen, Apis, Bombus. CSCF & SEG (Fauna Helvetica 29, info fauna).

[ieaf095-B3] Andersen KS , Kirkegaard RH, Karst SM, et al 2018. ampvis2: an R package to analyse and visualise 16S rRNA amplicon data. 10.1101/299537

[ieaf095-B4] Beule L , Karlovsky P. 2020. Improved normalization of species count data in ecology by scaling with ranked subsampling (SRS): application to microbial communities. PeerJ. 8:e9593. 10.7717/peerj.9593 32832266 PMC7409812

[ieaf095-B5] Bokulich NA , Subramanian S, Faith JJ, et al 2013. Quality-filtering vastly improves diversity estimates from Illumina amplicon sequencing. Nat. Methods 10:57–59. 10.1038/nmeth.2276 23202435 PMC3531572

[ieaf095-B6] Brunson JC , Read QD. 2023. ggalluvial: Alluvial Plots in ‘ggplot2'. R package version 0.12.5. http://corybrunson.github.io/ggalluvial/

[ieaf095-B7] Cao Y , Dong Q, Wang D, et al 2022. microbiomeMarker: an R/Bioconductor package for microbiome marker identification and visualization. Bioinformatics 38:4027–4029. 10.1093/bioinformatics/btac438 35771644

[ieaf095-B8] Caporaso JG , Kuczynski J, Stombaugh J, et al 2010. QIIME allows analysis of high-throughput community sequencing data. Nat. Methods. 7:335–336. 10.1038/nmeth.f.303 20383131 PMC3156573

[ieaf095-B9] Caporaso JG , Lauber CL, Walters WA, et al 2011. Global patterns of 16S rRNA diversity at a depth of millions of sequences per sample. Proc. Natl. Acad. Sci. USA. 108 Suppl 1:4516–4522. 10.1073/pnas.1000080107 20534432 PMC3063599

[ieaf095-B10] Copeland DC , Anderson KE, Mott BM. 2022. Early queen development in honey bees: social context and queen breeder source affect gut microbiota and associated metabolism. Microbiol. Spectr. 10:e00383-22. 10.1128/spectrum.00383-22 35867384 PMC9430896

[ieaf095-B11] Crotti E , Rizzi A, Chouaia B, et al 2010. Acetic acid bacteria, newly emerging symbionts of insects. Appl. Environ. Microbiol. 76:6963–6970. 10.1128/AEM.01336-10 20851977 PMC2976266

[ieaf095-B12] Cunningham M , Tran L, McKee C, et al 2022. Honey bees as biomonitors of environmental contaminants, pathogens, and climate change. Ecol. Indic. 134:108457. 10.1016/j.ecolind.2021.108457

[ieaf095-B13] Dicks L , Breeze T, Ngo H, et al 2021. A global-scale expert assessment of drivers and risks associated with pollinator decline. Nat. Ecol. Evol. 5:1453–1461. 10.1038/s41559-021-01534-9 34400826

[ieaf095-B14] Edgar RC. 2013. UPARSE: highly accurate OTU sequences from microbial amplicon reads. Nat. Methods 10:996–998. 10.1038/nmeth.2604 23955772

[ieaf095-B15] Edgar RC , Haas BJ, Clemente JC, et al 2011. UCHIME improves sensitivity and speed of chimera detection. Bioinformatics. 27:2194–2200. 10.1093/bioinformatics/btr381 21700674 PMC3150044

[ieaf095-B16] Eleftherianos I , Atri J, Accetta J, et al 2013. Endosymbiotic bacteria in insects: guardians of the immune system? Front. Physiol. 4:46. 10.3389/fphys.2013.00046 23508299 PMC3597943

[ieaf095-B17] Esri. 2025. World imagery [data set]. https://www.arcgis.com/home/item.html?id=10df2279f9684e4a9f6a7f08febac2a9

[ieaf095-B18] Fürst MA , McMahon DP, Osborne JL, et al 2014. Disease associations between honeybees and bumblebees as a threat to wild pollinators. Nature 506:364–366. 10.1038/nature12977 24553241 PMC3985068

[ieaf095-B19] Gerth M , Saeed A, White JA, et al 2015. Extensive screen for bacterial endosymbionts reveals taxon-specific distribution patterns among bees (Hymenoptera, Anthophila). FEMS Microbiol. Ecol. 91:fiv047. 10.1093/femsec/fiv047 25914139

[ieaf095-B20] Gokcezade J , Gereben-Krenn B-A, Neumayer J. 2023. Feldbestimmungsschlüssel für die hummeln deutschlands, österreichs und der schweiz. 2. Quelle & Meyer.

[ieaf095-B21] Hammer T , Le E, Martin A, et al 2021. The gut microbiota of bumblebees. Insectes Soc. 68:287–301. 10.1007/s00040-021-00837-1 35342195 PMC8956082

[ieaf095-B22] Hedges LM , Brownlie JC, O'Neill SL, et al 2008. *Wolbachia* and virus protection in insects. Science. 322:702–702. 10.1126/science.1162418 18974344

[ieaf095-B23] Hettiarachchi A , Cnockaert M, Joossens M, et al 2023. The wild solitary bees *Andrena vaga*, *Anthophora plumipes*, *Colletes cunicularius*, and *Osmia cornuta* microbiota are host specific and dominated by endosymbionts and environmental microorganisms. Microb. Ecol. 86:3013–3026. 10.1007/s00248-023-02304-9 37794084

[ieaf095-B24] Hiebl J , Orlik A. 2023. Klimastatusbericht österreich 2022. Klimarückblick Tirol 2022. CCCA.

[ieaf095-B25] Humphrey PT , Nguyen TT, Villalobos MM, et al 2014. Diversity and abundance of phyllosphere bacteria are linked to insect herbivory. Mol. Ecol. 23:1497–1515. 10.1111/mec.12657 24383417

[ieaf095-B26] Iwasaki JM , Hogendoorn K. 2022. Mounting evidence that managed and introduced bees have negative impacts on wild bees: an updated review. Curr. Res. Insect Sci. 2:100043. 10.1016/j.cris.2022.100043 36003276 PMC9387436

[ieaf095-B27] Khalifa S , Elshafiey E, Shetaia A, et al 2021. Overview of bee pollination and its economic value for crop production. Insects. 12. 10.3390/insects12080688 PMC8396518 34442255

[ieaf095-B28] Kompass Wanderkarte. 2025. Interaktive onlinekarte. KOMPASS-Karten GmbH. https://www.kompass.de/wanderkarte

[ieaf095-B29] Koto A , Nobu MK, Miyazaki R. 2020. Deep sequencing uncovers caste-associated diversity of symbionts in the social ant *Camponotus japonicus*. mBio. 11. 10.1128/mbio.00408-20 PMC7175090 32317320

[ieaf095-B30] Kwong WK , Medina LA, Koch H, et al 2017. Dynamic microbiome evolution in social bees. Sci. Adv. 3:e1600513. 10.1126/sciadv.1600513 28435856 PMC5371421

[ieaf095-B31] Kwong WK , Moran NA. 2016. Gut microbial communities of social bees. Nat. Rev. Microbiol. 14:374–384. 10.1038/nrmicro.2016.43 27140688 PMC5648345

[ieaf095-B32] Lahti L , Shetty S. 2019. microbiome R package. https://bioconductor.org/packages/release/bioc/html/microbiome.html

[ieaf095-B33] Li W , Xiang L, Zheng P, et al 2024. Phyllosphere bacterial composition from *Brassica oleracea* and *Raphanus sativus*, the feeding food for *Plutella xylostella*. J. Asia-Pac. Entomol. 27:102238. 10.1016/j.aspen.2024.102238

[ieaf095-B34] Lignon V , Mas F, Jones E, et al 2024. The floral interface: a playground for interactions between insect pollinators, microbes, and plants. N. Z. J. Zool. 52:218–237. 10.1080/03014223.2024.2353285

[ieaf095-B35] Magoc T , Salzberg S. 2011. FLASH: fast length adjustment of short reads to improve genome assemblies. Bioinformatics. 27:2957–2963. 10.1093/bioinformatics/btr507 21903629 PMC3198573

[ieaf095-B36] Mallinger RE , Gaines-Day HR, Gratton C. 2017. Do managed bees have negative effects on wild bees?: a systematic review of the literature. PLoS One. 12:e0189268. 10.1371/journal.pone.0189268 29220412 PMC5722319

[ieaf095-B37] Martin M. 2011. Cutadapt removes adapter sequences from high-throughput sequencing reads. EMBnet. J. 17:10. 10.14806/ej.17.1.200

[ieaf095-B38] McLaughlin G , Gueuning M, Genoud D, et al 2023. Why are there so many species of mining bees (Hymenoptera, Andrenidae)? The possible roles of phenology and *Wolbachia* incompatibility in maintaining species boundaries in the *Andrena proxima*-complex. Syst. Entomol. 48:127–141. 10.1111/syen.12566

[ieaf095-B39] McMurdie P , Holmes S. 2013. phyloseq: an R package for reproducible interactive analysis and graphics of microbiome census data. PLoS One. 8:e61217. 10.1371/journal.pone.0061217 23630581 PMC3632530

[ieaf095-B40] Mockler BK , Kwong WK, Moran NA, et al 2018. Microbiome structure influences infection by the parasite *Crithidia bombi* in bumble bees. Appl. Environ. Microbiol. 84:e02335-17. 10.1128/AEM.02335-17 29374030 PMC5861814

[ieaf095-B41] Müller A , Praz C. 2024. Rote Liste der Bienen. Gefährdete Arten der Schweiz. Stand 2022. Bern Umwelt-Vollzug. http://www.bafu.admin.ch/uv-2402-d

[ieaf095-B42] Nguyen P , Rehan S. 2023. Environmental effects on bee microbiota. Microb. Ecol. 86:1487–1498. 10.1007/s00248-023-02226-6 37099156

[ieaf095-B43] Oksanen J , Simpson GL, Blanchet FG, et al 2001. vegan: community ecology package: 2.6-10. 10.32614/CRAN.package.vegan

[ieaf095-B44] Osterman J , Aizen MA, Biesmeijer JC, et al 2021. Global trends in the number and diversity of managed pollinator species. Agr. Ecosyst. Environ. 322:107653. 10.1016/j.agee.2021.107653

[ieaf095-B45] Peterson CB , Saha S, Do K-A. 2024. Analysis of microbiome data. Annu. Rev. Stat. Appl. 11:483–504. 10.1146/annurev-statistics-040522-120734 38962089 PMC11218911

[ieaf095-B46] Powell E , Ratnayeke N, Moran NA. 2016. Strain diversity and host specificity in a specialized gut symbiont of honeybees and bumblebees. Mol. Ecol. 25:4461–4471. 10.1111/mec.13787 27482856 PMC5650064

[ieaf095-B47] Quast C , Pruesse E, Yilmaz P, et al 2012. The SILVA ribosomal RNA gene database project: improved data processing and web-based tools. Nucleic Acids Res. 41:D590–D596. 10.1093/nar/gks1219 23193283 PMC3531112

[ieaf095-B48] R Core Team. 2024. R: a language and environment for statistical computing. https://www.R-project.org/

[ieaf095-B49] Russel J. 2021. Russel88, MicEco: various functions for analysis for microbial community data. https://github.com/Russel88/MicEco

[ieaf095-B50] Schloss PD , Westcott SL, Ryabin T, et al 2009. Introducing mothur: open-source, platform-independent, community-supported software for describing and comparing microbial communities. Appl. Environ. Microbiol. 75:7537–7541. 10.1128/AEM.01541-09 19801464 PMC2786419

[ieaf095-B51] Voulgari-Kokota A , McFrederick QS, Steffan-Dewenter I, et al 2019. Drivers, diversity, and functions of the solitary-bee microbiota. Trends Microbiol. 27:1034–1044. 10.1016/j.tim.2019.07.011 31451346

[ieaf095-B52] Westrich P. 2019. Die wildbienen deutschlands. 2nd ed. Eugen Ulmer.

[ieaf095-B53] Wickham H. 2016. ggplot2: elegant graphics for data analysis. Springer Publishing.

[ieaf095-B54] Wiesbauer H. 2020. Wilde bienen; biologie, lebensraumdynamik und gefährdung. 2nd ed. Eugen Ulmer.

[ieaf095-B55] Wojcik VA , Morandin LA, Davies Adams L, et al 2018. Floral resource competition between honey bees and wild bees: is there clear evidence and can we guide management and conservation? Environ. Entomol. 47:822–833. 10.1093/ee/nvy077 29873687

[ieaf095-B56] Yang C , Hu J, Su Q, et al 2024. A review on recent taxonomic updates of gut bacteria associated with social bees, with a curated genomic reference database. Insect Sci. 32:2. 10.1111/1744-7917.13365 38594229

[ieaf095-B57] Ye M-H , Fan S-H, Li X-Y, et al 2021. Microbiota dysbiosis in honeybee (*Apis mellifera* L.) larvae infected with brood diseases and foraging bees exposed to agrochemicals. R Soc. Open Sci. 8:201805. 10.1098/rsos.201805 33614099 PMC7890499

[ieaf095-B58] Zbrozek M , Fearon ML, Weise C, et al 2023. Honeybee visitation to shared flowers increases *Vairimorpha ceranae* prevalence in bumblebees. Ecol. Evol. 13:e10528. 10.1002/ece3.10528 37736280 PMC10511299

[ieaf095-B59] Zhang Z-J , Zheng H. 2022. Bumblebees with the socially transmitted microbiome: a novel model organism for gut microbiota research. Insect Sci. 29:958–976. 10.1111/1744-7917.13040 35567381

