# Supplementary material for: Bacterial communities of wild bee species and the western honey bee (Apis mellifera) (Hymenoptera: Apoidea): Alpine insights
Source: J Insect Sci. 2025 Nov 7;25(6):ieaf095. doi: 10.1093/jisesa/ieaf095 (PMC12598629; doi:10.1093/jisesa/ieaf095)
Supplement: ieaf095_Supplementary_Data [file ieaf095_supplementary_data.docx]

# Supplement

Sampling sites

Site 1 was located on the western outskirts of Innsbruck, directly at sand banks with willows at the river Inn, mainly surrounded by an airport and intensive fields and pastures. Site 2 was an avalanche basin with willows at the base of Nordkette, surrounded by mixed and spruce forests. In April, Av and Am were sampled at both sites as corresponding pairs of individuals.

Sites 3 and 4 were private gardens with apple trees in the settlement area in and around Innsbruck with other gardens around. Approximately 50 meters next to Site 3 were small-sized but intensively farmed fields and pastures. At both sites, Oc and Am were sampled as corresponding pairs of individuals in May.

Site 5 was an altitudinal transect at Nordkette (south exposed), ranging from 871 m a.s.l., at the edge of the settlement area, to 2233 m a.s.l. The lower section was characterized by extensively managed meadows below a cable car, flanked by a spruce forest. In higher elevations, above the tree line formed by *Pinus mugo*, alpine meadows dominated, with patches of willows and rocky areas. Some parts are used as cow paddocks and ski slopes, others are uncultivated. Every 50 meters of altitude, a corresponding pair of individuals of Bsp and Am was sampled. Two sampling rounds took place in May and June. Site 6 was a second altitudinal transect at the mountain Patscherkofel, south of Innsbruck (north exposed), ranging from 1024 m a.s.l to 2246 m a.s.l. Most of the transect was on meadows of a ski slope used as cow paddocks as well, neighbored by spruce forest. Between forest and rocky summit, there was a belt of *Rhododendron ferrugineum*, partly within meadows, partly in uncultivated areas. Two sampling rounds were conducted in June, with the flowering peak of *R. ferrugineum* in the second round. These blossoms served as the main nectar and pollen source for honey bees that had been brought up to the tree line with the *R. ferrugineum* spots above by bee keepers just before the second sampling round.

Site 7 was a small fertile meadow in a moderately urbanized area of Innsbruck. Site 8 was a fertile meadow surrounded by intensively used agricultural areas in the Inn Valley, 35 km west of Innsbruck. Many individuals of *Knautia arvensis* occurred on both meadows. At these two sites, Ah and Am were sampled as corresponding pairs of individuals in July.

Site 9 was located west of Obergurgl, 50 km south-west of Innsbruck (Kompass Wanderkarte 2025). Obergurgl is a small village in the Gurgler Tal, a small side valley of the Ötz Valley. It was a steep, south-east-exposed hillside with alpine meadows with shrubby parts at the base and steep, rocky parts higher up. Some parts are extensively used as cow and sheep pastures. Five Bsp individuals were sampled in the alpine to subnival upper part of this hillside, near little lakes, at 2800 m a.s.l. In higher elevations, no further Bsp were found. Five Am individuals were sampled at the shrubby base of the same slope at 1965 m a.s.l. as these were the first and highest individuals found at the descent. Site 10 was on the opposite side of the same valley, at the peak of Hohe Mut mountain at 2653 m a.s.l. On its alpine meadows, five Bsp individuals were sampled. Following the same procedure as at Site 9, five Am individuals were sampled at 1945 m a.s.l. next to a ski slope and pasture. Site 9 and 10 were sampled in July; the aim of sampling these sites was to sample bumble bees that have as much distance to honey bees as possible and thus to exclude the possibility of microbial exchange.

Site 11 was located at Alpenzoo in Innsbruck, where 10 Am individuals were sampled from one single beehive in August. The aim of sampling these individuals was to show the variance of microbial diversity and composition within one hive, in contrast to all other sampled Am individuals.

Tables

**Table S1:** Bee samples (n = 201) and sampling information; last column: number of *Apis mellifera* individuals observed simultaneously with the respective bee sample in the immediate surroundings. For site localization, see Fig. 1.

| **sample_ID** | **bee group_site_round** | **species** | **site_number** | **site_name** | **site_information** | **date_sampling** | **latitude** | **longitude** | **elevation [m]** | **Apis_simultaneous [n Ind]** |
| --- | --- | --- | --- | --- | --- | --- | --- | --- | --- | --- |
| 150003 | Av_1 | *Andrena vaga* | 1 | inn bank | with willows | 11.04.2022 | 47,25704703 | 11,33185297 | 586 | 50 |
| 150004 | Am_1 | *Apis mellifera* | 1 | inn bank | with willows | 11.04.2022 | 47,25704703 | 11,33185297 | 586 | 50 |
| 150009 | Av_1 | *Andrena vaga* | 1 | inn bank | with willows | 11.04.2022 | 47,25805596 | 11,32981298 | 590 | 10 |
| 150010 | Am_1 | *Apis mellifera* | 1 | inn bank | with willows | 11.04.2022 | 47,25805596 | 11,32981298 | 590 | 10 |
| 150013 | Av_1 | *Andrena vaga* | 1 | inn bank | with willows | 11.04.2022 | 47,25912902 | 11,32864103 | 592 | 10 |
| 150017 | Av_1 | *Andrena vaga* | 1 | inn bank | with willows | 11.04.2022 | 47,25773603 | 11,33044498 | 589 | 30 |
| 150018 | Am_1 | *Apis mellifera* | 1 | inn bank | with willows | 11.04.2022 | 47,25773603 | 11,33044498 | 589 | 30 |
| 150019 | Av_1 | *Andrena vaga* | 1 | inn bank | with willows | 11.04.2022 | 47,25773603 | 11,33044498 | 589 | 30 |
| 150020 | Am_1 | *Apis mellifera* | 1 | inn bank | with willows | 11.04.2022 | 47,25773603 | 11,33044498 | 589 | 30 |
| 150022 | Am_1 | *Apis mellifera* | 1 | inn bank | with willows | 11.04.2022 | 47,25933798 | 11,32844699 | 591 | 30 |
| 150024 | Av_2 | *Andrena vaga* | 2 | avalanche basin | with willows, southeast of Rauschbrunnen | 14.04.2022 | 47,274198 | 11,35051503 | 790 | 1 |
| 150025 | Am_2 | *Apis mellifera* | 2 | avalanche basin | with willows, southeast of Rauschbrunnen | 14.04.2022 | 47,274198 | 11,35051503 | 790 | 3 |
| 150026 | Am_2 | *Apis mellifera* | 2 | avalanche basin | with willows, southeast of Rauschbrunnen | 14.04.2022 | 47,274198 | 11,35051503 | 790 | 1 |
| 150029 | Av_2 | *Andrena vaga* | 2 | avalanche basin | with willows, southeast of Rauschbrunnen | 14.04.2022 | 47,27402299 | 11,35005101 | 775 | 0 |
| 150034 | Av_2 | *Andrena vaga* | 2 | avalanche basin | with willows, southeast of Rauschbrunnen | 14.04.2022 | 47,27402299 | 11,35005101 | 775 | 0 |
| 150035 | Am_2 | *Apis mellifera* | 2 | avalanche basin | with willows, southeast of Rauschbrunnen | 14.04.2022 | 47,27402299 | 11,35005101 | 775 | 1 |
| 150036 | Am_2 | *Apis mellifera* | 2 | avalanche basin | with willows, southeast of Rauschbrunnen | 14.04.2022 | 47,27402299 | 11,35005101 | 775 | 1 |
| 150038 | Av_2 | *Andrena vaga* | 2 | avalanche basin | with willows, southeast of Rauschbrunnen | 14.04.2022 | 47,27402299 | 11,35005101 | 775 | 1 |
| 150040 | Av_2 | *Andrena vaga* | 2 | avalanche basin | with willows, southeast of Rauschbrunnen | 14.04.2022 | 47,27402299 | 11,35005101 | 775 | 1 |
| 150041 | Am_2 | *Apis mellifera* | 2 | avalanche basin | with willows, southeast of Rauschbrunnen | 14.04.2022 | 47,27402299 | 11,35005101 | 775 | 1 |
| 150049 | Am_3 | *Apis mellifera* | 3 | Rum | garden with apple trees, in Murstrasse | 21.04.2022 | 47,28545297 | 11,45459597 | 620 | 10 |
| 150050 | Oc_3 | *Osmia cornuta* | 3 | Rum | garden with apple trees, in Murstrasse | 21.04.2022 | 47,28545297 | 11,45459597 | 620 | 40 |
| 150051 | Oc_3 | *Osmia cornuta* | 3 | Rum | garden with apple trees, in Murstrasse | 21.04.2022 | 47,28545297 | 11,45459597 | 620 | 40 |
| 150056 | Am_3 | *Apis mellifera* | 3 | Rum | garden with apple trees, in Murstrasse | 21.04.2022 | 47,28545297 | 11,45459597 | 620 | 10 |
| 150057 | Am_3 | *Apis mellifera* | 3 | Rum | garden with apple trees, in Murstrasse | 21.04.2022 | 47,28545297 | 11,45459597 | 620 | 10 |
| 150059 | Oc_3 | *Osmia cornuta* | 3 | Rum | garden with apple trees, in Murstrasse | 21.04.2022 | 47,28545297 | 11,45459597 | 620 | 10 |
| 150061 | Oc_3 | *Osmia cornuta* | 3 | Rum | garden with apple trees, in Murstrasse | 21.04.2022 | 47,28545297 | 11,45459597 | 620 | 10 |
| 150063 | Am_3 | *Apis mellifera* | 3 | Rum | garden with apple trees, in Murstrasse | 21.04.2022 | 47,28545297 | 11,45459597 | 620 | 10 |
| 150066 | Am_3 | *Apis mellifera* | 3 | Rum | garden with apple trees, in Murstrasse | 21.04.2022 | 47,28545297 | 11,45459597 | 620 | 10 |
| 150069 | Oc_3 | *Osmia cornuta* | 3 | Rum | garden with apple trees, in Murstrasse | 21.04.2022 | 47,28545297 | 11,45459597 | 620 | 10 |
| 150073 | Am_4 | *Apis mellifera* | 4 | Saggen | garden with apple trees, Rennweg 33 | 23.04.2022 | 47,27905104 | 11,40234796 | 581 | 10 |
| 150075 | Am_4 | *Apis mellifera* | 4 | Saggen | garden with apple trees, Rennweg 34 | 23.04.2022 | 47,27905104 | 11,40234796 | 581 | 20 |
| 150076 | Oc_4 | *Osmia cornuta* | 4 | Saggen | garden with apple trees, Rennweg 35 | 23.04.2022 | 47,27905104 | 11,40234796 | 581 | 20 |
| 150077 | Am_4 | *Apis mellifera* | 4 | Saggen | garden with apple trees, Rennweg 36 | 23.04.2022 | 47,27905104 | 11,40234796 | 581 | 20 |
| 150079 | Am_4 | *Apis mellifera* | 4 | Saggen | garden with apple trees, Rennweg 37 | 23.04.2022 | 47,27905104 | 11,40234796 | 581 | 20 |
| 150080 | Oc_4 | *Osmia cornuta* | 4 | Saggen | garden with apple trees, Rennweg 38 | 23.04.2022 | 47,27905104 | 11,40234796 | 581 | 30 |
| 150082 | Oc_4 | *Osmia cornuta* | 4 | Saggen | garden with apple trees, Rennweg 39 | 23.04.2022 | 47,27905104 | 11,40234796 | 581 | 30 |
| 150088 | Oc_4 | *Osmia cornuta* | 4 | Saggen | garden with apple trees, Rennweg 40 | 23.04.2022 | 47,27905104 | 11,40234796 | 581 | 30 |
| 150090 | Oc_4 | *Osmia cornuta* | 4 | Saggen | garden with apple trees, Rennweg 41 | 23.04.2022 | 47,27905104 | 11,40234796 | 581 | 30 |
| 150091 | Am_4 | *Apis mellifera* | 4 | Saggen | garden with apple trees, Rennweg 42 | 23.04.2022 | 47,27905104 | 11,40234796 | 581 | 30 |
| 150092 | Am_5_a | *Apis mellifera* | 5 | Seegrube | Hungerburg-Seilbahnsteig | 26.05.2022 | 47,28672501 | 11,397825 | 871 | 0 |
| 150093 | Am_5_a | *Apis mellifera* | 5 | Seegrube | Hungerburg-Seilbahnsteig | 26.05.2022 | 47,28880397 | 11,39657602 | 928 | 0 |
| 150094 | Am_5_a | *Apis mellifera* | 5 | Seegrube | Hungerburg-Seilbahnsteig | 26.05.2022 | 47,29053299 | 11,395107 | 961 | 0 |
| 150096 | Am_5_a | *Apis mellifera* | 5 | Seegrube | Hungerburg-Seilbahnsteig | 26.05.2022 | 47,29317999 | 11,3921 | 1062 | 1 |
| 150099 | Am_5_a | *Apis mellifera* | 5 | Seegrube | Hungerburg-Seilbahnsteig | 26.05.2022 | 47,29579004 | 11,39024299 | 1219 | 1 |
| 150100 | Am_5_a | *Apis mellifera* | 5 | Seegrube | Hungerburg-Seilbahnsteig | 27.05.2022 | 47,29892604 | 11,38724 | 1361 | 0 |
| 150103 | Am_5_a | *Apis mellifera* | 5 | Seegrube | Hungerburg-Seilbahnsteig | 27.05.2022 | 47,30085899 | 11,38499097 | 1520 | 0 |
| 150106 | Am_5_a | *Apis mellifera* | 5 | Seegrube | Hungerburg-Seilbahnsteig | 27.05.2022 | 47,30314901 | 11,38497798 | 1626 | 0 |
| 150107 | Am_5_a | *Apis mellifera* | 5 | Seegrube | Hungerburg-Seilbahnsteig | 27.05.2022 | 47,30375301 | 11,38271 | 1717 | 0 |
| 150109 | Am_5_a | *Apis mellifera* | 5 | Seegrube | Hungerburg-Seilbahnsteig | 27.05.2022 | 47,30509504 | 11,37917804 | 1834 | 0 |
| 150110 | Am_6_a | *Apis mellifera* | 6 | Patscherkofel | edge of spruce forest and ski slope | 05.06.2022 | 47,21771697 | 11,42086501 | 1024 | 0 |
| 150111 | Am_6_a | *Apis mellifera* | 6 | Patscherkofel | edge of spruce forest and ski slope | 05.06.2022 | 47,21823698 | 11,42568997 | 1076 | 1 |
| 150112 | Am_6_a | *Apis mellifera* | 6 | Patscherkofel | edge of spruce forest and ski slope | 05.06.2022 | 47,21803003 | 11,42944104 | 1152 | 10 |
| 150114 | Am_6_a | *Apis mellifera* | 6 | Patscherkofel | edge of spruce forest and ski slope | 05.06.2022 | 47,21749501 | 11,42974103 | 1186 | 0 |
| 150115 | Am_6_a | *Apis mellifera* | 6 | Patscherkofel | edge of spruce forest and ski slope | 05.06.2022 | 47,21284599 | 11,433906 | 1478 | 0 |
| 150116a | Am_6_b | *Apis mellifera* | 6 | Patscherkofel | spruce forest and ski slope | 19.06.2022 | 47,21762502 | 11,420949 | 1036 | 1 |
| 150117a | Am_6_b | *Apis mellifera* | 6 | Patscherkofel | spruce forest and ski slope | 19.06.2022 | 47,21817403 | 11,42575099 | 1072 | 2 |
| 150118 | Am_6_a | *Apis mellifera* | 6 | Patscherkofel | edge of spruce forest and ski slope | 06.06.2022 | 47,21307599 | 11,43752597 | 1566 | 0 |
| 150121 | Am_6_a | *Apis mellifera* | 6 | Patscherkofel | edge of spruce forest and ski slope | 06.06.2022 | 47,21018398 | 11,43993099 | 1678 | 0 |
| 150122 | Bsp_5_a | *Bombus lapidarius* | 5 | Seegrube | Hungerburg-Seilbahnsteig | 26.05.2022 | 47,28672501 | 11,397825 | 871 | 0 |
| 150123 | Bsp_5_a | *Bombus veteranus* | 5 | Seegrube | Hungerburg-Seilbahnsteig | 26.05.2022 | 47,28880397 | 11,39657602 | 928 | 0 |
| 150124 | Bsp_5_a | *Bombus terrestris Komplex* | 5 | Seegrube | Hungerburg-Seilbahnsteig | 26.05.2022 | 47,29053299 | 11,395107 | 961 | 0 |
| 150126 | Bsp_5_a | *Bombus terrestris Komplex* | 5 | Seegrube | Hungerburg-Seilbahnsteig | 26.05.2022 | 47,29317999 | 11,3921 | 1062 | 1 |
| 150130 | Bsp_5_a | *Bombus lapidarius* | 5 | Seegrube | Hungerburg-Seilbahnsteig | 26.05.2022 | 47,29610302 | 11,38992104 | 1225 | 0 |
| 150134 | Bsp_5_a | *Bombus lapidarius* | 5 | Seegrube | Hungerburg-Seilbahnsteig | 27.05.2022 | 47,29892604 | 11,38724 | 1361 | 1 |
| 150136 | Bsp_5_a | *Bombus lapidarius* | 5 | Seegrube | Hungerburg-Seilbahnsteig | 27.05.2022 | 47,30030998 | 11,38561802 | 1470 | 1 |
| 150139 | Bsp_5_a | *Bombus monticola* | 5 | Seegrube | Hungerburg-Seilbahnsteig | 27.05.2022 | 47,30314901 | 11,38497798 | 1626 | 0 |
| 150141 | Bsp_5_a | *Bombus lapidarius* | 5 | Seegrube | Hungerburg-Seilbahnsteig | 27.05.2022 | 47,30375301 | 11,38271 | 1717 | 0 |
| 150145 | Bsp_5_a | *Bombus jonellus* | 5 | Seegrube | Hungerburg-Seilbahnsteig | 27.05.2022 | 47,305031 | 11,37916203 | 1833 | 0 |
| 150147 | Bsp_5_a | *Bombus jonellus* | 5 | Seegrube | Hungerburg-Seilbahnsteig | 27.05.2022 | 47,30509504 | 11,37917804 | 1834 | 0 |
| 150148 | Bsp_5_a | *Bombus monticola* | 5 | Seegrube | Hungerburg-Seilbahnsteig | 27.05.2022 | 47,30575997 | 11,378697 | 1884 | 0 |
| 150149 | Bsp_5_a | *Bombus monticola* | 5 | Seegrube | Hungerburg-Seilbahnsteig | 27.05.2022 | 47,30541104 | 11,37561799 | 1945 | 0 |
| 150150 | Bsp_5_a | *Bombus monticola* | 5 | Seegrube | Hungerburg-Seilbahnsteig | 27.05.2022 | 47,30631997 | 11,37509597 | 1972 | 0 |
| 150151 | Bsp_5_a | *Bombus jonellus* | 5 | Seegrube | Hungerburg-Seilbahnsteig | 27.05.2022 | 47,30734197 | 11,37390004 | 2004 | 0 |
| 150152 | Bsp_6_a | *Bombus pratorum* | 6 | Patscherkofel | edge of spruce forest and ski slope | 05.06.2022 | 47,21771697 | 11,42086501 | 1024 | 0 |
| 150153 | Bsp_6_a | *Bombus pratorum* | 6 | Patscherkofel | edge of spruce forest and ski slope | 05.06.2022 | 47,21823698 | 11,42568997 | 1076 | 1 |
| 150154 | Bsp_6_a | *Bombus terrestris Komplex* | 6 | Patscherkofel | edge of spruce forest and ski slope | 05.06.2022 | 47,21803003 | 11,42944104 | 1152 | 10 |
| 150155 | Bsp_6_a | *Bombus lapidarius* | 6 | Patscherkofel | edge of spruce forest and ski slope | 05.06.2022 | 47,21749501 | 11,42974103 | 1186 | 1 |
| 150156 | Bsp_6_a | *Bombus lapidarius* | 6 | Patscherkofel | edge of spruce forest and ski slope | 05.06.2022 | 47,21749501 | 11,42974103 | 1186 | 1 |
| 150158 | Bsp_6_a | *Bombus pratorum* | 6 | Patscherkofel | edge of spruce forest and ski slope | 05.06.2022 | 47,21152199 | 11,42860201 | 1384 | 0 |
| 150161 | Bsp_6_a | *Bombus terrestris Komplex* | 6 | Patscherkofel | edge of spruce forest and ski slope | 05.06.2022 | 47,21284599 | 11,433906 | 1478 | 0 |
| 150163 | Bsp_6_a | *Bombus lapidarius* | 6 | Patscherkofel | edge of spruce forest and ski slope | 06.06.2022 | 47,21307599 | 11,43752597 | 1566 | 0 |
| 150165 | Bsp_6_a | *Bombus lapidarius* | 6 | Patscherkofel | edge of spruce forest and ski slope | 06.06.2022 | 47,210303 | 11,43990903 | 1676 | 0 |
| 150166 | Bsp_6_a | *Bombus terrestris Komplex* | 6 | Patscherkofel | edge of spruce forest and ski slope | 06.06.2022 | 47,20962298 | 11,44468898 | 1801 | 0 |
| 150168 | Bsp_6_a | *Bombus lapidarius* | 6 | Patscherkofel | edge of spruce forest and ski slope | 06.06.2022 | 47,20352397 | 11,45951103 | 2091 | 0 |
| 150169 | Bsp_6_a | *Bombus monticola* | 6 | Patscherkofel | edge of spruce forest and ski slope | 06.06.2022 | 47,20438303 | 11,45900602 | 2124 | 0 |
| 150170 | Bsp_6_a | *Bombus wurflenii* | 6 | Patscherkofel | edge of spruce forest and ski slope | 06.06.2022 | 47,20349397 | 11,45952302 | 2091 | 0 |
| 150171 | Bsp_5_b | *Bombus lapidarius* | 5 | Seegrube | Hungerburg-Seilbahnsteig | 11.06.2022 | 47,28672501 | 11,397825 | 871 | 0 |
| 150173 | Bsp_5_b | *Bombus terrestris Komplex* | 5 | Seegrube | Hungerburg-Seilbahnsteig | 11.06.2022 | 47,29053299 | 11,395107 | 961 | 0 |
| 150176 | Bsp_5_b | *Bombus hortorum/ruderatus* | 5 | Seegrube | Hungerburg-Seilbahnsteig | 11.06.2022 | 47,29335803 | 11,39226998 | 1074 | 0 |
| 150177 | Bsp_5_b | *Bombus humilis/pascuorum* | 5 | Seegrube | Hungerburg-Seilbahnsteig | 11.06.2022 | 47,29415003 | 11,39200302 | 1130 | 0 |
| 150182 | Bsp_5_b | *Bombus lapidarius* | 5 | Seegrube | Hungerburg-Seilbahnsteig | 11.06.2022 | 47,29893601 | 11,386938 | 1373 | 0 |
| 150184 | Bsp_5_b | *Bombus lapidarius* | 5 | Seegrube | Hungerburg-Seilbahnsteig | 11.06.2022 | 47,29613202 | 11,38982004 | 1224 | 0 |
| 150187 | Bsp_5_b | *Bombus lapidarius* | 5 | Seegrube | Hungerburg-Seilbahnsteig | 11.06.2022 | 47,30115504 | 11,38467497 | 1535 | 0 |
| 150190 | Bsp_5_b | *Bombus lapidarius* | 5 | Seegrube | alpine meadows above Seegrube | 12.06.2022 | 47,30626498 | 11,375045 | 1970 | 0 |
| 150194 | Am_6_a | *Apis mellifera* | 6 | Patscherkofel | edge of spruce forest and ski slope | 06.06.2022 | 47,20958401 | 11,44551996 | 1807 | 0 |
| 150196 | Am_6_a | *Apis mellifera* | 6 | Patscherkofel | edge of spruce forest and ski slope | 06.06.2022 | 47,20932199 | 11,449295 | 1919 | 0 |
| 150197 | Am_5_b | *Apis mellifera* | 5 | Seegrube | Hungerburg-Seilbahnsteig | 11.06.2022 | 47,28672501 | 11,397825 | 871 | 1 |
| 150199 | Am_5_b | *Apis mellifera* | 5 | Seegrube | Hungerburg-Seilbahnsteig | 11.06.2022 | 47,29053299 | 11,395107 | 961 | 0 |
| 150202 | Am_5_b | *Apis mellifera* | 5 | Seegrube | Hungerburg-Seilbahnsteig | 11.06.2022 | 47,29335803 | 11,39226998 | 1074 | 0 |
| 150203 | Am_5_b | *Apis mellifera* | 5 | Seegrube | Hungerburg-Seilbahnsteig | 11.06.2022 | 47,29405498 | 11,39155601 | 1128 | 0 |
| 150205 | Am_5_b | *Apis mellifera* | 5 | Seegrube | Hungerburg-Seilbahnsteig | 11.06.2022 | 47,29613202 | 11,38982004 | 1224 | 0 |
| 150208 | Am_5_b | *Apis mellifera* | 5 | Seegrube | Hungerburg-Seilbahnsteig | 11.06.2022 | 47,29893601 | 11,386938 | 1373 | 0 |
| 150211 | Am_5_b | *Apis mellifera* | 5 | Seegrube | Hungerburg-Seilbahnsteig | 11.06.2022 | 47,30101498 | 11,38497496 | 1521 | 0 |
| 150214 | Am_5_b | *Apis mellifera* | 5 | Seegrube | alpine meadows above Seegrube | 12.06.2022 | 47,30626498 | 11,375045 | 1970 | 0 |
| 150216 | Am_5_b | *Apis mellifera* | 5 | Seegrube | alpine meadows above Seegrube | 12.06.2022 | 47,30806198 | 11,37447302 | 2065 | 0 |
| 150217 | Am_5_b | *Apis mellifera* | 5 | Seegrube | alpine meadows above Seegrube | 12.06.2022 | 47,30886203 | 11,37438702 | 2126 | 0 |
| 150218 | Am_5_b | *Apis mellifera* | 5 | Seegrube | alpine meadows above Seegrube | 12.06.2022 | 47,309648 | 11,37368697 | 2198 | 0 |
| 150219 | Am_5_b | *Apis mellifera* | 5 | Seegrube | alpine meadows above Seegrube | 12.06.2022 | 47,30493704 | 11,37841503 | 1880 | 0 |
| 150220 | Am_5_b | *Apis mellifera* | 5 | Seegrube | alpine meadows above Seegrube | 12.06.2022 | 47,30457201 | 11,379316 | 1827 | 2 |
| 150222 | Am_5_b | *Apis mellifera* | 5 | Seegrube | alpine meadows above Seegrube | 12.06.2022 | 47,30355604 | 11,38071402 | 1732 | 0 |
| 150224 | Am_5_b | *Apis mellifera* | 5 | Seegrube | alpine meadows above Seegrube | 12.06.2022 | 47,30317198 | 11,38486298 | 1634 | 2 |
| 150225 | Am_6_b | *Apis mellifera* | 6 | Patscherkofel | spruce forest and ski slope | 19.06.2022 | 47,21824201 | 11,429147 | 1138 | 0 |
| 150226 | Am_6_b | *Apis mellifera* | 6 | Patscherkofel | spruce forest and ski slope | 19.06.2022 | 47,21766399 | 11,42959502 | 1176 | 0 |
| 150228 | Am_6_b | *Apis mellifera* | 6 | Patscherkofel | spruce forest and ski slope | 19.06.2022 | 47,21383397 | 11,42617403 | 1282 | 0 |
| 150230 | Am_6_b | *Apis mellifera* | 6 | Patscherkofel | spruce forest and ski slope | 19.06.2022 | 47,21181904 | 11,42762301 | 1360 | 0 |
| 150232 | Am_6_b | *Apis mellifera* | 6 | Patscherkofel | spruce forest and ski slope | 19.06.2022 | 47,212761 | 11,43371497 | 1470 | 0 |
| 150236 | Am_6_b | *Apis mellifera* | 6 | Patscherkofel | spruce forest and ski slope | 19.06.2022 | 47,21216102 | 11,44389404 | 1722 | 1 |
| 150237 | Bsp_5_b | *Bombus terrestris Komplex* | 5 | Seegrube | alpine meadows above Seegrube | 12.06.2022 | 47,30806198 | 11,37447302 | 2065 | 0 |
| 150238 | Bsp_5_b | *Bombus jonellus* | 5 | Seegrube | alpine meadows above Seegrube | 12.06.2022 | 47,30905004 | 11,37388101 | 2125 | 0 |
| 150240 | Bsp_5_b | *Bombus jonellus* | 5 | Seegrube | alpine meadows above Seegrube | 12.06.2022 | 47,310338 | 11,37336703 | 2233 | 0 |
| 150241 | Bsp_5_b | *Bombus hortorum* | 5 | Seegrube | alpine meadows above Seegrube | 12.06.2022 | 47,30978496 | 11,37377498 | 2209 | 0 |
| 150242 | Bsp_5_b | *Bombus wurflenii* | 5 | Seegrube | alpine meadows above Seegrube | 12.06.2022 | 47,30493704 | 11,37841503 | 1880 | 0 |
| 150243 | Bsp_5_b | *Bombus monticola* | 5 | Seegrube | alpine meadows above Seegrube | 12.06.2022 | 47,30457201 | 11,379316 | 1827 | 2 |
| 150245 | Bsp_5_b | *Bombus wurflenii* | 5 | Seegrube | alpine meadows above Seegrube | 12.06.2022 | 47,30355604 | 11,38071402 | 1732 | 0 |
| 150247 | Bsp_5_b | *Bombus lapidarius* | 5 | Seegrube | alpine meadows above Seegrube | 12.06.2022 | 47,30317198 | 11,38486298 | 1634 | 4 |
| 150248 | Bsp_6_b | *Bombus pratorum* | 6 | Patscherkofel | spruce forest and ski slope | 19.06.2022 | 47,21762502 | 11,420949 | 1036 | 0 |
| 150249 | Bsp_6_b | *Bombus pratorum* | 6 | Patscherkofel | spruce forest and ski slope | 19.06.2022 | 47,21817403 | 11,42575099 | 1072 | 2 |
| 150250 | Bsp_6_b | *Bombus humilis/pascuorum* | 6 | Patscherkofel | spruce forest and ski slope | 19.06.2022 | 47,21824201 | 11,429147 | 1138 | 0 |
| 150252 | Bsp_6_b | *Bombus lapidarius* | 6 | Patscherkofel | spruce forest and ski slope | 19.06.2022 | 47,21766399 | 11,42959502 | 1176 | 0 |
| 150254 | Bsp_6_b | *Bombus humilis/pascuorum* | 6 | Patscherkofel | spruce forest and ski slope | 19.06.2022 | 47,21281799 | 11,42596196 | 1299 | 0 |
| 150257 | Bsp_6_b | *Bombus humilis/pascuorum* | 6 | Patscherkofel | spruce forest and ski slope | 19.06.2022 | 47,211697 | 11,42797404 | 1370 | 0 |
| 150260 | Bsp_6_b | *Bombus lapidarius* | 6 | Patscherkofel | spruce forest and ski slope | 19.06.2022 | 47,212761 | 11,43371497 | 1470 | 0 |
| 150263 | Bsp_6_b | *Bombus lapidarius* | 6 | Patscherkofel | spruce forest and ski slope | 19.06.2022 | 47,21281003 | 11,43810097 | 1591 | 0 |
| 150267 | Bsp_6_b | *Bombus lapidarius* | 6 | Patscherkofel | spruce forest and ski slope | 19.06.2022 | 47,21236596 | 11,44445999 | 1740 | 1 |
| 150269 | Bsp_6_b | *Bombus lapidarius* | 6 | Patscherkofel | spruce forest and ski slope | 19.06.2022 | 47,21172298 | 11,44724404 | 1820 | 1 |
| 150271 | Bsp_6_b | *Bombus ruderarius* | 6 | Patscherkofel | subalpine shrub zone and ski slope | 21.06.2022 | 47,21012397 | 11,45054298 | 1929 | 5 |
| 150273 | Bsp_6_b | *Bombus pratorum* | 6 | Patscherkofel | subalpine shrub zone and ski slope | 21.06.2022 | 47,20967897 | 11,45407202 | 2026 | 10 |
| 150275 | Bsp_6_b | *Bombus terrestris Komplex* | 6 | Patscherkofel | subalpine shrub zone and ski slope | 21.06.2022 | 47,20902904 | 11,45644099 | 2132 | 1 |
| 150277 | Bsp_6_b | *Bombus hortorum/ruderatus* | 6 | Patscherkofel | subalpine shrub zone and ski slope | 21.06.2022 | 47,20876199 | 11,45716502 | 2175 | 4 |
| 150279 | Bsp_6_b | *Bombus lapidarius* | 6 | Patscherkofel | subalpine shrub zone and ski slope | 21.06.2022 | 47,208475 | 11,45956803 | 2234 | 0 |
| 150280 | Bsp_6_b | *Bombus lapidarius* | 6 | Patscherkofel | subalpine shrub zone and ski slope | 21.06.2022 | 47,20875302 | 11,46244604 | 2243 | 1 |
| 150281 | Bsp_6_b | *Bombus lapidarius* | 6 | Patscherkofel | subalpine shrub zone and ski slope | 21.06.2022 | 47,20875302 | 11,46244604 | 2243 | 1 |
| 150283 | Bsp_9 | *Bombus sichelii/pyrenaeus* | 9 | Obergurgl Gurgler Schartl | alpine meadow, above Seenplatte | 19.07.2022 | 46,89103204 | 11,00271299 | 2800 | 0 |
| 150289 | Am_6_b | *Apis mellifera* | 6 | Patscherkofel | spruce forest and ski slope | 19.06.2022 | 47,21172298 | 11,44724404 | 1820 | 0 |
| 150291 | Am_6_b | *Apis mellifera* | 6 | Patscherkofel | subalpine shrub zone and ski slope | 21.06.2022 | 47,21012397 | 11,45054298 | 1929 | 4 |
| 150293 | Am_6_b | *Apis mellifera* | 6 | Patscherkofel | subalpine shrub zone and ski slope | 21.06.2022 | 47,20967897 | 11,45407202 | 2026 | 10 |
| 150296 | Am_6_b | *Apis mellifera* | 6 | Patscherkofel | subalpine shrub zone and ski slope | 21.06.2022 | 47,20902904 | 11,45644099 | 2132 | 1 |
| 150297 | Am_6_b | *Apis mellifera* | 6 | Patscherkofel | subalpine shrub zone and ski slope | 21.06.2022 | 47,20876199 | 11,45716502 | 2175 | 0 |
| 150298 | Am_6_b | *Apis mellifera* | 6 | Patscherkofel | subalpine shrub zone and ski slope | 21.06.2022 | 47,208475 | 11,45956803 | 2234 | 0 |
| 150299 | Am_6_b | *Apis mellifera* | 6 | Patscherkofel | subalpine shrub zone and ski slope | 21.06.2022 | 47,20875302 | 11,46244604 | 2243 | 0 |
| 150300 | Am_6_b | *Apis mellifera* | 6 | Patscherkofel | subalpine shrub zone and ski slope | 21.06.2022 | 47,20875302 | 11,46244604 | 2243 | 0 |
| 150302 | Ah_7 | *Andrena hattorfiana* | 7 | Hötting | fertile meadow, settlement edge, forest edge | 16.07.2022 | 47,26518502 | 11,36291798 | 604 | 0 |
| 150303 | Ah_7 | *Andrena hattorfiana* | 7 | Hötting | fertile meadow, settlement edge, forest edge | 16.07.2022 | 47,26518502 | 11,36291798 | 604 | 0 |
| 150304 | Am_7 | *Apis mellifera* | 7 | Hötting | fertile meadow, settlement edge, forest edge | 16.07.2022 | 47,26518502 | 11,36291798 | 604 | 0 |
| 150307 | Am_7 | *Apis mellifera* | 7 | Hötting | fertile meadow, settlement edge, forest edge | 16.07.2022 | 47,26518502 | 11,36291798 | 604 | 0 |
| 150308 | Am_7 | *Apis mellifera* | 7 | Hötting | fertile meadow, settlement edge, forest edge | 16.07.2022 | 47,26518502 | 11,36291798 | 604 | 0 |
| 150309 | Am_7 | *Apis mellifera* | 7 | Hötting | fertile meadow, settlement edge, forest edge | 16.07.2022 | 47,26518502 | 11,36291798 | 604 | 1 |
| 150310 | Am_7 | *Apis mellifera* | 7 | Hötting | fertile meadow, settlement edge, forest edge | 16.07.2022 | 47,26518502 | 11,36291798 | 604 | 1 |
| 150312 | Ah_7 | *Andrena hattorfiana* | 7 | Hötting | fertile meadow, settlement edge, forest edge | 16.07.2022 | 47,26501697 | 11,36411098 | 598 | 0 |
| 150313 | Ah_7 | *Andrena hattorfiana* | 7 | Hötting | fertile meadow, settlement edge, forest edge | 16.07.2022 | 47,26501697 | 11,36411098 | 598 | 0 |
| 150314 | Ah_7 | *Andrena hattorfiana* | 7 | Hötting | fertile meadow, settlement edge, forest edge | 16.07.2022 | 47,26501697 | 11,36411098 | 598 | 0 |
| 150321 | Ah_8 | *Andrena hattorfiana* | 8 | Silz | fertile meadow, forest edge, near Silz | 16.07.2022 | 47,25937201 | 10,91609901 | 661 | 0 |
| 150322 | Ah_8 | *Andrena hattorfiana* | 8 | Silz | fertile meadow, forest edge, near Silz | 16.07.2022 | 47,25937201 | 10,91609901 | 661 | 0 |
| 150323 | Ah_8 | *Andrena hattorfiana* | 8 | Silz | fertile meadow, forest edge, near Silz | 16.07.2022 | 47,25937201 | 10,91609901 | 661 | 0 |
| 150327 | Am_8 | *Apis mellifera* | 8 | Silz | fertile meadow, forest edge, near Silz | 16.07.2022 | 47,25937201 | 10,91609901 | 661 | 1 |
| 150330 | Ah_8 | *Andrena hattorfiana* | 8 | Silz | fertile meadow, forest edge, near Silz | 16.07.2022 | 47,25937201 | 10,91609901 | 661 | 0 |
| 150334 | Ah_8 | *Andrena hattorfiana* | 8 | Silz | fertile meadow, forest edge, near Silz | 16.07.2022 | 47,25937201 | 10,91609901 | 661 | 0 |
| 150335 | Am_8 | *Apis mellifera* | 8 | Silz | fertile meadow, forest edge, near Silz | 16.07.2022 | 47,25937201 | 10,91609901 | 661 | 1 |
| 150337 | Am_8 | *Apis mellifera* | 8 | Silz | fertile meadow, forest edge, near Silz | 16.07.2022 | 47,25937201 | 10,91609901 | 661 | 1 |
| 150339 | Am_8 | *Apis mellifera* | 8 | Silz | fertile meadow, forest edge, near Silz | 16.07.2022 | 47,25937201 | 10,91609901 | 661 | 0 |
| 150340 | Am_8 | *Apis mellifera* | 8 | Silz | fertile meadow, forest edge, near Silz | 16.07.2022 | 47,25937201 | 10,91609901 | 661 | 0 |
| 150341 | Am_9 | *Apis mellifera* | 9 | Obergurgl Gurgler Schartl | alpine meadow, above Seenplatte | 19.07.2022 | 46,87758797 | 11,02540196 | 1965 | 0 |
| 150343 | Am_9 | *Apis mellifera* | 9 | Obergurgl Gurgler Schartl | alpine meadow, above Seenplatte | 19.07.2022 | 46,87758797 | 11,02540196 | 1965 | 0 |
| 150344 | Am_9 | *Apis mellifera* | 9 | Obergurgl Gurgler Schartl | alpine meadow, above Seenplatte | 19.07.2022 | 46,87758797 | 11,02540196 | 1965 | 0 |
| 150345 | Am_9 | *Apis mellifera* | 9 | Obergurgl Gurgler Schartl | alpine meadow, above Seenplatte | 19.07.2022 | 46,87758797 | 11,02540196 | 1965 | 0 |
| 150348 | Am_9 | *Apis mellifera* | 9 | Obergurgl Gurgler Schartl | alpine meadow, above Seenplatte | 19.07.2022 | 46,87758797 | 11,02540196 | 1965 | 0 |
| 150352 | Am_10 | *Apis mellifera* | 10 | Obergurgl Hohe Mut | on summit, alpine meadow | 19.07.2022 | 46,86824198 | 11,02794201 | 1945 | 0 |
| 150355 | Am_10 | *Apis mellifera* | 10 | Obergurgl Hohe Mut | on summit, alpine meadow | 19.07.2022 | 46,86824198 | 11,02794201 | 1945 | 0 |
| 150356 | Am_10 | *Apis mellifera* | 10 | Obergurgl Hohe Mut | on summit, alpine meadow | 19.07.2022 | 46,86824198 | 11,02794201 | 1945 | 0 |
| 150357 | Am_10 | *Apis mellifera* | 10 | Obergurgl Hohe Mut | on summit, alpine meadow | 19.07.2022 | 46,86840098 | 11,02749802 | 1932 | 0 |
| 150359 | Am_10 | *Apis mellifera* | 10 | Obergurgl Hohe Mut | on summit, alpine meadow | 19.07.2022 | 46,86824198 | 11,02794201 | 1945 | 0 |
| 150364 | Am_11 | *Apis mellifera* | 11 | Alpenzoo | all bees from same hive | 12.08.2022 | 47,28209199 | 11,39797697 | 678 | 50 |
| 150365 | Am_11 | *Apis mellifera* | 11 | Alpenzoo | all bees from same hive | 13.08.2022 | 47,28209199 | 11,39797697 | 678 | 50 |
| 150366 | Am_11 | *Apis mellifera* | 11 | Alpenzoo | all bees from same hive | 14.08.2022 | 47,28209199 | 11,39797697 | 678 | 50 |
| 150367 | Am_11 | *Apis mellifera* | 11 | Alpenzoo | all bees from same hive | 15.08.2022 | 47,28209199 | 11,39797697 | 678 | 50 |
| 150370 | Am_11 | *Apis mellifera* | 11 | Alpenzoo | all bees from same hive | 18.08.2022 | 47,28209199 | 11,39797697 | 678 | 50 |
| 150371 | Bsp_9 | *Bombus sichelii/pyrenaeus* | 9 | Obergurgl Gurgler Schartl | alpine meadow, above Seenplatte | 19.07.2022 | 46,89160603 | 11,00445801 | 2777 | 0 |
| 150375 | Bsp_9 | *Bombus sichelii/pyrenaeus* | 9 | Obergurgl Gurgler Schartl | alpine meadow, above Seenplatte | 19.07.2022 | 46,89113799 | 11,00555303 | 2736 | 0 |
| 150376 | Bsp_9 | *Bombus sichelii/pyrenaeus* | 9 | Obergurgl Gurgler Schartl | alpine meadow, above Seenplatte | 19.07.2022 | 46,89133999 | 11,00559301 | 2746 | 0 |
| 150377 | Bsp_9 | *Bombus sichelii/pyrenaeus* | 9 | Obergurgl Gurgler Schartl | alpine meadow, above Seenplatte | 19.07.2022 | 46,891026 | 11,00540299 | 2733 | 0 |
| 150380 | Bsp_10 | *Bombus terrestris Komplex* | 10 | Obergurgl Hohe Mut | on summit, alpine meadow | 19.07.2022 | 46,84832004 | 11,03000798 | 2650 | 0 |
| 150382 | Bsp_10 | *Bombus terrestris Komplex* | 10 | Obergurgl Hohe Mut | on summit, alpine meadow | 19.07.2022 | 46,84803899 | 11,03029598 | 2647 | 0 |
| 150385 | Bsp_10 | *Bombus lapidarius* | 10 | Obergurgl Hohe Mut | on summit, alpine meadow | 19.07.2022 | 46,848371 | 11,02977899 | 2643 | 0 |
| 150386 | Bsp_10 | *Bombus wurflenii* | 10 | Obergurgl Hohe Mut | on summit, alpine meadow | 19.07.2022 | 46,848371 | 11,02977899 | 2643 | 0 |
| 150388 | Bsp_10 | *Bombus sichelii/pyrenaeus* | 10 | Obergurgl Hohe Mut | on summit, alpine meadow | 19.07.2022 | 46,84907299 | 11,03024603 | 2642 | 0 |
| 150394 | Am_11 | *Apis mellifera* | 11 | Alpenzoo | all bees from same hive | 19.08.2022 | 47,28209199 | 11,39797697 | 678 | 50 |
| 150396 | Am_11 | *Apis mellifera* | 11 | Alpenzoo | all bees from same hive | 21.08.2022 | 47,28209199 | 11,39797697 | 678 | 50 |
| 150398 | Am_11 | *Apis mellifera* | 11 | Alpenzoo | all bees from same hive | 23.08.2022 | 47,28209199 | 11,39797697 | 678 | 50 |
| 150401 | Am_11 | *Apis mellifera* | 11 | Alpenzoo | all bees from same hive | 26.08.2022 | 47,28209199 | 11,39797697 | 678 | 50 |
| 150404 | Am_11 | *Apis mellifera* | 11 | Alpenzoo | all bees from same hive | 29.08.2022 | 47,28209199 | 11,39797697 | 678 | 50 |

**Table S2:** Detected Operational Taxonomic Units (OTUs, n = 997) and their taxonomic assignment. Sequencing data were filtered to remove mitochondria, chloroplasts, Archaea, and OTUs that occurred only once per bee sample and in less than 2.5% of bee samples (n = 201).

| **OTU** | **Kingdom** | **Phylum** | **Class** | **Order** | **Family** | **Genus** | **Species** |
| --- | --- | --- | --- | --- | --- | --- | --- |
| OTU00001 | Bacteria | Proteobacteria | Gammaproteobacteria | Enterobacterales | Orbaceae | Gilliamella | Gilliamella uncl. |
| OTU00002 | Bacteria | Proteobacteria | Gammaproteobacteria | Burkholderiales | Neisseriaceae | Snodgrassella | Snodgrassella uncl. |
| OTU00003 | Bacteria | Proteobacteria | Alphaproteobacteria | Rhizobiales | Rhizobiaceae | Bartonella | Bartonella_apis |
| OTU00004 | Bacteria | Proteobacteria | Gammaproteobacteria | Enterobacterales | Orbaceae | Frischella | Frischella_perrara |
| OTU00005 | Bacteria | Proteobacteria | Gammaproteobacteria | Enterobacterales | Enterobacterales uncl. | Enterobacterales uncl. | Enterobacterales uncl. |
| OTU00006 | Bacteria | Proteobacteria | Alphaproteobacteria | Acetobacterales | Acetobacteraceae | Commensalibacter | Commensalibacter_sp_AMU001 |
| OTU00007 | Bacteria | Proteobacteria | Alphaproteobacteria | Rickettsiales | Anaplasmataceae | Wolbachia | Wolbachia uncl. |
| OTU00009 | Bacteria | Firmicutes | Bacilli | Entomoplasmatales | Spiroplasmataceae | Spiroplasma | Spiroplasma uncl. |
| OTU00011 | Bacteria | Firmicutes | Bacilli | Lactobacillales | Lactobacillaceae | Lactobacillus | Lactobacillus_apis |
| OTU00013 | Bacteria | Firmicutes | Bacilli | Entomoplasmatales | Spiroplasmataceae | Spiroplasma | Spiroplasma_endosymbiont_of_Curculio_elephas |
| OTU00014 | Bacteria | Bacteroidota | Bacteroidia | Flavobacteriales | Weeksellaceae | Apibacter | Apibacter_sp_wkB309 |
| OTU00015 | Bacteria | Firmicutes | Bacilli | Entomoplasmatales | Spiroplasmataceae | Spiroplasma | Spiroplasma_citri |
| OTU00016 | Bacteria | Firmicutes | Bacilli | Lactobacillales | Lactobacillaceae | Lactobacillus | Lactobacillus_kullabergensis |
| OTU00017 | Bacteria | Proteobacteria | Gammaproteobacteria | Enterobacterales | Morganellaceae | Arsenophonus | Arsenophonus uncl. |
| OTU00018 | Bacteria | Proteobacteria | Gammaproteobacteria | Enterobacterales | Yersiniaceae | Yersiniaceae uncl. | Yersiniaceae uncl. |
| OTU00019 | Bacteria | Firmicutes | Bacilli | Lactobacillales | Lactobacillaceae | Bombilactobacillus | Bombilactobacillus uncl. |
| OTU00020 | Bacteria | Proteobacteria | Gammaproteobacteria | Pseudomonadales | Moraxellaceae | Acinetobacter | Acinetobacter uncl. |
| OTU00021 | Bacteria | Proteobacteria | Gammaproteobacteria | Pseudomonadales | Pseudomonadaceae | Pseudomonas | Pseudomonas uncl. |
| OTU00022 | Bacteria | Proteobacteria | Alphaproteobacteria | Rickettsiales | Anaplasmataceae | Wolbachia | Wolbachia_sp |
| OTU00024 | Bacteria | Actinobacteriota | Actinobacteria | Bifidobacteriales | Bifidobacteriaceae | Bifidobacterium | Bifidobacterium_asteroides |
| OTU00025 | Bacteria | unclassified | unclassified | unclassified | unclassified | unclassified | unclassified |
| OTU00026 | Bacteria | Proteobacteria | Gammaproteobacteria | Pseudomonadales | Pseudomonadaceae | Pseudomonas | Pseudomonas uncl. |
| OTU00028 | Bacteria | Proteobacteria | Alphaproteobacteria | Acetobacterales | Acetobacteraceae | Commensalibacter | Commensalibacter uncl. |
| OTU00029 | Bacteria | Actinobacteriota | Actinobacteria | Bifidobacteriales | Bifidobacteriaceae | Bombiscardovia | Bombiscardovia uncl. |
| OTU00030 | Bacteria | Firmicutes | Bacilli | Lactobacillales | Lactobacillaceae | Apilactobacillus | Lactobacillus_kunkeei |
| OTU00031 | Bacteria | Firmicutes | Bacilli | Lactobacillales | Lactobacillaceae | Bombilactobacillus | Lactobacillus_mellifer |
| OTU00032 | Bacteria | Firmicutes | Bacilli | Lactobacillales | Lactobacillaceae | Lactobacillaceae uncl. | Lactobacillaceae uncl. |
| OTU00034 | Bacteria | Proteobacteria | Gammaproteobacteria | Enterobacterales | Orbaceae | Candidatus_Schmidhempelia | Candidatus_Schmidhempelia uncl. |
| OTU00035 | Bacteria | Proteobacteria | Gammaproteobacteria | Pseudomonadales | Moraxellaceae | Enhydrobacter | Moraxella_osloensis |
| OTU00036 | Bacteria | unclassified | unclassified | unclassified | unclassified | unclassified | unclassified |
| OTU00037 | Bacteria | Proteobacteria | Alphaproteobacteria | Acetobacterales | Acetobacteraceae | Commensalibacter | Commensalibacter uncl. |
| OTU00038 | Bacteria | Proteobacteria | Gammaproteobacteria | Xanthomonadales | Xanthomonadaceae | Stenotrophomonas | Stenotrophomonas_rhizophila |
| OTU00039 | Bacteria | Firmicutes | Bacilli | Lactobacillales | Lactobacillaceae | Fructobacillus | Fructobacillus_tropaeoli |
| OTU00040 | Bacteria | Proteobacteria | Gammaproteobacteria | Pseudomonadales | Pseudomonadaceae | Pseudomonas | Pseudomonas uncl. |
| OTU00042 | Bacteria | unclassified | unclassified | unclassified | unclassified | unclassified | unclassified |
| OTU00043 | Bacteria | unclassified | unclassified | unclassified | unclassified | unclassified | unclassified |
| OTU00044 | Bacteria | unclassified | unclassified | unclassified | unclassified | unclassified | unclassified |
| OTU00045 | Bacteria | unclassified | unclassified | unclassified | unclassified | unclassified | unclassified |
| OTU00046 | Bacteria | Proteobacteria | Proteobacteria uncl. | Proteobacteria uncl. | Proteobacteria uncl. | Proteobacteria uncl. | Proteobacteria uncl. |
| OTU00047 | Bacteria | unclassified | unclassified | unclassified | unclassified | unclassified | unclassified |
| OTU00048 | Bacteria | Proteobacteria | Gammaproteobacteria | Pseudomonadales | Moraxellaceae | Alkanindiges | Moraxellaceae_bacterium_HYN0046 |
| OTU00049 | Bacteria | Proteobacteria | Alphaproteobacteria | Acetobacterales | Acetobacteraceae | Bombella | Bombella uncl. |
| OTU00050 | Bacteria | Firmicutes | Bacilli | Lactobacillales | Lactobacillaceae | Bombilactobacillus | Bombilactobacillus uncl. |
| OTU00051 | Bacteria | Firmicutes | Bacilli | Lactobacillales | Lactobacillaceae | Fructobacillus | Fructobacillus_fructosus |
| OTU00052 | Bacteria | unclassified | unclassified | unclassified | unclassified | unclassified | unclassified |
| OTU00053 | Bacteria | unclassified | unclassified | unclassified | unclassified | unclassified | unclassified |
| OTU00054 | Bacteria | Firmicutes | Bacilli | Lactobacillales | Streptococcaceae | Lactococcus | Lactococcus_lactis |
| OTU00055 | Bacteria | Proteobacteria | Alphaproteobacteria | Acetobacterales | Acetobacteraceae | Asaia | Asaia uncl. |
| OTU00056 | Bacteria | Proteobacteria | Gammaproteobacteria | Burkholderiales | Oxalobacteraceae | Janthinobacterium | Janthinobacterium uncl. |
| OTU00057 | Bacteria | unclassified | unclassified | unclassified | unclassified | unclassified | unclassified |
| OTU00059 | Bacteria | unclassified | unclassified | unclassified | unclassified | unclassified | unclassified |
| OTU00060 | Bacteria | Firmicutes | Bacilli | Lactobacillales | Streptococcaceae | Streptococcus | Streptococcus uncl. |
| OTU00062 | Bacteria | Proteobacteria | Proteobacteria uncl. | Proteobacteria uncl. | Proteobacteria uncl. | Proteobacteria uncl. | Proteobacteria uncl. |
| OTU00063 | Bacteria | Proteobacteria | Alphaproteobacteria | Acetobacterales | Acetobacteraceae | Acetobacteraceae uncl. | Acetobacteraceae uncl. |
| OTU00066 | Bacteria | unclassified | unclassified | unclassified | unclassified | unclassified | unclassified |
| OTU00067 | Bacteria | unclassified | unclassified | unclassified | unclassified | unclassified | unclassified |
| OTU00069 | Bacteria | Firmicutes | Negativicutes | Veillonellales-Selenomonadales | Selenomonadaceae | Megamonas | Megamonas uncl. |
| OTU00071 | Bacteria | unclassified | unclassified | unclassified | unclassified | unclassified | unclassified |
| OTU00072 | Bacteria | Firmicutes | Bacilli | Lactobacillales | Lactobacillaceae | Apilactobacillus | Lactobacillus_ozensis |
| OTU00073 | Bacteria | Bacteroidota | Bacteroidia | Sphingobacteriales | Sphingobacteriaceae | Pedobacter | Pedobacter uncl. |
| OTU00075 | Bacteria | unclassified | unclassified | unclassified | unclassified | unclassified | unclassified |
| OTU00076 | Bacteria | Actinobacteriota | Actinobacteria | Corynebacteriales | Corynebacteriaceae | Corynebacterium | Corynebacterium uncl. |
| OTU00078 | Bacteria | Bacteroidota | Bacteroidia | Bacteroidales | Prevotellaceae | Prevotella_7 | Prevotella_melaninogenica |
| OTU00079 | Bacteria | Proteobacteria | Gammaproteobacteria | Burkholderiales | Burkholderiaceae | Ralstonia | Ralstonia_pickettii |
| OTU00080 | Bacteria | Proteobacteria | Gammaproteobacteria | Pseudomonadales | Moraxellaceae | Acinetobacter | Acinetobacter uncl. |
| OTU00084 | Bacteria | unclassified | unclassified | unclassified | unclassified | unclassified | unclassified |
| OTU00085 | Bacteria | Firmicutes | Bacilli | Lactobacillales | Streptococcaceae | Streptococcus | Streptococcus uncl. |
| OTU00086 | Bacteria | unclassified | unclassified | unclassified | unclassified | unclassified | unclassified |
| OTU00087 | Bacteria | Bacteroidota | Bacteroidia | Bacteroidales | Dysgonomonadaceae | Dysgonomonas | Dysgonomonas uncl. |
| OTU00088 | Bacteria | Proteobacteria | Alphaproteobacteria | Sphingomonadales | Sphingomonadaceae | Sphingomonas | Sphingomonas_faeni |
| OTU00089 | Bacteria | Bacteroidota | Bacteroidia | Sphingobacteriales | Sphingobacteriaceae | Pedobacter | Pedobacter uncl. |
| OTU00090 | Bacteria | unclassified | unclassified | unclassified | unclassified | unclassified | unclassified |
| OTU00091 | Bacteria | unclassified | unclassified | unclassified | unclassified | unclassified | unclassified |
| OTU00092 | Bacteria | Proteobacteria | Gammaproteobacteria | Enterobacterales | Enterobacterales uncl. | Enterobacterales uncl. | Enterobacterales uncl. |
| OTU00093 | Bacteria | unclassified | unclassified | unclassified | unclassified | unclassified | unclassified |
| OTU00095 | Bacteria | unclassified | unclassified | unclassified | unclassified | unclassified | unclassified |
| OTU00096 | Bacteria | Bacteroidota | Bacteroidia | Bacteroidales | Prevotellaceae | Prevotella | Prevotella uncl. |
| OTU00098 | Bacteria | Fusobacteriota | Fusobacteriia | Fusobacteriales | Fusobacteriaceae | Fusobacterium | Fusobacterium_periodonticum |
| OTU00100 | Bacteria | unclassified | unclassified | unclassified | unclassified | unclassified | unclassified |
| OTU00101 | Bacteria | unclassified | unclassified | unclassified | unclassified | unclassified | unclassified |
| OTU00103 | Bacteria | unclassified | unclassified | unclassified | unclassified | unclassified | unclassified |
| OTU00104 | Bacteria | unclassified | unclassified | unclassified | unclassified | unclassified | unclassified |
| OTU00105 | Bacteria | unclassified | unclassified | unclassified | unclassified | unclassified | unclassified |
| OTU00106 | Bacteria | unclassified | unclassified | unclassified | unclassified | unclassified | unclassified |
| OTU00107 | Bacteria | unclassified | unclassified | unclassified | unclassified | unclassified | unclassified |
| OTU00108 | Bacteria | unclassified | unclassified | unclassified | unclassified | unclassified | unclassified |
| OTU00109 | Bacteria | unclassified | unclassified | unclassified | unclassified | unclassified | unclassified |
| OTU00111 | Bacteria | unclassified | unclassified | unclassified | unclassified | unclassified | unclassified |
| OTU00112 | Bacteria | Proteobacteria | Alphaproteobacteria | Rickettsiales | Rickettsiaceae | Rickettsia | Rickettsia uncl. |
| OTU00113 | Bacteria | unclassified | unclassified | unclassified | unclassified | unclassified | unclassified |
| OTU00114 | Bacteria | Bacteroidota | Bacteroidia | Flavobacteriales | Weeksellaceae | Chryseobacterium | Chryseobacterium_balustinum |
| OTU00115 | Bacteria | unclassified | unclassified | unclassified | unclassified | unclassified | unclassified |
| OTU00117 | Bacteria | Synergistota | Synergistia | Synergistales | Synergistaceae | Pyramidobacter | Pyramidobacter_piscolens |
| OTU00118 | Bacteria | Proteobacteria | Gammaproteobacteria | Burkholderiales | Burkholderiaceae | Robbsia | Robbsia uncl. |
| OTU00119 | Bacteria | Actinobacteriota | Actinobacteria | Corynebacteriales | Nocardiaceae | Rhodococcus | Rhodococcus uncl. |
| OTU00120 | Bacteria | unclassified | unclassified | unclassified | unclassified | unclassified | unclassified |
| OTU00121 | Bacteria | Proteobacteria | Gammaproteobacteria | Enterobacterales | Morganellaceae | Xenorhabdus | Xenorhabdus_bovienii |
| OTU00122 | Bacteria | Proteobacteria | Gammaproteobacteria | Burkholderiales | Comamonadaceae | Delftia | Delftia_tsuruhatensis |
| OTU00123 | Bacteria | Actinobacteriota | Actinobacteria | Micrococcales | Micrococcaceae | Arthrobacter | Arthrobacter uncl. |
| OTU00124 | Bacteria | unclassified | unclassified | unclassified | unclassified | unclassified | unclassified |
| OTU00125 | Bacteria | Actinobacteriota | Actinobacteria | Corynebacteriales | Nocardiaceae | Gordonia | Gordonia uncl. |
| OTU00126 | Bacteria | Firmicutes | Negativicutes | Veillonellales-Selenomonadales | Veillonellaceae | Megasphaera | Megasphaera_elsdenii |
| OTU00128 | Bacteria | Proteobacteria | Alphaproteobacteria | Sphingomonadales | Sphingomonadaceae | Sphingomonas | Sphingomonas uncl. |
| OTU00129 | Bacteria | Proteobacteria | Gammaproteobacteria | Burkholderiales | Comamonadaceae | Comamonas | Comamonas_testosteroni |
| OTU00132 | Bacteria | Actinobacteriota | Actinobacteria | Corynebacteriales | Nocardiaceae | Rhodococcus | Rhodococcus_erythropolis |
| OTU00133 | Bacteria | Firmicutes | Bacilli | Staphylococcales | Staphylococcaceae | Staphylococcus | Staphylococcus_aureus |
| OTU00134 | Bacteria | unclassified | unclassified | unclassified | unclassified | unclassified | unclassified |
| OTU00135 | Bacteria | Proteobacteria | Gammaproteobacteria | Pseudomonadales | Moraxellaceae | Acinetobacter | Acinetobacter uncl. |
| OTU00136 | Bacteria | unclassified | unclassified | unclassified | unclassified | unclassified | unclassified |
| OTU00137 | Bacteria | Firmicutes | Bacilli | Bacillales | Bacillaceae | Bacillaceae uncl. | Bacillaceae uncl. |
| OTU00138 | Bacteria | Proteobacteria | Alphaproteobacteria | Rickettsiales | Anaplasmataceae | Wolbachia | alpha_proteobacterium_endosymbiont_of_Coelostomidia_montana |
| OTU00139 | Bacteria | unclassified | unclassified | unclassified | unclassified | unclassified | unclassified |
| OTU00140 | Bacteria | unclassified | unclassified | unclassified | unclassified | unclassified | unclassified |
| OTU00141 | Bacteria | Proteobacteria | Alphaproteobacteria | Rhizobiales | Beijerinckiaceae | 1174-901-12 | 1174-901-12 uncl. |
| OTU00142 | Bacteria | Proteobacteria | Gammaproteobacteria | Burkholderiales | Oxalobacteraceae | Massilia | Massilia uncl. |
| OTU00143 | Bacteria | unclassified | unclassified | unclassified | unclassified | unclassified | unclassified |
| OTU00144 | Bacteria | unclassified | unclassified | unclassified | unclassified | unclassified | unclassified |
| OTU00145 | Bacteria | Proteobacteria | Gammaproteobacteria | Pseudomonadales | Pseudomonadaceae | Pseudomonadaceae uncl. | Pseudomonadaceae uncl. |
| OTU00146 | Bacteria | Proteobacteria | Alphaproteobacteria | Rhizobiales | Beijerinckiaceae | 1174-901-12 | 1174-901-12 uncl. |
| OTU00147 | Bacteria | Firmicutes | Clostridia | Lachnospirales | Lachnospiraceae | Lachnoanaerobaculum | Lachnoanaerobaculum uncl. |
| OTU00148 | Bacteria | Proteobacteria | Alphaproteobacteria | Rhizobiales | Beijerinckiaceae | Beijerinckiaceae uncl. | Beijerinckiaceae uncl. |
| OTU00149 | Bacteria | Firmicutes | Negativicutes | Veillonellales-Selenomonadales | Veillonellaceae | Veillonella | Veillonella uncl. |
| OTU00150 | Bacteria | Proteobacteria | Alphaproteobacteria | Rhizobiales | Beijerinckiaceae | Methylobacterium-Methylorubrum | Methylobacterium-Methylorubrum uncl. |
| OTU00151 | Bacteria | Proteobacteria | Alphaproteobacteria | Acetobacterales | Acetobacteraceae | Saccharibacter | Saccharibacter uncl. |
| OTU00153 | Bacteria | Bacteroidota | Bacteroidia | Sphingobacteriales | Sphingobacteriaceae | Sphingobacterium | Sphingobacterium uncl. |
| OTU00155 | Bacteria | Proteobacteria | Alphaproteobacteria | Acetobacterales | Acetobacteraceae | Endobacter | Endobacter uncl. |
| OTU00156 | Bacteria | Proteobacteria | Alphaproteobacteria | Rhizobiales | Beijerinckiaceae | 1174-901-12 | 1174-901-12 uncl. |
| OTU00158 | Bacteria | Acidobacteriota | Acidobacteriae | Acidobacteriales | Acidobacteriaceae_(Subgroup_1) | Terriglobus | Terriglobus uncl. |
| OTU00161 | Bacteria | Proteobacteria | Alphaproteobacteria | Sphingomonadales | Sphingomonadaceae | Sphingomonas | Sphingomonas uncl. |
| OTU00162 | Bacteria | Proteobacteria | Gammaproteobacteria | Pseudomonadales | Moraxellaceae | Alkanindiges | Alkanindiges uncl. |
| OTU00164 | Bacteria | Bacteroidota | Bacteroidia | Bacteroidales | Prevotellaceae | Prevotella_9 | Prevotella_9 uncl. |
| OTU00165 | Bacteria | Proteobacteria | Gammaproteobacteria | Enterobacterales | Orbaceae | Gilliamella | Gilliamella uncl. |
| OTU00166 | Bacteria | Firmicutes | Bacilli | Lactobacillales | Lactobacillaceae | Lactobacillus | Lactobacillus uncl. |
| OTU00167 | Bacteria | Firmicutes | Bacilli | Lactobacillales | Lactobacillaceae | Holzapfelia | Lactobacillus_floricola |
| OTU00169 | Bacteria | Firmicutes | Clostridia | Lachnospirales | Lachnospiraceae | Lachnoclostridium | Lachnoclostridium_sp_YL32 |
| OTU00170 | Bacteria | unclassified | unclassified | unclassified | unclassified | unclassified | unclassified |
| OTU00171 | Bacteria | Proteobacteria | Gammaproteobacteria | Enterobacterales | Morganellaceae | Buchnera | Buchnera uncl. |
| OTU00172 | Bacteria | Actinobacteriota | Actinobacteria | Corynebacteriales | Nocardiaceae | Rhodococcus | Rhodococcus uncl. |
| OTU00174 | Bacteria | Firmicutes | Bacilli | Erysipelotrichales | Erysipelotrichaceae | Solobacterium | Solobacterium_moorei |
| OTU00175 | Bacteria | Proteobacteria | Alphaproteobacteria | Acetobacterales | Acetobacteraceae | Acidiphilium | Acidiphilium uncl. |
| OTU00176 | Bacteria | unclassified | unclassified | unclassified | unclassified | unclassified | unclassified |
| OTU00177 | Bacteria | Firmicutes | Bacilli | Bacillales | Bacillaceae | Bacillus | Bacillus_thermolactis |
| OTU00178 | Bacteria | unclassified | unclassified | unclassified | unclassified | unclassified | unclassified |
| OTU00180 | Bacteria | unclassified | unclassified | unclassified | unclassified | unclassified | unclassified |
| OTU00181 | Bacteria | Proteobacteria | Alphaproteobacteria | Rhizobiales | Beijerinckiaceae | Methylobacterium-Methylorubrum | Methylobacterium-Methylorubrum uncl. |
| OTU00182 | Bacteria | Proteobacteria | Alphaproteobacteria | Rhizobiales | Rhizobiaceae | Allorhizobium-Neorhizobium-Pararhizobium-Rhizobium | Allorhizobium-Neorhizobium-Pararhizobium-Rhizobium uncl. |
| OTU00183 | Bacteria | Proteobacteria | Gammaproteobacteria | Enterobacterales | Pasteurellaceae | Haemophilus | Haemophilus_parainfluenzae |
| OTU00184 | Bacteria | unclassified | unclassified | unclassified | unclassified | unclassified | unclassified |
| OTU00185 | Bacteria | unclassified | unclassified | unclassified | unclassified | unclassified | unclassified |
| OTU00186 | Bacteria | Proteobacteria | Gammaproteobacteria | Pseudomonadales | Moraxellaceae | Acinetobacter | Acinetobacter uncl. |
| OTU00188 | Bacteria | unclassified | unclassified | unclassified | unclassified | unclassified | unclassified |
| OTU00189 | Bacteria | Actinobacteriota | Coriobacteriia | Coriobacteriales | Atopobiaceae | Coriobacteriaceae_UCG-003 | Coriobacteriaceae_UCG-003 uncl. |
| OTU00190 | Bacteria | unclassified | unclassified | unclassified | unclassified | unclassified | unclassified |
| OTU00191 | Bacteria | unclassified | unclassified | unclassified | unclassified | unclassified | unclassified |
| OTU00192 | Bacteria | Proteobacteria | Gammaproteobacteria | Pseudomonadales | Moraxellaceae | Acinetobacter | Acinetobacter uncl. |
| OTU00194 | Bacteria | Proteobacteria | Gammaproteobacteria | Burkholderiales | Burkholderiaceae | Burkholderia-Caballeronia-Paraburkholderia | Paraburkholderia_diazotrophica |
| OTU00195 | Bacteria | unclassified | unclassified | unclassified | unclassified | unclassified | unclassified |
| OTU00196 | Bacteria | unclassified | unclassified | unclassified | unclassified | unclassified | unclassified |
| OTU00197 | Bacteria | Proteobacteria | Gammaproteobacteria | Enterobacterales | Orbaceae | Orbaceae uncl. | Orbaceae uncl. |
| OTU00198 | Bacteria | Firmicutes | Clostridia | Lachnospirales | Lachnospiraceae | Blautia | Blautia uncl. |
| OTU00199 | Bacteria | Proteobacteria | Gammaproteobacteria | Pseudomonadales | Pseudomonadaceae | Pseudomonas | Pseudomonas uncl. |
| OTU00201 | Bacteria | Actinobacteriota | Actinobacteria | Streptomycetales | Streptomycetaceae | Streptomyces | Streptomyces uncl. |
| OTU00202 | Bacteria | Firmicutes | Clostridia | Lachnospirales | Lachnospiraceae | Lachnospiraceae uncl. | Lachnospiraceae uncl. |
| OTU00203 | Bacteria | unclassified | unclassified | unclassified | unclassified | unclassified | unclassified |
| OTU00204 | Bacteria | Proteobacteria | Alphaproteobacteria | Rhodobacterales | Rhodobacteraceae | Rhodobacteraceae uncl. | Rhodobacteraceae uncl. |
| OTU00206 | Bacteria | Proteobacteria | Gammaproteobacteria | Burkholderiales | Oxalobacteraceae | Duganella | Duganella uncl. |
| OTU00208 | Bacteria | unclassified | unclassified | unclassified | unclassified | unclassified | unclassified |
| OTU00209 | Bacteria | Proteobacteria | Alphaproteobacteria | Sphingomonadales | Sphingomonadaceae | Sphingomonadaceae uncl. | Sphingomonadaceae uncl. |
| OTU00210 | Bacteria | Proteobacteria | Alphaproteobacteria | Rhizobiales | Beijerinckiaceae | Methylobacterium-Methylorubrum | Methylobacterium-Methylorubrum uncl. |
| OTU00211 | Bacteria | unclassified | unclassified | unclassified | unclassified | unclassified | unclassified |
| OTU00212 | Bacteria | Actinobacteriota | Actinobacteria | Actinomycetales | Actinomycetaceae | Actinomyces | Schaalia_odontolytica |
| OTU00214 | Bacteria | Firmicutes | Clostridia | Peptostreptococcales-Tissierellales | Family_XI | Anaerosalibacter | Anaerosalibacter_sp_ND1 |
| OTU00215 | Bacteria | Proteobacteria | Alphaproteobacteria | Caulobacterales | Caulobacteraceae | Brevundimonas | Brevundimonas uncl. |
| OTU00216 | Bacteria | Bacteroidota | Bacteroidia | Bacteroidales | Dysgonomonadaceae | Dysgonomonas | Dysgonomonas uncl. |
| OTU00217 | Bacteria | unclassified | unclassified | unclassified | unclassified | unclassified | unclassified |
| OTU00218 | Bacteria | Firmicutes | Clostridia | Lachnospirales | Lachnospiraceae | Oribacterium | Oribacterium_sinus |
| OTU00219 | Bacteria | unclassified | unclassified | unclassified | unclassified | unclassified | unclassified |
| OTU00220 | Bacteria | unclassified | unclassified | unclassified | unclassified | unclassified | unclassified |
| OTU00221 | Bacteria | Firmicutes | Bacilli | Lactobacillales | Lactobacillaceae | Apilactobacillus | Lactobacillus_kosoi |
| OTU00222 | Bacteria | unclassified | unclassified | unclassified | unclassified | unclassified | unclassified |
| OTU00224 | Bacteria | unclassified | unclassified | unclassified | unclassified | unclassified | unclassified |
| OTU00225 | Bacteria | unclassified | unclassified | unclassified | unclassified | unclassified | unclassified |
| OTU00227 | Bacteria | Actinobacteriota | Actinobacteria | Propionibacteriales | Nocardioidaceae | Marmoricola | Marmoricola uncl. |
| OTU00228 | Bacteria | Fusobacteriota | Fusobacteriia | Fusobacteriales | Leptotrichiaceae | Leptotrichia | Leptotrichia uncl. |
| OTU00229 | Bacteria | Bacteroidota | Bacteroidia | Sphingobacteriales | Sphingobacteriaceae | Pedobacter | Pedobacter_antarcticus |
| OTU00230 | Bacteria | Bacteroidota | Bacteroidia | Flavobacteriales | Weeksellaceae | Chryseobacterium | Chryseobacterium_chaponense |
| OTU00231 | Bacteria | unclassified | unclassified | unclassified | unclassified | unclassified | unclassified |
| OTU00232 | Bacteria | unclassified | unclassified | unclassified | unclassified | unclassified | unclassified |
| OTU00235 | Bacteria | Firmicutes | Clostridia | Oscillospirales | Ruminococcaceae | Subdoligranulum | Subdoligranulum uncl. |
| OTU00238 | Bacteria | Actinobacteriota | Actinobacteria | Propionibacteriales | Propionibacteriaceae | Cutibacterium | Cutibacterium_acnes |
| OTU00239 | Bacteria | Proteobacteria | Gammaproteobacteria | Enterobacterales | Morganellaceae | Buchnera | Buchnera uncl. |
| OTU00240 | Bacteria | Firmicutes | Clostridia | Peptostreptococcales-Tissierellales | Peptostreptococcaceae | Peptostreptococcus | Peptostreptococcus uncl. |
| OTU00241 | Bacteria | Firmicutes | Clostridia | Lachnospirales | Lachnospiraceae | Anaerostipes | Anaerostipes_hadrus |
| OTU00242 | Bacteria | Firmicutes | Clostridia | Oscillospirales | Ruminococcaceae | Faecalibacterium | Faecalibacterium_prausnitzii |
| OTU00243 | Bacteria | Firmicutes | Bacilli | Lactobacillales | Listeriaceae | Listeria | Listeria uncl. |
| OTU00246 | Bacteria | unclassified | unclassified | unclassified | unclassified | unclassified | unclassified |
| OTU00248 | Bacteria | Bacteroidota | Bacteroidia | Cytophagales | Amoebophilaceae | Candidatus_Cardinium | Candidatus_Cardinium uncl. |
| OTU00249 | Bacteria | Actinobacteriota | Actinobacteria | Corynebacteriales | Mycobacteriaceae | Mycobacterium | Mycobacterium uncl. |
| OTU00251 | Bacteria | Firmicutes | Bacilli | Lactobacillales | Lactobacillaceae | Leuconostoc | Leuconostoc_lactis |
| OTU00252 | Bacteria | Proteobacteria | Gammaproteobacteria | Xanthomonadales | Rhodanobacteraceae | Rhodanobacter | Rhodanobacter uncl. |
| OTU00256 | Bacteria | unclassified | unclassified | unclassified | unclassified | unclassified | unclassified |
| OTU00257 | Bacteria | unclassified | unclassified | unclassified | unclassified | unclassified | unclassified |
| OTU00258 | Bacteria | Firmicutes | Bacilli | Staphylococcales | Gemellaceae | Gemella | Gemella uncl. |
| OTU00259 | Bacteria | Bacteroidota | Bacteroidia | Bacteroidales | Bacteroidaceae | Bacteroides | Bacteroides uncl. |
| OTU00260 | Bacteria | Proteobacteria | Gammaproteobacteria | Burkholderiales | Hydrogenophilaceae | Hydrogenophilus | Hydrogenophilus_thermoluteolus |
| OTU00261 | Bacteria | Actinobacteriota | Actinobacteria | Corynebacteriales | Nocardiaceae | Rhodococcus | Rhodococcus_globerulus |
| OTU00263 | Bacteria | Bacteroidota | Bacteroidia | Bacteroidales | Bacteroidaceae | Bacteroides | Bacteroides_uniformis |
| OTU00265 | Bacteria | Bacteroidota | Bacteroidia | Cytophagales | Hymenobacteraceae | Hymenobacter | Hymenobacter uncl. |
| OTU00266 | Bacteria | Firmicutes | Clostridia | Lachnospirales | Lachnospiraceae | Fusicatenibacter | Fusicatenibacter uncl. |
| OTU00268 | Bacteria | unclassified | unclassified | unclassified | unclassified | unclassified | unclassified |
| OTU00269 | Bacteria | Firmicutes | Bacilli | Thermoactinomycetales | Thermoactinomycetaceae | Kroppenstedtia | Kroppenstedtia uncl. |
| OTU00270 | Bacteria | Actinobacteriota | Actinobacteria | Corynebacteriales | Corynebacteriales uncl. | Corynebacteriales uncl. | Corynebacteriales uncl. |
| OTU00271 | Bacteria | Proteobacteria | Gammaproteobacteria | Pseudomonadales | Pseudomonadaceae | Pseudomonas | Pseudomonas_citronellolis |
| OTU00272 | Bacteria | unclassified | unclassified | unclassified | unclassified | unclassified | unclassified |
| OTU00275 | Bacteria | unclassified | unclassified | unclassified | unclassified | unclassified | unclassified |
| OTU00278 | Bacteria | unclassified | unclassified | unclassified | unclassified | unclassified | unclassified |
| OTU00279 | Bacteria | Proteobacteria | Alphaproteobacteria | Acetobacterales | Acetobacteraceae | Acidiphilium | Acidiphilium uncl. |
| OTU00280 | Bacteria | Proteobacteria | Alphaproteobacteria | Acetobacterales | Acetobacteraceae | Acidocella | Acidocella_aluminiidurans |
| OTU00281 | Bacteria | unclassified | unclassified | unclassified | unclassified | unclassified | unclassified |
| OTU00285 | Bacteria | unclassified | unclassified | unclassified | unclassified | unclassified | unclassified |
| OTU00286 | Bacteria | unclassified | unclassified | unclassified | unclassified | unclassified | unclassified |
| OTU00290 | Bacteria | Proteobacteria | Alphaproteobacteria | Sphingomonadales | Sphingomonadaceae | Sphingomonadaceae uncl. | Sphingomonadaceae uncl. |
| OTU00293 | Bacteria | Bacteroidota | Bacteroidia | Chitinophagales | Chitinophagaceae | Arachidicoccus | Arachidicoccus uncl. |
| OTU00294 | Bacteria | Myxococcota | Polyangia | Haliangiales | Haliangiaceae | Haliangium | Haliangium uncl. |
| OTU00295 | Bacteria | Proteobacteria | Gammaproteobacteria | Pseudomonadales | Pseudomonadaceae | Pseudomonas | Gammaproteobacteria_bacterium_ESL0073 |
| OTU00298 | Bacteria | unclassified | unclassified | unclassified | unclassified | unclassified | unclassified |
| OTU00299 | Bacteria | unclassified | unclassified | unclassified | unclassified | unclassified | unclassified |
| OTU00300 | Bacteria | Proteobacteria | Gammaproteobacteria | Diplorickettsiales | Diplorickettsiaceae | Rickettsiella | Candidatus_Rickettsiella_viridis |
| OTU00301 | Bacteria | Proteobacteria | Gammaproteobacteria | Burkholderiales | Neisseriaceae | Neisseria | Neisseria_perflava |
| OTU00303 | Bacteria | unclassified | unclassified | unclassified | unclassified | unclassified | unclassified |
| OTU00304 | Bacteria | unclassified | unclassified | unclassified | unclassified | unclassified | unclassified |
| OTU00305 | Bacteria | Bacteroidota | Bacteroidia | Bacteroidales | Prevotellaceae | Alloprevotella | Prevotella_sp |
| OTU00306 | Bacteria | unclassified | unclassified | unclassified | unclassified | unclassified | unclassified |
| OTU00307 | Bacteria | unclassified | unclassified | unclassified | unclassified | unclassified | unclassified |
| OTU00309 | Bacteria | Proteobacteria | Alphaproteobacteria | Rickettsiales | SM2D12 | SM2D12 uncl. | SM2D12 uncl. |
| OTU00311 | Bacteria | unclassified | unclassified | unclassified | unclassified | unclassified | unclassified |
| OTU00312 | Bacteria | unclassified | unclassified | unclassified | unclassified | unclassified | unclassified |
| OTU00313 | Bacteria | Bacteroidota | Bacteroidia | Bacteroidales | Muribaculaceae | Muribaculaceae uncl. | Muribaculaceae uncl. |
| OTU00314 | Bacteria | unclassified | unclassified | unclassified | unclassified | unclassified | unclassified |
| OTU00316 | Bacteria | Firmicutes | Negativicutes | Acidaminococcales | Acidaminococcaceae | Phascolarctobacterium | Phascolarctobacterium_faecium |
| OTU00317 | Bacteria | Bdellovibrionota | Bdellovibrionia | Bacteriovoracales | Bacteriovoracaceae | Bacteriovorax | Bacteriovorax uncl. |
| OTU00318 | Bacteria | Actinobacteriota | Actinobacteria | Micrococcales | Microbacteriaceae | Microterricola | Microterricola_viridarii |
| OTU00319 | Bacteria | unclassified | unclassified | unclassified | unclassified | unclassified | unclassified |
| OTU00320 | Bacteria | Actinobacteriota | Actinobacteria | Micrococcales | Microbacteriaceae | Microbacteriaceae uncl. | Microbacteriaceae uncl. |
| OTU00322 | Bacteria | Proteobacteria | Gammaproteobacteria | Burkholderiales | Neisseriaceae | Snodgrassella | Snodgrassella uncl. |
| OTU00324 | Bacteria | Proteobacteria | Alphaproteobacteria | Caulobacterales | Caulobacteraceae | Brevundimonas | Brevundimonas_vesicularis |
| OTU00325 | Bacteria | Firmicutes | Clostridia | Clostridia_UCG-014 | Clostridia_UCG-014 uncl. | Clostridia_UCG-014 uncl. | Clostridia_UCG-014 uncl. |
| OTU00326 | Bacteria | unclassified | unclassified | unclassified | unclassified | unclassified | unclassified |
| OTU00327 | Bacteria | unclassified | unclassified | unclassified | unclassified | unclassified | unclassified |
| OTU00328 | Bacteria | unclassified | unclassified | unclassified | unclassified | unclassified | unclassified |
| OTU00329 | Bacteria | Bacteroidota | Bacteroidia | Bacteroidales | Prevotellaceae | Prevotella | Prevotella_pallens |
| OTU00330 | Bacteria | Actinobacteriota | Actinobacteria | Micrococcales | Dermacoccaceae | Flexivirga | Flexivirga uncl. |
| OTU00331 | Bacteria | Proteobacteria | Alphaproteobacteria | Caulobacterales | Caulobacteraceae | Brevundimonas | Brevundimonas_diminuta |
| OTU00334 | Bacteria | unclassified | unclassified | unclassified | unclassified | unclassified | unclassified |
| OTU00335 | Bacteria | Proteobacteria | Gammaproteobacteria | Enterobacterales | Enterobacterales uncl. | Enterobacterales uncl. | Enterobacterales uncl. |
| OTU00337 | Bacteria | unclassified | unclassified | unclassified | unclassified | unclassified | unclassified |
| OTU00338 | Bacteria | Bacteroidota | Bacteroidia | Flavobacteriales | Weeksellaceae | Chryseobacterium | Chryseobacterium_glaciei |
| OTU00340 | Bacteria | Proteobacteria | Proteobacteria uncl. | Proteobacteria uncl. | Proteobacteria uncl. | Proteobacteria uncl. | Proteobacteria uncl. |
| OTU00342 | Bacteria | unclassified | unclassified | unclassified | unclassified | unclassified | unclassified |
| OTU00343 | Bacteria | Proteobacteria | Alphaproteobacteria | Rhodobacterales | Rhodobacteraceae | Rhodobacteraceae uncl. | Rhodobacteraceae uncl. |
| OTU00344 | Bacteria | Proteobacteria | Gammaproteobacteria | Enterobacterales | Enterobacterales uncl. | Enterobacterales uncl. | Enterobacterales uncl. |
| OTU00345 | Bacteria | Firmicutes | Clostridia | Peptostreptococcales-Tissierellales | Family_XI | Parvimonas | Parvimonas uncl. |
| OTU00347 | Bacteria | unclassified | unclassified | unclassified | unclassified | unclassified | unclassified |
| OTU00348 | Bacteria | Proteobacteria | Gammaproteobacteria | Pseudomonadales | Moraxellaceae | Cavicella | Cavicella uncl. |
| OTU00349 | Bacteria | unclassified | unclassified | unclassified | unclassified | unclassified | unclassified |
| OTU00350 | Bacteria | Proteobacteria | Alphaproteobacteria | Rhizobiales | Rhizobiaceae | Rhizobiaceae uncl. | Rhizobiaceae uncl. |
| OTU00351 | Bacteria | Bacteroidota | Bacteroidia | Flavobacteriales | Weeksellaceae | Cloacibacterium | Cloacibacterium uncl. |
| OTU00352 | Bacteria | unclassified | unclassified | unclassified | unclassified | unclassified | unclassified |
| OTU00353 | Bacteria | unclassified | unclassified | unclassified | unclassified | unclassified | unclassified |
| OTU00354 | Bacteria | Actinobacteriota | Actinobacteria | Corynebacteriales | unidentified_Corynebacteriales | Tomitella | Tomitella uncl. |
| OTU00355 | Bacteria | Firmicutes | Bacilli | Lactobacillales | Lactobacillaceae | Lactobacillus | Lactobacillus uncl. |
| OTU00356 | Bacteria | Actinobacteriota | Coriobacteriia | Coriobacteriales | Atopobiaceae | Atopobium | Atopobium uncl. |
| OTU00357 | Bacteria | Proteobacteria | Alphaproteobacteria | Acetobacterales | Acetobacteraceae | Acetobacteraceae uncl. | Acetobacteraceae uncl. |
| OTU00358 | Bacteria | unclassified | unclassified | unclassified | unclassified | unclassified | unclassified |
| OTU00359 | Bacteria | Bacteroidota | Bacteroidia | Flavobacteriales | Weeksellaceae | Weeksellaceae uncl. | Weeksellaceae uncl. |
| OTU00360 | Bacteria | Bacteroidota | Bacteroidia | Bacteroidales | Tannerellaceae | Parabacteroides | Parabacteroides uncl. |
| OTU00361 | Bacteria | Actinobacteriota | Actinobacteria | Frankiales | Frankiaceae | Jatrophihabitans | Jatrophihabitans uncl. |
| OTU00363 | Bacteria | Chloroflexi | Chloroflexia | Kallotenuales | AKIW781 | AKIW781 uncl. | AKIW781 uncl. |
| OTU00364 | Bacteria | unclassified | unclassified | unclassified | unclassified | unclassified | unclassified |
| OTU00366 | Bacteria | unclassified | unclassified | unclassified | unclassified | unclassified | unclassified |
| OTU00368 | Bacteria | Firmicutes | Bacilli | Bacillales | Bacillaceae | Bacillus | Bacillus uncl. |
| OTU00370 | Bacteria | Firmicutes | Bacilli | Bacillales | Sporolactobacillaceae | Sporolactobacillus | Sporolactobacillus_inulinus |
| OTU00371 | Bacteria | Actinobacteriota | Actinobacteria | Micrococcales | Micrococcaceae | Micrococcus | Micrococcus_luteus |
| OTU00373 | Bacteria | Actinobacteriota | Actinobacteria | Corynebacteriales | Tsukamurellaceae | Tsukamurella | Tsukamurella uncl. |
| OTU00375 | Bacteria | Proteobacteria | Alphaproteobacteria | Rhizobiales | Rhizobiaceae | Rhizobiaceae uncl. | Rhizobiaceae uncl. |
| OTU00376 | Bacteria | Bacteroidota | Bacteroidia | Bacteroidales | Rikenellaceae | Rikenellaceae_RC9_gut_group | Rikenellaceae_RC9_gut_group uncl. |
| OTU00377 | Bacteria | unclassified | unclassified | unclassified | unclassified | unclassified | unclassified |
| OTU00378 | Bacteria | unclassified | unclassified | unclassified | unclassified | unclassified | unclassified |
| OTU00379 | Bacteria | unclassified | unclassified | unclassified | unclassified | unclassified | unclassified |
| OTU00380 | Bacteria | unclassified | unclassified | unclassified | unclassified | unclassified | unclassified |
| OTU00381 | Bacteria | unclassified | unclassified | unclassified | unclassified | unclassified | unclassified |
| OTU00382 | Bacteria | Actinobacteriota | Actinobacteria | Bifidobacteriales | Bifidobacteriaceae | Bifidobacterium | Bifidobacterium_animalis |
| OTU00384 | Bacteria | Proteobacteria | Alphaproteobacteria | Acetobacterales | Acetobacteraceae | Acetobacteraceae uncl. | Acetobacteraceae uncl. |
| OTU00385 | Bacteria | unclassified | unclassified | unclassified | unclassified | unclassified | unclassified |
| OTU00386 | Bacteria | unclassified | unclassified | unclassified | unclassified | unclassified | unclassified |
| OTU00387 | Bacteria | unclassified | unclassified | unclassified | unclassified | unclassified | unclassified |
| OTU00388 | Bacteria | unclassified | unclassified | unclassified | unclassified | unclassified | unclassified |
| OTU00390 | Bacteria | Proteobacteria | Proteobacteria uncl. | Proteobacteria uncl. | Proteobacteria uncl. | Proteobacteria uncl. | Proteobacteria uncl. |
| OTU00391 | Bacteria | Proteobacteria | Gammaproteobacteria | Burkholderiales | Hydrogenophilaceae | Tepidiphilus | Tepidiphilus uncl. |
| OTU00393 | Bacteria | Bacteroidota | Bacteroidia | Cytophagales | Spirosomaceae | Dyadobacter | Dyadobacter uncl. |
| OTU00394 | Bacteria | Actinobacteriota | Coriobacteriia | Coriobacteriales | Atopobiaceae | Olsenella | Olsenella uncl. |
| OTU00395 | Bacteria | Firmicutes | Bacilli | Bacillales | Bacillaceae | Bacillaceae uncl. | Bacillaceae uncl. |
| OTU00396 | Bacteria | Bacteroidota | Bacteroidia | Sphingobacteriales | Sphingobacteriaceae | Sphingobacteriaceae uncl. | Sphingobacteriaceae uncl. |
| OTU00397 | Bacteria | Proteobacteria | Alphaproteobacteria | Acetobacterales | Acetobacteraceae | Acetobacteraceae uncl. | Acetobacteraceae uncl. |
| OTU00399 | Bacteria | unclassified | unclassified | unclassified | unclassified | unclassified | unclassified |
| OTU00400 | Bacteria | Proteobacteria | Gammaproteobacteria | Enterobacterales | Hafniaceae | Hafnia-Obesumbacterium | Hafnia-Obesumbacterium uncl. |
| OTU00402 | Bacteria | Proteobacteria | Gammaproteobacteria | Pseudomonadales | Marinobacteraceae | Marinobacter | Marinobacter_hydrocarbonoclasticus |
| OTU00403 | Bacteria | Firmicutes | Clostridia | Monoglobales | Monoglobaceae | Monoglobus | Monoglobus uncl. |
| OTU00404 | Bacteria | Firmicutes | Bacilli | Lactobacillales | Lactobacillaceae | Lactobacillus | Lactobacillus uncl. |
| OTU00405 | Bacteria | Desulfobacterota | Desulfovibrionia | Desulfovibrionales | Desulfovibrionaceae | Bilophila | Bilophila uncl. |
| OTU00406 | Bacteria | Actinobacteriota | Coriobacteriia | Coriobacteriales | Eggerthellaceae | Enterorhabdus | Enterorhabdus uncl. |
| OTU00408 | Bacteria | Proteobacteria | Alphaproteobacteria | Acetobacterales | Acetobacteraceae | unidentified_Acetobacteraceae | unidentified_Acetobacteraceae uncl. |
| OTU00411 | Bacteria | Actinobacteriota | Actinobacteria | Corynebacteriales | Corynebacteriaceae | Corynebacterium | Corynebacterium_ureicelerivorans |
| OTU00413 | Bacteria | Proteobacteria | Gammaproteobacteria | Burkholderiales | Neisseriaceae | Neisseriaceae uncl. | Neisseriaceae uncl. |
| OTU00414 | Bacteria | Actinobacteriota | Actinobacteria | Propionibacteriales | Nocardioidaceae | Nocardioides | Nocardioides uncl. |
| OTU00415 | Bacteria | Actinobacteriota | Actinobacteria | Propionibacteriales | Nocardioidaceae | Nocardioides | Nocardioides uncl. |
| OTU00417 | Bacteria | Bacteroidota | Bacteroidia | Sphingobacteriales | env.OPS_17 | env.OPS_17 uncl. | env.OPS_17 uncl. |
| OTU00418 | Bacteria | Proteobacteria | Alphaproteobacteria | Acetobacterales | Acetobacteraceae | Acidiphilium | Acidiphilium uncl. |
| OTU00419 | Bacteria | unclassified | unclassified | unclassified | unclassified | unclassified | unclassified |
| OTU00421 | Bacteria | Actinobacteriota | Actinobacteria | Propionibacteriales | Nocardioidaceae | Aeromicrobium | Aeromicrobium uncl. |
| OTU00422 | Bacteria | unclassified | unclassified | unclassified | unclassified | unclassified | unclassified |
| OTU00424 | Bacteria | Acidobacteriota | Acidobacteriae | Acidobacteriales | Acidobacteriaceae_(Subgroup_1) | Granulicella | Granulicella uncl. |
| OTU00425 | Bacteria | Proteobacteria | Gammaproteobacteria | Pseudomonadales | Pseudomonadaceae | Pseudomonas | Pseudomonas_psychrotolerans |
| OTU00426 | Bacteria | Proteobacteria | Alphaproteobacteria | Sphingomonadales | Sphingomonadaceae | Sphingomonas | Sphingomonas uncl. |
| OTU00427 | Bacteria | unclassified | unclassified | unclassified | unclassified | unclassified | unclassified |
| OTU00428 | Bacteria | Bacteroidota | Bacteroidia | Sphingobacteriales | Sphingobacteriales uncl. | Sphingobacteriales uncl. | Sphingobacteriales uncl. |
| OTU00429 | Bacteria | Proteobacteria | Gammaproteobacteria | Xanthomonadales | Rhodanobacteraceae | Luteibacter | Luteibacter uncl. |
| OTU00431 | Bacteria | unclassified | unclassified | unclassified | unclassified | unclassified | unclassified |
| OTU00435 | Bacteria | Firmicutes | Bacilli | Lactobacillales | Streptococcaceae | Floricoccus | Floricoccus uncl. |
| OTU00436 | Bacteria | Proteobacteria | Alphaproteobacteria | Rickettsiales | Rickettsiaceae | Rickettsiaceae uncl. | Rickettsiaceae uncl. |
| OTU00438 | Bacteria | Firmicutes | Negativicutes | Acidaminococcales | Acidaminococcaceae | Acidaminococcus | Acidaminococcus uncl. |
| OTU00439 | Bacteria | Bacteroidota | Bacteroidia | Flavobacteriales | Flavobacteriaceae | Flavobacterium | Flavobacterium_hauense |
| OTU00440 | Bacteria | Firmicutes | Negativicutes | Veillonellales-Selenomonadales | Veillonellaceae | Dialister | Dialister uncl. |
| OTU00441 | Bacteria | Actinobacteriota | Actinobacteria | Corynebacteriales | Nocardiaceae | Rhodococcus | Rhodococcus uncl. |
| OTU00442 | Bacteria | Firmicutes | Clostridia | Oscillospirales | Oscillospiraceae | NK4A214_group | NK4A214_group uncl. |
| OTU00443 | Bacteria | Proteobacteria | Gammaproteobacteria | Salinisphaerales | Solimonadaceae | Alkanibacter | Sinobacteraceae_bacterium |
| OTU00444 | Bacteria | Actinobacteriota | Actinobacteria | Micrococcales | Brevibacteriaceae | Brevibacterium | Brevibacterium_iodinum |
| OTU00445 | Bacteria | unclassified | unclassified | unclassified | unclassified | unclassified | unclassified |
| OTU00447 | Bacteria | Proteobacteria | Gammaproteobacteria | Xanthomonadales | Xanthomonadaceae | Xanthomonadaceae uncl. | Xanthomonadaceae uncl. |
| OTU00449 | Bacteria | Bacteroidota | Bacteroidia | Bacteroidales | Porphyromonadaceae | Porphyromonas | Porphyromonas uncl. |
| OTU00450 | Bacteria | unclassified | unclassified | unclassified | unclassified | unclassified | unclassified |
| OTU00451 | Bacteria | Bacteroidota | Bacteroidia | Chitinophagales | Chitinophagaceae | Edaphobaculum | Edaphobaculum uncl. |
| OTU00453 | Bacteria | Firmicutes | Clostridia | Lachnospirales | Lachnospiraceae | Lachnoclostridium | Lachnoclostridium uncl. |
| OTU00456 | Bacteria | Bacteroidota | Bacteroidia | Flavobacteriales | Crocinitomicaceae | Crocinitomicaceae uncl. | Crocinitomicaceae uncl. |
| OTU00458 | Bacteria | Bacteroidota | Bacteroidia | Chitinophagales | Chitinophagaceae | Taibaiella | Taibaiella uncl. |
| OTU00460 | Bacteria | Firmicutes | Clostridia | Lachnospirales | Lachnospiraceae | Oribacterium | Oribacterium uncl. |
| OTU00461 | Bacteria | Proteobacteria | Alphaproteobacteria | Acetobacterales | Acetobacteraceae | Acetobacteraceae uncl. | Acetobacteraceae uncl. |
| OTU00463 | Bacteria | Firmicutes | Clostridia | Peptostreptococcales-Tissierellales | Anaerovoracaceae | [Eubacterium]_nodatum_group | [Eubacterium]_nodatum_group uncl. |
| OTU00467 | Bacteria | Firmicutes | Bacilli | Lactobacillales | Carnobacteriaceae | Carnobacterium | Carnobacterium uncl. |
| OTU00468 | Bacteria | Proteobacteria | Gammaproteobacteria | Burkholderiales | Alcaligenaceae | Alcaligenaceae uncl. | Alcaligenaceae uncl. |
| OTU00469 | Bacteria | Proteobacteria | Proteobacteria uncl. | Proteobacteria uncl. | Proteobacteria uncl. | Proteobacteria uncl. | Proteobacteria uncl. |
| OTU00470 | Bacteria | Proteobacteria | Alphaproteobacteria | Rhizobiales | Beijerinckiaceae | 1174-901-12 | 1174-901-12 uncl. |
| OTU00474 | Bacteria | Firmicutes | Clostridia | Oscillospirales | [Eubacterium]_coprostanoligenes_group | [Eubacterium]_coprostanoligenes_group uncl. | [Eubacterium]_coprostanoligenes_group uncl. |
| OTU00475 | Bacteria | unclassified | unclassified | unclassified | unclassified | unclassified | unclassified |
| OTU00476 | Bacteria | Proteobacteria | Gammaproteobacteria | Enterobacterales | Orbaceae | Gilliamella | Gilliamella_apicola |
| OTU00478 | Bacteria | Bacteroidota | Bacteroidia | Bacteroidales | Muribaculaceae | Muribaculaceae uncl. | Muribaculaceae uncl. |
| OTU00480 | Bacteria | Bacteroidota | Bacteroidia | Bacteroidales | Bacteroidaceae | Bacteroides | Bacteroides_plebeius |
| OTU00481 | Bacteria | Proteobacteria | Alphaproteobacteria | Rhizobiales | Xanthobacteraceae | Bradyrhizobium | Bradyrhizobium uncl. |
| OTU00482 | Bacteria | Bacteroidota | Bacteroidia | Cytophagales | Hymenobacteraceae | Hymenobacter | Hymenobacter uncl. |
| OTU00483 | Bacteria | unclassified | unclassified | unclassified | unclassified | unclassified | unclassified |
| OTU00485 | Bacteria | unclassified | unclassified | unclassified | unclassified | unclassified | unclassified |
| OTU00489 | Bacteria | Firmicutes | Bacilli | Entomoplasmatales | Entomoplasmataceae | Mesoplasma | Mesoplasma uncl. |
| OTU00490 | Bacteria | Proteobacteria | Alphaproteobacteria | Caulobacterales | Caulobacteraceae | Caulobacter | Caulobacter uncl. |
| OTU00491 | Bacteria | Proteobacteria | Gammaproteobacteria | Burkholderiales | Neisseriaceae | Snodgrassella | Snodgrassella uncl. |
| OTU00492 | Bacteria | Acidobacteriota | Acidobacteriae | Acidobacteriales | Acidobacteriales uncl. | Acidobacteriales uncl. | Acidobacteriales uncl. |
| OTU00493 | Bacteria | Proteobacteria | Gammaproteobacteria | Burkholderiales | Methylophilaceae | Methylotenera | Methylotenera uncl. |
| OTU00494 | Bacteria | Actinobacteriota | Actinobacteria | Corynebacteriales | Nocardiaceae | Williamsia | Williamsia uncl. |
| OTU00495 | Bacteria | unclassified | unclassified | unclassified | unclassified | unclassified | unclassified |
| OTU00496 | Bacteria | Proteobacteria | Alphaproteobacteria | Rhizobiales | Hyphomicrobiaceae | Hyphomicrobium | Hyphomicrobium_zavarzinii |
| OTU00497 | Bacteria | Proteobacteria | Gammaproteobacteria | Pseudomonadales | Pseudomonadaceae | Pseudomonas | Pseudomonas uncl. |
| OTU00498 | Bacteria | Proteobacteria | Gammaproteobacteria | Burkholderiales | Alcaligenaceae | Verticiella | Verticiella uncl. |
| OTU00499 | Bacteria | unclassified | unclassified | unclassified | unclassified | unclassified | unclassified |
| OTU00501 | Bacteria | Firmicutes | Clostridia | Lachnospirales | Lachnospiraceae | Roseburia | Roseburia_intestinalis |
| OTU00502 | Bacteria | Bacteroidota | Bacteroidia | Flavobacteriales | Flavobacteriaceae | Flavobacterium | Flavobacterium_sp_140616W15 |
| OTU00506 | Bacteria | Firmicutes | Bacilli | Lactobacillales | Carnobacteriaceae | Atopostipes | Atopostipes uncl. |
| OTU00507 | Bacteria | Proteobacteria | Alphaproteobacteria | Sphingomonadales | Sphingomonadaceae | Novosphingobium | Novosphingobium uncl. |
| OTU00509 | Bacteria | Bacteroidota | Bacteroidia | Cytophagales | Spirosomaceae | Flectobacillus | Flectobacillus uncl. |
| OTU00510 | Bacteria | Actinobacteriota | Actinobacteria | Corynebacteriales | Nocardiaceae | Nocardia | Nocardia uncl. |
| OTU00511 | Bacteria | Bacteroidota | Bacteroidia | Cytophagales | Hymenobacteraceae | Hymenobacter | Hymenobacter uncl. |
| OTU00513 | Bacteria | Proteobacteria | Alphaproteobacteria | Rhizobiales | Rhizobiaceae | Aureimonas | Aureimonas uncl. |
| OTU00514 | Bacteria | Firmicutes | Bacilli | Erysipelotrichales | Erysipelotrichaceae | Catenisphaera | Catenisphaera uncl. |
| OTU00515 | Bacteria | Bacteroidota | Bacteroidia | Bacteroidales | Bacteroidaceae | Bacteroides | Bacteroides_sp_Marseille-P3166 |
| OTU00516 | Bacteria | unclassified | unclassified | unclassified | unclassified | unclassified | unclassified |
| OTU00518 | Bacteria | Firmicutes | Negativicutes | Veillonellales-Selenomonadales | Veillonellaceae | Allisonella | Allisonella uncl. |
| OTU00519 | Bacteria | Proteobacteria | Alphaproteobacteria | Rhizobiales | Rhizobiaceae | Phyllobacterium | Phyllobacterium uncl. |
| OTU00525 | Bacteria | Bacteroidota | Bacteroidia | Bacteroidales | Prevotellaceae | Prevotellaceae_UCG-001 | Prevotellaceae_UCG-001 uncl. |
| OTU00526 | Bacteria | Firmicutes | unidentified_Firmicutes | Oscillospirales | Ruminococcaceae | Ruminococcaceae uncl. | Ruminococcaceae uncl. |
| OTU00527 | Bacteria | Bacteroidota | Bacteroidia | Chitinophagales | Chitinophagaceae | Taibaiella | Taibaiella uncl. |
| OTU00529 | Bacteria | Proteobacteria | Alphaproteobacteria | Rhizobiales | Xanthobacteraceae | Xanthobacteraceae uncl. | Xanthobacteraceae uncl. |
| OTU00530 | Bacteria | Proteobacteria | Alphaproteobacteria | Sphingomonadales | Sphingomonadaceae | Sphingomonadaceae uncl. | Sphingomonadaceae uncl. |
| OTU00531 | Bacteria | Firmicutes | Clostridia | Christensenellales | Christensenellaceae | Christensenellaceae_R-7_group | Christensenellaceae_R-7_group uncl. |
| OTU00532 | Bacteria | Bacteroidota | Bacteroidia | Bacteroidales | Prevotellaceae | Prevotellaceae uncl. | Prevotellaceae uncl. |
| OTU00534 | Bacteria | Proteobacteria | Gammaproteobacteria | Xanthomonadales | Xanthomonadaceae | Stenotrophomonas | Stenotrophomonas_maltophilia |
| OTU00535 | Bacteria | Firmicutes | Clostridia | Lachnospirales | Lachnospiraceae | Coprococcus | Coprococcus_eutactus |
| OTU00536 | Bacteria | Bacteroidota | Bacteroidia | Bacteroidales | Prevotellaceae | Prevotella | Prevotella_sp_S7-1-8 |
| OTU00538 | Bacteria | Verrucomicrobiota | Verrucomicrobiae | Verrucomicrobiales | Rubritaleaceae | Luteolibacter | Verrucomicrobium_sp_IMCC25902 |
| OTU00541 | Bacteria | Firmicutes | Clostridia | Lachnospirales | Lachnospiraceae | Lachnoclostridium | Lachnoclostridium uncl. |
| OTU00542 | Bacteria | Proteobacteria | Alphaproteobacteria | Rhizobiales | Rhizobiaceae | Mesorhizobium | Mesorhizobium uncl. |
| OTU00543 | Bacteria | Bacteroidota | Bacteroidia | Sphingobacteriales | Sphingobacteriaceae | Mucilaginibacter | Mucilaginibacter uncl. |
| OTU00544 | Bacteria | Fusobacteriota | Fusobacteriia | Fusobacteriales | Fusobacteriaceae | Fusobacterium | Fusobacterium uncl. |
| OTU00547 | Bacteria | Proteobacteria | Alphaproteobacteria | Rhizobiales | Beijerinckiaceae | Methylobacterium-Methylorubrum | Methylobacterium-Methylorubrum uncl. |
| OTU00548 | Bacteria | Actinobacteriota | Actinobacteria | Corynebacteriales | Nocardiaceae | Smaragdicoccus | Smaragdicoccus uncl. |
| OTU00549 | Bacteria | unclassified | unclassified | unclassified | unclassified | unclassified | unclassified |
| OTU00550 | Bacteria | Proteobacteria | Alphaproteobacteria | Rhizobiales | Beijerinckiaceae | 1174-901-12 | 1174-901-12 uncl. |
| OTU00552 | Bacteria | Proteobacteria | Alphaproteobacteria | Rhizobiales | Labraceae | Labrys | Labrys uncl. |
| OTU00553 | Bacteria | Proteobacteria | Gammaproteobacteria | Pseudomonadales | Moraxellaceae | Alkanindiges | Alkanindiges uncl. |
| OTU00554 | Bacteria | Firmicutes | Bacilli | Erysipelotrichales | Erysipelotrichaceae | Allobaculum | Allobaculum uncl. |
| OTU00556 | Bacteria | Actinobacteriota | Actinobacteria | Micrococcales | Dermacoccaceae | Dermacoccus | Dermacoccus uncl. |
| OTU00558 | Bacteria | Firmicutes | Clostridia | Oscillospirales | Oscillospiraceae | UCG-005 | UCG-005 uncl. |
| OTU00561 | Bacteria | Proteobacteria | Alphaproteobacteria | Acetobacterales | Acetobacteraceae | Commensalibacter | Commensalibacter uncl. |
| OTU00567 | Bacteria | Firmicutes | Negativicutes | Veillonellales-Selenomonadales | Veillonellaceae | Megasphaera | Megasphaera_stantonii |
| OTU00569 | Bacteria | Firmicutes | Clostridia | Lachnospirales | Lachnospiraceae | Lachnospiraceae_ND3007_group | Lachnospiraceae_ND3007_group uncl. |
| OTU00572 | Bacteria | Proteobacteria | Alphaproteobacteria | Caulobacterales | Caulobacteraceae | Caulobacteraceae uncl. | Caulobacteraceae uncl. |
| OTU00574 | Bacteria | Proteobacteria | Gammaproteobacteria | Enterobacterales | Enterobacterales uncl. | Enterobacterales uncl. | Enterobacterales uncl. |
| OTU00576 | Bacteria | Proteobacteria | Alphaproteobacteria | Rickettsiales | Anaplasmataceae | Wolbachia | Wolbachia uncl. |
| OTU00577 | Bacteria | unclassified | unclassified | unclassified | unclassified | unclassified | unclassified |
| OTU00581 | Bacteria | unclassified | unclassified | unclassified | unclassified | unclassified | unclassified |
| OTU00582 | Bacteria | Proteobacteria | Alphaproteobacteria | Rhodospirillales | unidentified_Rhodospirillales | unidentified_Rhodospirillales | Azospirillum_sp_47_25 |
| OTU00584 | Bacteria | Proteobacteria | Gammaproteobacteria | Pseudomonadales | Cellvibrionaceae | Cellvibrio | Cellvibrio uncl. |
| OTU00585 | Bacteria | unclassified | unclassified | unclassified | unclassified | unclassified | unclassified |
| OTU00587 | Bacteria | Proteobacteria | Gammaproteobacteria | Gammaproteobacteria uncl. | Gammaproteobacteria uncl. | Gammaproteobacteria uncl. | Gammaproteobacteria uncl. |
| OTU00588 | Bacteria | Proteobacteria | Alphaproteobacteria | Caulobacterales | Caulobacteraceae | Caulobacteraceae uncl. | Caulobacteraceae uncl. |
| OTU00589 | Bacteria | Actinobacteriota | Actinobacteria | Propionibacteriales | Nocardioidaceae | Nocardioides | Nocardioides uncl. |
| OTU00590 | Bacteria | Proteobacteria | Alphaproteobacteria | Sphingomonadales | Sphingomonadaceae | Rhizorhapis | bacterium_enrichment_culture_clone_heteroB40_4W |
| OTU00592 | Bacteria | Acidobacteriota | Acidobacteriae | Acidobacteriales | Acidobacteriaceae_(Subgroup_1) | Granulicella | Granulicella uncl. |
| OTU00593 | Bacteria | WPS-2 | WPS-2 uncl. | WPS-2 uncl. | WPS-2 uncl. | WPS-2 uncl. | WPS-2 uncl. |
| OTU00594 | Bacteria | Actinobacteriota | Actinobacteria | Micrococcales | Microbacteriaceae | Leucobacter | Leucobacter uncl. |
| OTU00597 | Bacteria | Bacteroidota | Bacteroidia | Sphingobacteriales | env.OPS_17 | env.OPS_17 uncl. | env.OPS_17 uncl. |
| OTU00600 | Bacteria | Bacteroidota | Bacteroidia | Bacteroidales | Muribaculaceae | Muribaculaceae uncl. | Muribaculaceae uncl. |
| OTU00602 | Bacteria | Armatimonadota | Armatimonadia | Armatimonadales | Armatimonadales uncl. | Armatimonadales uncl. | Armatimonadales uncl. |
| OTU00605 | Bacteria | Proteobacteria | Alphaproteobacteria | Rhizobiales | Rhizobiaceae | Rhizobiaceae uncl. | Rhizobiaceae uncl. |
| OTU00606 | Bacteria | Proteobacteria | Alphaproteobacteria | Sphingomonadales | Sphingomonadaceae | Polymorphobacter | Polymorphobacter uncl. |
| OTU00607 | Bacteria | Proteobacteria | Alphaproteobacteria | Rickettsiales | Anaplasmataceae | Wolbachia | Wolbachia uncl. |
| OTU00610 | Bacteria | Proteobacteria | Gammaproteobacteria | Burkholderiales | Sutterellaceae | Sutterella | Sutterella uncl. |
| OTU00611 | Bacteria | Actinobacteriota | Actinobacteria | Bifidobacteriales | Bifidobacteriaceae | Bifidobacterium | Bifidobacterium uncl. |
| OTU00613 | Bacteria | Firmicutes | Clostridia | Oscillospirales | Oscillospiraceae | UCG-002 | UCG-002 uncl. |
| OTU00617 | Bacteria | Actinobacteriota | Acidimicrobiia | Acidimicrobiia uncl. | Acidimicrobiia uncl. | Acidimicrobiia uncl. | Acidimicrobiia uncl. |
| OTU00623 | Bacteria | unclassified | unclassified | unclassified | unclassified | unclassified | unclassified |
| OTU00625 | Bacteria | Bacteroidota | Bacteroidia | Bacteroidales | Muribaculaceae | Muribaculaceae uncl. | Muribaculaceae uncl. |
| OTU00626 | Bacteria | Firmicutes | Clostridia | Lachnospirales | Lachnospirales uncl. | Lachnospirales uncl. | Lachnospirales uncl. |
| OTU00627 | Bacteria | Verrucomicrobiota | Verrucomicrobiae | Verrucomicrobiales | Akkermansiaceae | Akkermansia | Akkermansia_muciniphila |
| OTU00629 | Bacteria | Firmicutes | Bacilli | Erysipelotrichales | Erysipelotrichaceae | Allobaculum | Allobaculum uncl. |
| OTU00630 | Bacteria | unclassified | unclassified | unclassified | unclassified | unclassified | unclassified |
| OTU00632 | Bacteria | Desulfobacterota | Desulfovibrionia | Desulfovibrionales | Desulfovibrionaceae | Desulfovibrio | Desulfovibrio_piger |
| OTU00636 | Bacteria | unclassified | unclassified | unclassified | unclassified | unclassified | unclassified |
| OTU00637 | Bacteria | Firmicutes | Bacilli | Erysipelotrichales | Erysipelotrichaceae | Solobacterium | Solobacterium uncl. |
| OTU00638 | Bacteria | Actinobacteriota | Actinobacteria | Corynebacteriales | Nocardiaceae | Nocardiaceae uncl. | Nocardiaceae uncl. |
| OTU00639 | Bacteria | Proteobacteria | Gammaproteobacteria | Burkholderiales | Sutterellaceae | Sutterella | Sutterella_wadsworthensis |
| OTU00642 | Bacteria | Bacteroidota | Bacteroidia | Bacteroidales | Bacteroidaceae | Bacteroides | Bacteroides_coprophilus |
| OTU00645 | Bacteria | Verrucomicrobiota | Kiritimatiellae | WCHB1-41 | WCHB1-41 uncl. | WCHB1-41 uncl. | WCHB1-41 uncl. |
| OTU00647 | Bacteria | Acidobacteriota | Acidobacteriae | Acidobacteriales | Acidobacteriaceae_(Subgroup_1) | Granulicella | Granulicella uncl. |
| OTU00648 | Bacteria | Firmicutes | Clostridia | Lachnospirales | Lachnospiraceae | Lachnospiraceae uncl. | Lachnospiraceae uncl. |
| OTU00650 | Bacteria | unclassified | unclassified | unclassified | unclassified | unclassified | unclassified |
| OTU00652 | Bacteria | unclassified | unclassified | unclassified | unclassified | unclassified | unclassified |
| OTU00653 | Bacteria | unclassified | unclassified | unclassified | unclassified | unclassified | unclassified |
| OTU00654 | Bacteria | unclassified | unclassified | unclassified | unclassified | unclassified | unclassified |
| OTU00659 | Bacteria | Firmicutes | Clostridia | Lachnospirales | Lachnospiraceae | [Eubacterium]_hallii_group | [Eubacterium]_hallii_group uncl. |
| OTU00660 | Bacteria | unclassified | unclassified | unclassified | unclassified | unclassified | unclassified |
| OTU00661 | Bacteria | unclassified | unclassified | unclassified | unclassified | unclassified | unclassified |
| OTU00662 | Bacteria | unclassified | unclassified | unclassified | unclassified | unclassified | unclassified |
| OTU00668 | Bacteria | Proteobacteria | Alphaproteobacteria | Rickettsiales | Anaplasmataceae | Wolbachia | Wolbachia uncl. |
| OTU00669 | Bacteria | unclassified | unclassified | unclassified | unclassified | unclassified | unclassified |
| OTU00670 | Bacteria | Actinobacteriota | Thermoleophilia | Solirubrobacterales | Solirubrobacteraceae | Conexibacter | Conexibacter uncl. |
| OTU00671 | Bacteria | unclassified | unclassified | unclassified | unclassified | unclassified | unclassified |
| OTU00672 | Bacteria | Actinobacteriota | Actinobacteria | Micrococcales | Intrasporangiaceae | Intrasporangiaceae uncl. | Intrasporangiaceae uncl. |
| OTU00682 | Bacteria | Firmicutes | Clostridia | Oscillospirales | Oscillospiraceae | Colidextribacter | Anaerostipes_hadrus |
| OTU00684 | Bacteria | Actinobacteriota | Thermoleophilia | Gaiellales | Gaiellales uncl. | Gaiellales uncl. | Gaiellales uncl. |
| OTU00687 | Bacteria | Firmicutes | Clostridia | Peptostreptococcales-Tissierellales | Anaerovoracaceae | Mogibacterium | Mogibacterium uncl. |
| OTU00688 | Bacteria | Proteobacteria | Gammaproteobacteria | Pseudomonadales | Moraxellaceae | Psychrobacter | Psychrobacter_alimentarius |
| OTU00690 | Bacteria | unclassified | unclassified | unclassified | unclassified | unclassified | unclassified |
| OTU00691 | Bacteria | Proteobacteria | Gammaproteobacteria | Burkholderiales | Sutterellaceae | Sutterella | Sutterella uncl. |
| OTU00694 | Bacteria | Verrucomicrobiota | Kiritimatiellae | WCHB1-41 | WCHB1-41 uncl. | WCHB1-41 uncl. | WCHB1-41 uncl. |
| OTU00696 | Bacteria | Proteobacteria | Gammaproteobacteria | Burkholderiales | Neisseriaceae | Snodgrassella | Snodgrassella uncl. |
| OTU00699 | Bacteria | Proteobacteria | Alphaproteobacteria | Rhodobacterales | Rhodobacteraceae | Rubellimicrobium | Rubellimicrobium uncl. |
| OTU00701 | Bacteria | Proteobacteria | Alphaproteobacteria | Rhizobiales | Xanthobacteraceae | Rhodopseudomonas | Rhodopseudomonas uncl. |
| OTU00704 | Bacteria | Bacteroidota | Bacteroidia | Bacteroidales | Rikenellaceae | Alistipes | Alistipes uncl. |
| OTU00705 | Bacteria | Firmicutes | Bacilli | Thermicanales | Thermicanaceae | Thermicanus | Thermicanus uncl. |
| OTU00707 | Bacteria | Firmicutes | Clostridia | Lachnospirales | Lachnospiraceae | [Eubacterium]_ventriosum_group | [Eubacterium]_ventriosum_group uncl. |
| OTU00708 | Bacteria | Firmicutes | Clostridia | Peptostreptococcales-Tissierellales | Peptostreptococcaceae | Romboutsia | Romboutsia_ilealis |
| OTU00709 | Bacteria | unclassified | unclassified | unclassified | unclassified | unclassified | unclassified |
| OTU00710 | Bacteria | Firmicutes | Clostridia | Oscillospirales | Ruminococcaceae | Subdoligranulum | Subdoligranulum uncl. |
| OTU00711 | Bacteria | Firmicutes | Clostridia | Lachnospirales | Lachnospiraceae | Stomatobaculum | Stomatobaculum uncl. |
| OTU00713 | Bacteria | unclassified | unclassified | unclassified | unclassified | unclassified | unclassified |
| OTU00714 | Bacteria | unclassified | unclassified | unclassified | unclassified | unclassified | unclassified |
| OTU00716 | Bacteria | Firmicutes | Clostridia | Oscillospirales | Butyricicoccaceae | UCG-008 | UCG-008 uncl. |
| OTU00719 | Bacteria | unclassified | unclassified | unclassified | unclassified | unclassified | unclassified |
| OTU00720 | Bacteria | Myxococcota | Myxococcia | Myxococcales | Myxococcaceae | Corallococcus | Corallococcus_coralloides |
| OTU00722 | Bacteria | unclassified | unclassified | unclassified | unclassified | unclassified | unclassified |
| OTU00723 | Bacteria | Bacteroidota | Bacteroidia | Bacteroidales | Bacteroidaceae | Bacteroides | Bacteroides uncl. |
| OTU00724 | Bacteria | Bacteroidota | Bacteroidia | Sphingobacteriales | Sphingobacteriaceae | Mucilaginibacter | Mucilaginibacter uncl. |
| OTU00726 | Bacteria | Myxococcota | Polyangia | Haliangiales | Haliangiaceae | Haliangium | Haliangium uncl. |
| OTU00727 | Bacteria | Firmicutes | Clostridia | Lachnospirales | Lachnospiraceae | Oribacterium | Oribacterium uncl. |
| OTU00728 | Bacteria | Patescibacteria | Saccharimonadia | Saccharimonadales | Saccharimonadaceae | TM7a | TM7a uncl. |
| OTU00730 | Bacteria | unclassified | unclassified | unclassified | unclassified | unclassified | unclassified |
| OTU00731 | Bacteria | Bacteroidota | Bacteroidia | Sphingobacteriales | Sphingobacteriales uncl. | Sphingobacteriales uncl. | Sphingobacteriales uncl. |
| OTU00732 | Bacteria | Myxococcota | bacteriap25 | bacteriap25 uncl. | bacteriap25 uncl. | bacteriap25 uncl. | bacteriap25 uncl. |
| OTU00733 | Bacteria | Proteobacteria | Gammaproteobacteria | Enterobacterales | Aeromonadaceae | Aeromonas | Aeromonas uncl. |
| OTU00734 | Bacteria | Bacteroidota | Bacteroidia | Chitinophagales | Chitinophagaceae | Ferruginibacter | Ferruginibacter uncl. |
| OTU00736 | Bacteria | Firmicutes | Clostridia | Oscillospirales | Ruminococcaceae | [Eubacterium]_siraeum_group | [Eubacterium]_siraeum_group uncl. |
| OTU00737 | Bacteria | Chloroflexi | KD4-96 | KD4-96 uncl. | KD4-96 uncl. | KD4-96 uncl. | KD4-96 uncl. |
| OTU00738 | Bacteria | Proteobacteria | Gammaproteobacteria | Xanthomonadales | Rhodanobacteraceae | Dokdonella | Dokdonella uncl. |
| OTU00739 | Bacteria | Firmicutes | Clostridia | Oscillospirales | Butyricicoccaceae | Butyricicoccaceae uncl. | Butyricicoccaceae uncl. |
| OTU00740 | Bacteria | Firmicutes | Clostridia | Oscillospirales | Oscillospiraceae | Flavonifractor | Flavonifractor uncl. |
| OTU00742 | Bacteria | Proteobacteria | Proteobacteria uncl. | Proteobacteria uncl. | Proteobacteria uncl. | Proteobacteria uncl. | Proteobacteria uncl. |
| OTU00743 | Bacteria | Proteobacteria | Gammaproteobacteria | Pseudomonadales | Pseudomonadaceae | Pseudomonas | Pseudomonas uncl. |
| OTU00745 | Bacteria | Actinobacteriota | Coriobacteriia | Coriobacteriia uncl. | Coriobacteriia uncl. | Coriobacteriia uncl. | Coriobacteriia uncl. |
| OTU00747 | Bacteria | unclassified | unclassified | unclassified | unclassified | unclassified | unclassified |
| OTU00748 | Bacteria | Firmicutes | Clostridia | Lachnospirales | Lachnospiraceae | Blautia | Blautia_obeum |
| OTU00749 | Bacteria | unclassified | unclassified | unclassified | unclassified | unclassified | unclassified |
| OTU00750 | Bacteria | Firmicutes | Clostridia | Lachnospirales | Lachnospiraceae | Lachnoclostridium | Lachnoclostridium uncl. |
| OTU00756 | Bacteria | Firmicutes | Bacilli | Bacillales | Bacillaceae | Bacillaceae uncl. | Bacillaceae uncl. |
| OTU00757 | Bacteria | unclassified | unclassified | unclassified | unclassified | unclassified | unclassified |
| OTU00759 | Bacteria | Actinobacteriota | Actinobacteria | Corynebacteriales | Nocardiaceae | Rhodococcus | Rhodococcus_wratislaviensis |
| OTU00760 | Bacteria | Actinobacteriota | Actinobacteria | Streptomycetales | Streptomycetaceae | Streptomyces | Streptomyces uncl. |
| OTU00761 | Bacteria | Proteobacteria | Gammaproteobacteria | Enterobacterales | Enterobacterales uncl. | Enterobacterales uncl. | Enterobacterales uncl. |
| OTU00762 | Bacteria | Proteobacteria | Gammaproteobacteria | Xanthomonadales | Rhodanobacteraceae | Chujaibacter | Chujaibacter uncl. |
| OTU00768 | Bacteria | unclassified | unclassified | unclassified | unclassified | unclassified | unclassified |
| OTU00769 | Bacteria | Firmicutes | Clostridia | Oscillospirales | Ruminococcaceae | Ruminococcaceae uncl. | Ruminococcaceae uncl. |
| OTU00772 | Bacteria | Proteobacteria | Gammaproteobacteria | WD260 | WD260 uncl. | WD260 uncl. | WD260 uncl. |
| OTU00773 | Bacteria | Firmicutes | Bacilli | Bacillales | Bacillaceae | Bacillaceae uncl. | Bacillaceae uncl. |
| OTU00774 | Bacteria | Proteobacteria | Alphaproteobacteria | Rhizobiales | Beijerinckiaceae | Beijerinckiaceae uncl. | Beijerinckiaceae uncl. |
| OTU00778 | Bacteria | Proteobacteria | Alphaproteobacteria | Rhizobiales | Xanthobacteraceae | Xanthobacter | Xanthobacter uncl. |
| OTU00779 | Bacteria | unclassified | unclassified | unclassified | unclassified | unclassified | unclassified |
| OTU00780 | Bacteria | Actinobacteriota | Coriobacteriia | Coriobacteriales | Eggerthellaceae | Eggerthellaceae uncl. | Eggerthellaceae uncl. |
| OTU00782 | Bacteria | Proteobacteria | Gammaproteobacteria | Salinisphaerales | Solimonadaceae | unidentified_Solimonadaceae | Sinobacteraceae_bacterium |
| OTU00785 | Bacteria | Firmicutes | unidentified_Firmicutes | Oscillospirales | Ruminococcaceae | Ruminococcaceae uncl. | Ruminococcaceae uncl. |
| OTU00787 | Bacteria | unclassified | unclassified | unclassified | unclassified | unclassified | unclassified |
| OTU00791 | Bacteria | Firmicutes | Negativicutes | Veillonellales-Selenomonadales | Veillonellaceae | Veillonellaceae uncl. | Veillonellaceae uncl. |
| OTU00794 | Bacteria | Actinobacteriota | Coriobacteriia | Coriobacteriales | Eggerthellaceae | Eggerthellaceae uncl. | Eggerthellaceae uncl. |
| OTU00795 | Bacteria | Firmicutes | Clostridia | Oscillospirales | [Eubacterium]_coprostanoligenes_group | [Eubacterium]_coprostanoligenes_group uncl. | [Eubacterium]_coprostanoligenes_group uncl. |
| OTU00796 | Bacteria | Proteobacteria | Gammaproteobacteria | Pseudomonadales | Alcanivoracaceae1 | Alcanivorax | Alcanivorax uncl. |
| OTU00797 | Bacteria | Bacteroidota | Bacteroidia | Sphingobacteriales | Sphingobacteriaceae | Mucilaginibacter | Mucilaginibacter uncl. |
| OTU00799 | Bacteria | Firmicutes | Bacilli | Lactobacillales | Streptococcaceae | Streptococcus | Streptococcus_anginosus |
| OTU00800 | Bacteria | Firmicutes | Bacilli | Lactobacillales | Lactobacillaceae | Lactobacillus | Lactobacillus uncl. |
| OTU00801 | Bacteria | Proteobacteria | Gammaproteobacteria | Burkholderiales | Sutterellaceae | Sutterella | Sutterella uncl. |
| OTU00802 | Bacteria | Firmicutes | Clostridia | Lachnospirales | Lachnospiraceae | Lachnospira | Lachnospira uncl. |
| OTU00807 | Bacteria | Proteobacteria | Gammaproteobacteria | Enterobacterales | Morganellaceae | Morganella | Morganella_morganii |
| OTU00808 | Bacteria | Bacteroidota | Bacteroidia | Bacteroidia uncl. | Bacteroidia uncl. | Bacteroidia uncl. | Bacteroidia uncl. |
| OTU00809 | Bacteria | Firmicutes | Negativicutes | Acidaminococcales | Acidaminococcaceae | Phascolarctobacterium | Phascolarctobacterium uncl. |
| OTU00812 | Bacteria | Proteobacteria | Alphaproteobacteria | Acetobacterales | Acetobacteraceae | Acetobacteraceae uncl. | Acetobacteraceae uncl. |
| OTU00814 | Bacteria | Firmicutes | Clostridia | Oscillospirales | Oscillospiraceae | UCG-002 | UCG-002 uncl. |
| OTU00816 | Bacteria | unclassified | unclassified | unclassified | unclassified | unclassified | unclassified |
| OTU00817 | Bacteria | Firmicutes | Bacilli | Bacillales | Bacillaceae | Oceanobacillus | Oceanobacillus uncl. |
| OTU00819 | Bacteria | Actinobacteriota | Thermoleophilia | Solirubrobacterales | 67-14 | 67-14 uncl. | 67-14 uncl. |
| OTU00820 | Bacteria | Bacteroidota | Bacteroidia | Bacteroidales | Prevotellaceae | Alloprevotella | Alloprevotella uncl. |
| OTU00823 | Bacteria | Proteobacteria | Gammaproteobacteria | Xanthomonadales | Rhodanobacteraceae | Dyella | Dyella uncl. |
| OTU00825 | Bacteria | Proteobacteria | Gammaproteobacteria | Xanthomonadales | Rhodanobacteraceae | Dokdonella | Dokdonella uncl. |
| OTU00826 | Bacteria | Proteobacteria | Alphaproteobacteria | Acetobacterales | Acetobacteraceae | Acidiphilium | Acidiphilium uncl. |
| OTU00831 | Bacteria | Actinobacteriota | Actinobacteria | Kineosporiales | Kineosporiaceae | Kineococcus | Kineococcus uncl. |
| OTU00833 | Bacteria | Proteobacteria | Gammaproteobacteria | Pseudomonadales | Halomonadaceae | Carnimonas | Carnimonas uncl. |
| OTU00834 | Bacteria | Actinobacteriota | Actinobacteria | Propionibacteriales | Propionibacteriaceae | Propionibacterium | Propionibacterium uncl. |
| OTU00835 | Bacteria | Proteobacteria | Gammaproteobacteria | Burkholderiales | Nitrosomonadaceae | MND1 | MND1 uncl. |
| OTU00836 | Bacteria | Actinobacteriota | Acidimicrobiia | IMCC26256 | unidentified_IMCC26256 | unidentified_IMCC26256 | bacterium_enrichment_culture_clone_auto73_4W |
| OTU00838 | Bacteria | Firmicutes | Clostridia | Peptostreptococcales-Tissierellales | Family_XI | Family_XI uncl. | Family_XI uncl. |
| OTU00840 | Bacteria | Actinobacteriota | Thermoleophilia | Solirubrobacterales | Solirubrobacteraceae | Solirubrobacteraceae uncl. | Solirubrobacteraceae uncl. |
| OTU00845 | Bacteria | unclassified | unclassified | unclassified | unclassified | unclassified | unclassified |
| OTU00846 | Bacteria | Proteobacteria | Alphaproteobacteria | Rickettsiales | Anaplasmataceae | Wolbachia | alpha_proteobacterium_endosymbiont_of_Coelostomidia_montana |
| OTU00847 | Bacteria | Proteobacteria | Gammaproteobacteria | Salinisphaerales | Solimonadaceae | Fontimonas | Fontimonas uncl. |
| OTU00849 | Bacteria | Firmicutes | Clostridia | Lachnospirales | Lachnospiraceae | Lachnoanaerobaculum | Lachnoanaerobaculum uncl. |
| OTU00852 | Bacteria | unclassified | unclassified | unclassified | unclassified | unclassified | unclassified |
| OTU00853 | Bacteria | Proteobacteria | Alphaproteobacteria | Sphingomonadales | Sphingomonadaceae | Sphingomonas | Sphingomonas uncl. |
| OTU00854 | Bacteria | Bacteroidota | Bacteroidia | Sphingobacteriales | Sphingobacteriaceae | Mucilaginibacter | Mucilaginibacter uncl. |
| OTU00857 | Bacteria | Firmicutes | Clostridia | Oscillospirales | Ruminococcaceae | Ruminococcus | Ruminococcus uncl. |
| OTU00858 | Bacteria | Proteobacteria | Alphaproteobacteria | Caulobacterales | Caulobacteraceae | Caulobacter | Caulobacter_fusiformis |
| OTU00860 | Bacteria | Actinobacteriota | Actinobacteria | Pseudonocardiales | Pseudonocardiaceae | Pseudonocardia | Pseudonocardia uncl. |
| OTU00863 | Bacteria | Bacteroidota | Bacteroidia | Cytophagales | Hymenobacteraceae | Hymenobacter | Hymenobacter_sp_PAMC_26628 |
| OTU00865 | Bacteria | Actinobacteriota | Actinobacteria | Micrococcales | Microbacteriaceae | Microbacterium | Microbacterium_oxydans |
| OTU00868 | Bacteria | Proteobacteria | Gammaproteobacteria | Diplorickettsiales | Diplorickettsiaceae | Rickettsiella | Rickettsiella uncl. |
| OTU00870 | Bacteria | Actinobacteriota | Coriobacteriia | Coriobacteriales | Atopobiaceae | Libanicoccus | Libanicoccus uncl. |
| OTU00872 | Bacteria | Bacteroidota | Bacteroidia | Bacteroidia uncl. | Bacteroidia uncl. | Bacteroidia uncl. | Bacteroidia uncl. |
| OTU00874 | Bacteria | unclassified | unclassified | unclassified | unclassified | unclassified | unclassified |
| OTU00876 | Bacteria | Actinobacteriota | Actinobacteria | Corynebacteriales | Corynebacteriaceae | Lawsonella | Lawsonella uncl. |
| OTU00877 | Bacteria | unclassified | unclassified | unclassified | unclassified | unclassified | unclassified |
| OTU00878 | Bacteria | unclassified | unclassified | unclassified | unclassified | unclassified | unclassified |
| OTU00879 | Bacteria | Firmicutes | Bacilli | Lactobacillales | Listeriaceae | Brochothrix | Brochothrix_thermosphacta |
| OTU00881 | Bacteria | Chloroflexi | AD3 | AD3 uncl. | AD3 uncl. | AD3 uncl. | AD3 uncl. |
| OTU00885 | Bacteria | unclassified | unclassified | unclassified | unclassified | unclassified | unclassified |
| OTU00889 | Bacteria | Bdellovibrionota | Bdellovibrionia | Bdellovibrionales | Bdellovibrionaceae | Bdellovibrio | Bdellovibrio_bacteriovorus |
| OTU00890 | Bacteria | Proteobacteria | Gammaproteobacteria | Burkholderiales | Alcaligenaceae | Alcaligenaceae uncl. | Alcaligenaceae uncl. |
| OTU00891 | Bacteria | unclassified | unclassified | unclassified | unclassified | unclassified | unclassified |
| OTU00895 | Bacteria | unclassified | unclassified | unclassified | unclassified | unclassified | unclassified |
| OTU00897 | Bacteria | Firmicutes | Bacilli | Erysipelotrichales | Erysipelatoclostridiaceae | Catenibacterium | Catenibacterium uncl. |
| OTU00902 | Bacteria | unclassified | unclassified | unclassified | unclassified | unclassified | unclassified |
| OTU00904 | Bacteria | Proteobacteria | Alphaproteobacteria | Rhodobacterales | Rhodobacteraceae | Rhodobacteraceae uncl. | Rhodobacteraceae uncl. |
| OTU00906 | Bacteria | unclassified | unclassified | unclassified | unclassified | unclassified | unclassified |
| OTU00910 | Bacteria | Firmicutes | Bacilli | Erysipelotrichales | Erysipelotrichaceae | Faecalibaculum | Faecalibaculum_rodentium |
| OTU00911 | Bacteria | Firmicutes | Bacilli | Lactobacillales | Lactobacillaceae | Ligilactobacillus | Lactobacillus_murinus |
| OTU00914 | Bacteria | Proteobacteria | Gammaproteobacteria | Gammaproteobacteria uncl. | Gammaproteobacteria uncl. | Gammaproteobacteria uncl. | Gammaproteobacteria uncl. |
| OTU00916 | Bacteria | Bacteroidota | Bacteroidia | Bacteroidales | Prevotellaceae | Prevotella | Prevotella_stercorea |
| OTU00919 | Bacteria | Bdellovibrionota | Bdellovibrionia | Bacteriovoracales | Bacteriovoracaceae | Peredibacter | Peredibacter uncl. |
| OTU00927 | Bacteria | unclassified | unclassified | unclassified | unclassified | unclassified | unclassified |
| OTU00929 | Bacteria | Bacteroidota | Bacteroidia | Sphingobacteriales | Sphingobacteriaceae | Mucilaginibacter | Mucilaginibacter uncl. |
| OTU00936 | Bacteria | Proteobacteria | Gammaproteobacteria | Xanthomonadales | Xanthomonadaceae | Luteimonas | Luteimonas uncl. |
| OTU00937 | Bacteria | Actinobacteriota | Thermoleophilia | Solirubrobacterales | Solirubrobacteraceae | Solirubrobacteraceae uncl. | Solirubrobacteraceae uncl. |
| OTU00940 | Bacteria | Firmicutes | Bacilli | Lactobacillales | Lactobacillaceae | Lactobacillus | Lactobacillus_iners |
| OTU00941 | Bacteria | Firmicutes | Bacilli | Lactobacillales | Lactobacillaceae | Lactiplantibacillus | Lactiplantibacillus uncl. |
| OTU00942 | Bacteria | Firmicutes | Bacilli | Entomoplasmatales | Spiroplasmataceae | Spiroplasma | Spiroplasma uncl. |
| OTU00944 | Bacteria | Proteobacteria | Gammaproteobacteria | Salinisphaerales | Solimonadaceae | unidentified_Solimonadaceae | Solimonas_sp_CDMK |
| OTU00945 | Bacteria | Proteobacteria | Alphaproteobacteria | Rhizobiales | Devosiaceae | Devosia | Devosia uncl. |
| OTU00947 | Bacteria | unclassified | unclassified | unclassified | unclassified | unclassified | unclassified |
| OTU00948 | Bacteria | unclassified | unclassified | unclassified | unclassified | unclassified | unclassified |
| OTU00954 | Bacteria | Firmicutes | Bacilli | Bacillales | Bacillaceae | Bacillus | Bacillus_psychrosaccharolyticus |
| OTU00956 | Bacteria | Actinobacteriota | Actinobacteria | Propionibacteriales | Nocardioidaceae | Aeromicrobium | Aeromicrobium uncl. |
| OTU00959 | Bacteria | Bacteroidota | Bacteroidia | Bacteroidales | Muribaculaceae | Muribaculaceae uncl. | Muribaculaceae uncl. |
| OTU00962 | Bacteria | Firmicutes | Clostridia | Clostridia_UCG-014 | unidentified_Clostridia_UCG-014 | unidentified_Clostridia_UCG-014 | Clostridiales_bacterium_oral_taxon_075 |
| OTU00965 | Bacteria | Proteobacteria | Gammaproteobacteria | Burkholderiales | Rhodocyclaceae | Dechloromonas | Dechloromonas uncl. |
| OTU00970 | Bacteria | Firmicutes | Bacilli | Bacillales | Bacillaceae | Bacillus | Bacillus_anthracis |
| OTU00973 | Bacteria | unclassified | unclassified | unclassified | unclassified | unclassified | unclassified |
| OTU00976 | Bacteria | Firmicutes | Clostridia | Lachnospirales | Lachnospiraceae | [Ruminococcus]_gnavus_group | [Ruminococcus]_gnavus_group uncl. |
| OTU00978 | Bacteria | Proteobacteria | Alphaproteobacteria | Rhizobiales | Xanthobacteraceae | Rhodopseudomonas | Rhodopseudomonas uncl. |
| OTU00984 | Bacteria | unclassified | unclassified | unclassified | unclassified | unclassified | unclassified |
| OTU00985 | Bacteria | Firmicutes | Clostridia | Oscillospirales | Oscillospiraceae | Oscillibacter | Oscillibacter uncl. |
| OTU00987 | Bacteria | Proteobacteria | Alphaproteobacteria | Sphingomonadales | Sphingomonadaceae | Sphingomonadaceae uncl. | Sphingomonadaceae uncl. |
| OTU00989 | Bacteria | Bacteroidota | Bacteroidia | Flavobacteriales | Weeksellaceae | Apibacter | Apibacter uncl. |
| OTU00990 | Bacteria | Proteobacteria | Gammaproteobacteria | Steroidobacterales | Steroidobacteraceae | Steroidobacter | Steroidobacter uncl. |
| OTU00992 | Bacteria | Proteobacteria | Alphaproteobacteria | Caulobacterales | Caulobacteraceae | Caulobacteraceae uncl. | Caulobacteraceae uncl. |
| OTU01003 | Bacteria | Bacteroidota | Bacteroidia | Bacteroidales | Prevotellaceae | Prevotella_7 | Prevotella_7 uncl. |
| OTU01005 | Bacteria | Proteobacteria | Gammaproteobacteria | PLTA13 | PLTA13 uncl. | PLTA13 uncl. | PLTA13 uncl. |
| OTU01006 | Bacteria | unclassified | unclassified | unclassified | unclassified | unclassified | unclassified |
| OTU01007 | Bacteria | Firmicutes | Clostridia | Lachnospirales | Lachnospiraceae | Coprococcus | Coprococcus uncl. |
| OTU01008 | Bacteria | unclassified | unclassified | unclassified | unclassified | unclassified | unclassified |
| OTU01011 | Bacteria | Firmicutes | Clostridia | Oscillospirales | Oscillospiraceae | Colidextribacter | Colidextribacter uncl. |
| OTU01014 | Bacteria | unclassified | unclassified | unclassified | unclassified | unclassified | unclassified |
| OTU01016 | Bacteria | Proteobacteria | Alphaproteobacteria | Sphingomonadales | Sphingomonadaceae | Sphingoaurantiacus | Sphingoaurantiacus uncl. |
| OTU01017 | Bacteria | Bacteroidota | Bacteroidia | Flavobacteriales | Crocinitomicaceae | Fluviicola | Fluviicola uncl. |
| OTU01019 | Bacteria | Firmicutes | Bacilli | Lactobacillales | Streptococcaceae | Lactococcus | Lactococcus_raffinolactis |
| OTU01020 | Bacteria | Proteobacteria | Gammaproteobacteria | Xanthomonadales | Xanthomonadaceae | Xanthomonadaceae uncl. | Xanthomonadaceae uncl. |
| OTU01023 | Bacteria | unclassified | unclassified | unclassified | unclassified | unclassified | unclassified |
| OTU01026 | Bacteria | Proteobacteria | Alphaproteobacteria | Rhizobiales | Rhizobiales uncl. | Rhizobiales uncl. | Rhizobiales uncl. |
| OTU01036 | Bacteria | Proteobacteria | Gammaproteobacteria | Enterobacterales | Orbaceae | Gilliamella | Gilliamella uncl. |
| OTU01046 | Bacteria | Proteobacteria | Alphaproteobacteria | Rhodobacterales | Rhodobacteraceae | Rubellimicrobium | Rubellimicrobium uncl. |
| OTU01050 | Bacteria | Campylobacterota | Campylobacteria | Campylobacterales | Campylobacteraceae | Campylobacter | Campylobacter_showae |
| OTU01053 | Bacteria | Proteobacteria | Alphaproteobacteria | Reyranellales | Reyranellaceae | Reyranella | Reyranella uncl. |
| OTU01059 | Bacteria | Proteobacteria | Gammaproteobacteria | Xanthomonadales | Xanthomonadaceae | Luteimonas | Luteimonas uncl. |
| OTU01066 | Bacteria | Proteobacteria | Alphaproteobacteria | Rhodobacterales | Rhodobacteraceae | Rhodobacteraceae uncl. | Rhodobacteraceae uncl. |
| OTU01074 | Bacteria | Myxococcota | Polyangia | Polyangiales | Sandaracinaceae | Sandaracinaceae uncl. | Sandaracinaceae uncl. |
| OTU01075 | Bacteria | Firmicutes | Clostridia | Oscillospirales | Ruminococcaceae | Ruminococcus | Ruminococcus uncl. |
| OTU01079 | Bacteria | unclassified | unclassified | unclassified | unclassified | unclassified | unclassified |
| OTU01080 | Bacteria | Proteobacteria | Gammaproteobacteria | Xanthomonadales | Rhodanobacteraceae | Pseudofulvimonas | Pseudofulvimonas uncl. |
| OTU01082 | Bacteria | Actinobacteriota | Actinobacteria | Frankiales | Nakamurellaceae | Nakamurella | Nakamurella uncl. |
| OTU01087 | Bacteria | unclassified | unclassified | unclassified | unclassified | unclassified | unclassified |
| OTU01088 | Bacteria | unclassified | unclassified | unclassified | unclassified | unclassified | unclassified |
| OTU01090 | Bacteria | unclassified | unclassified | unclassified | unclassified | unclassified | unclassified |
| OTU01094 | Bacteria | unclassified | unclassified | unclassified | unclassified | unclassified | unclassified |
| OTU01097 | Bacteria | Acidobacteriota | Acidobacteriae | Acidobacteriales | Acidobacteriaceae_(Subgroup_1) | Granulicella | Granulicella uncl. |
| OTU01101 | Bacteria | Bacteroidota | Bacteroidia | Flavobacteriales | Flavobacteriaceae | Flavobacteriaceae uncl. | Flavobacteriaceae uncl. |
| OTU01102 | Bacteria | Firmicutes | Bacilli | Lactobacillales | Lactobacillaceae | Ligilactobacillus | Lactobacillus_aviarius |
| OTU01108 | Bacteria | Actinobacteriota | Actinobacteria | Micrococcales | Microbacteriaceae | Amnibacterium | Amnibacterium uncl. |
| OTU01110 | Bacteria | Proteobacteria | Alphaproteobacteria | Rhizobiales | Beijerinckiaceae | Roseiarcus | Roseiarcus uncl. |
| OTU01112 | Bacteria | Acidobacteriota | Vicinamibacteria | Vicinamibacterales | Vicinamibacteraceae | Vicinamibacteraceae uncl. | Vicinamibacteraceae uncl. |
| OTU01115 | Bacteria | Armatimonadota | Armatimonadia | Armatimonadales | Armatimonadaceae | Armatimonas | Armatimonas uncl. |
| OTU01116 | Bacteria | Proteobacteria | Alphaproteobacteria | Sphingomonadales | Sphingomonadaceae | Parablastomonas | Parablastomonas uncl. |
| OTU01117 | Bacteria | unclassified | unclassified | unclassified | unclassified | unclassified | unclassified |
| OTU01119 | Bacteria | unclassified | unclassified | unclassified | unclassified | unclassified | unclassified |
| OTU01124 | Bacteria | unclassified | unclassified | unclassified | unclassified | unclassified | unclassified |
| OTU01125 | Bacteria | Proteobacteria | Gammaproteobacteria | Xanthomonadales | Xanthomonadaceae | Xanthomonadaceae uncl. | Xanthomonadaceae uncl. |
| OTU01128 | Bacteria | Proteobacteria | Alphaproteobacteria | Sphingomonadales | Sphingomonadaceae | Polymorphobacter | Polymorphobacter_sp |
| OTU01129 | Bacteria | Proteobacteria | Alphaproteobacteria | Rhizobiales | Rhizobiaceae | Rhizobiaceae uncl. | Rhizobiaceae uncl. |
| OTU01130 | Bacteria | Actinobacteriota | Thermoleophilia | Gaiellales | Gaiellaceae | Gaiella | Gaiella uncl. |
| OTU01138 | Bacteria | Proteobacteria | Gammaproteobacteria | Pseudomonadales | Pseudomonadaceae | Pseudomonas | Pseudomonas uncl. |
| OTU01141 | Bacteria | Firmicutes | Clostridia | Lachnospirales | Lachnospiraceae | Lachnospiraceae uncl. | Lachnospiraceae uncl. |
| OTU01144 | Bacteria | Firmicutes | Clostridia | Oscillospirales | [Eubacterium]_coprostanoligenes_group | [Eubacterium]_coprostanoligenes_group uncl. | [Eubacterium]_coprostanoligenes_group uncl. |
| OTU01145 | Bacteria | Proteobacteria | Alphaproteobacteria | Acetobacterales | Acetobacteraceae | Roseomonas | Roseomonas uncl. |
| OTU01149 | Bacteria | Proteobacteria | Gammaproteobacteria | Burkholderiales | Burkholderiaceae | Polynucleobacter | Polynucleobacter_asymbioticus |
| OTU01151 | Bacteria | unclassified | unclassified | unclassified | unclassified | unclassified | unclassified |
| OTU01153 | Bacteria | Actinobacteriota | Thermoleophilia | Solirubrobacterales | 67-14 | 67-14 uncl. | 67-14 uncl. |
| OTU01156 | Bacteria | Proteobacteria | Gammaproteobacteria | Burkholderiales | Burkholderiaceae | Burkholderia-Caballeronia-Paraburkholderia | Burkholderia-Caballeronia-Paraburkholderia uncl. |
| OTU01157 | Bacteria | Firmicutes | Bacilli | Paenibacillales | Paenibacillaceae | Paenibacillus | Paenibacillus_hordei |
| OTU01162 | Bacteria | Proteobacteria | Gammaproteobacteria | Xanthomonadales | Xanthomonadaceae | Pseudoxanthomonas | Pseudoxanthomonas uncl. |
| OTU01163 | Bacteria | Proteobacteria | Alphaproteobacteria | Sphingomonadales | Sphingomonadaceae | Sphingomonas | Sphingomonas uncl. |
| OTU01164 | Bacteria | Firmicutes | Bacilli | Bacillales | Bacillaceae | Bacillaceae uncl. | Bacillaceae uncl. |
| OTU01171 | Bacteria | Proteobacteria | Alphaproteobacteria | Sphingomonadales | Sphingomonadaceae | Sphingomonas | Sphingomonas uncl. |
| OTU01173 | Bacteria | Actinobacteriota | Actinobacteria | Micrococcales | Micrococcaceae | Paenarthrobacter | Paenarthrobacter_nitroguajacolicus |
| OTU01176 | Bacteria | Firmicutes | Clostridia | Oscillospirales | Ruminococcaceae | Ruminococcus | Ruminococcus uncl. |
| OTU01180 | Bacteria | Acidobacteriota | Holophagae | Subgroup_7 | Subgroup_7 uncl. | Subgroup_7 uncl. | Subgroup_7 uncl. |
| OTU01182 | Bacteria | unclassified | unclassified | unclassified | unclassified | unclassified | unclassified |
| OTU01184 | Bacteria | Proteobacteria | Alphaproteobacteria | Rhizobiales | Rhizobiaceae | Cohaesibacter | Cohaesibacter uncl. |
| OTU01186 | Bacteria | Proteobacteria | Alphaproteobacteria | Rhodobacterales | Rhodobacteraceae | Roseovarius | Roseovarius uncl. |
| OTU01189 | Bacteria | Proteobacteria | Alphaproteobacteria | Acetobacterales | Acetobacteraceae | Acetobacteraceae uncl. | Acetobacteraceae uncl. |
| OTU01195 | Bacteria | Acidobacteriota | Acidobacteriae | Acidobacteriales | Acidobacteriaceae_(Subgroup_1) | Granulicella | Granulicella uncl. |
| OTU01197 | Bacteria | unclassified | unclassified | unclassified | unclassified | unclassified | unclassified |
| OTU01205 | Bacteria | unclassified | unclassified | unclassified | unclassified | unclassified | unclassified |
| OTU01210 | Bacteria | Proteobacteria | Alphaproteobacteria | Rhizobiales | Devosiaceae | Devosia | Devosia uncl. |
| OTU01211 | Bacteria | Actinobacteriota | Thermoleophilia | Solirubrobacterales | Solirubrobacteraceae | Patulibacter | Patulibacter_minatonensis |
| OTU01215 | Bacteria | Actinobacteriota | Actinobacteria | Frankiales | Geodermatophilaceae | Blastococcus | Blastococcus uncl. |
| OTU01219 | Bacteria | Proteobacteria | Gammaproteobacteria | Salinisphaerales | Solimonadaceae | Nevskia | Nevskia uncl. |
| OTU01220 | Bacteria | Actinobacteriota | Actinobacteria | Corynebacteriales | Corynebacteriaceae | Corynebacterium | Corynebacterium uncl. |
| OTU01221 | Bacteria | unclassified | unclassified | unclassified | unclassified | unclassified | unclassified |
| OTU01226 | Bacteria | Firmicutes | Bacilli | Bacilli uncl. | Bacilli uncl. | Bacilli uncl. | Bacilli uncl. |
| OTU01229 | Bacteria | Proteobacteria | Gammaproteobacteria | Pseudomonadales | Nitrincolaceae | Neptunomonas | Neptunomonas_phycophila |
| OTU01230 | Bacteria | Firmicutes | Bacilli | Bacillales | Bacillaceae | Bacillus | Bacillus_thermoamylovorans |
| OTU01232 | Bacteria | Verrucomicrobiota | Verrucomicrobiae | Verrucomicrobiales | Verrucomicrobiaceae | Prosthecobacter | Prosthecobacter uncl. |
| OTU01235 | Bacteria | Bacteroidota | Bacteroidia | Cytophagales | Hymenobacteraceae | Hymenobacter | Hymenobacter uncl. |
| OTU01239 | Bacteria | Proteobacteria | Alphaproteobacteria | Acetobacterales | Acetobacteraceae | Acetobacteraceae uncl. | Acetobacteraceae uncl. |
| OTU01242 | Bacteria | unclassified | unclassified | unclassified | unclassified | unclassified | unclassified |
| OTU01255 | Bacteria | Gemmatimonadota | Gemmatimonadetes | Gemmatimonadales | Gemmatimonadaceae | Gemmatimonas | Gemmatimonas uncl. |
| OTU01257 | Bacteria | Bacteroidota | Bacteroidia | Flavobacteriales | Weeksellaceae | Moheibacter | Moheibacter uncl. |
| OTU01258 | Bacteria | unclassified | unclassified | unclassified | unclassified | unclassified | unclassified |
| OTU01264 | Bacteria | unclassified | unclassified | unclassified | unclassified | unclassified | unclassified |
| OTU01265 | Bacteria | Bacteroidota | Bacteroidia | Flavobacteriales | Weeksellaceae | Apibacter | Apibacter uncl. |
| OTU01267 | Bacteria | Firmicutes | Bacilli | Lactobacillales | Lactobacillaceae | Lactobacillaceae uncl. | Lactobacillaceae uncl. |
| OTU01270 | Bacteria | Deinococcota | Deinococci | Deinococcales | Deinococcaceae | Deinococcus | Deinococcus uncl. |
| OTU01275 | Bacteria | unclassified | unclassified | unclassified | unclassified | unclassified | unclassified |
| OTU01278 | Bacteria | unclassified | unclassified | unclassified | unclassified | unclassified | unclassified |
| OTU01279 | Bacteria | unclassified | unclassified | unclassified | unclassified | unclassified | unclassified |
| OTU01281 | Bacteria | Proteobacteria | Alphaproteobacteria | Caulobacterales | Caulobacteraceae | Caulobacteraceae uncl. | Caulobacteraceae uncl. |
| OTU01283 | Bacteria | Bacteroidota | Bacteroidia | Sphingobacteriales | Sphingobacteriaceae | Mucilaginibacter | Mucilaginibacter uncl. |
| OTU01285 | Bacteria | Acidobacteriota | Acidobacteriae | Acidobacteriales | Acidobacteriaceae_(Subgroup_1) | Bryocella | Bryocella uncl. |
| OTU01289 | Bacteria | unclassified | unclassified | unclassified | unclassified | unclassified | unclassified |
| OTU01293 | Bacteria | Proteobacteria | Gammaproteobacteria | Enterobacterales | Enterobacteriaceae | Escherichia-Shigella | Escherichia-Shigella uncl. |
| OTU01297 | Bacteria | Proteobacteria | Gammaproteobacteria | Burkholderiales | Comamonadaceae | Comamonadaceae uncl. | Comamonadaceae uncl. |
| OTU01300 | Bacteria | unclassified | unclassified | unclassified | unclassified | unclassified | unclassified |
| OTU01301 | Bacteria | Firmicutes | Clostridia | Lachnospirales | Lachnospiraceae | [Eubacterium]_eligens_group | [Eubacterium]_eligens_group uncl. |
| OTU01313 | Bacteria | Firmicutes | Clostridia | Lachnospirales | Lachnospiraceae | Lachnospiraceae_NK3A20_group | Lachnospiraceae_NK3A20_group uncl. |
| OTU01314 | Bacteria | Proteobacteria | Alphaproteobacteria | Acetobacterales | Acetobacteraceae | Commensalibacter | Commensalibacter uncl. |
| OTU01319 | Bacteria | Firmicutes | Clostridia | Lachnospirales | Lachnospiraceae | Lachnospira | Lachnospira uncl. |
| OTU01320 | Bacteria | Actinobacteriota | Coriobacteriia | Coriobacteriales | Eggerthellaceae | Slackia | Slackia uncl. |
| OTU01322 | Bacteria | Proteobacteria | Gammaproteobacteria | Pseudomonadales | Moraxellaceae | Alkanindiges | Alkanindiges uncl. |
| OTU01324 | Bacteria | unclassified | unclassified | unclassified | unclassified | unclassified | unclassified |
| OTU01333 | Bacteria | Firmicutes | Clostridia | Lachnospirales | Lachnospiraceae | CHKCI001 | CHKCI001 uncl. |
| OTU01349 | Bacteria | unclassified | unclassified | unclassified | unclassified | unclassified | unclassified |
| OTU01350 | Bacteria | Verrucomicrobiota | Verrucomicrobiae | Verrucomicrobiales | Rubritaleaceae | Luteolibacter | Luteolibacter uncl. |
| OTU01351 | Bacteria | Actinobacteriota | Thermoleophilia | Solirubrobacterales | Solirubrobacteraceae | Patulibacter | Patulibacter uncl. |
| OTU01356 | Bacteria | Actinobacteriota | Actinobacteria | Corynebacteriales | Mycobacteriaceae | Mycobacterium | Mycobacterium uncl. |
| OTU01358 | Bacteria | Bacteroidota | Bacteroidia | Sphingobacteriales | Sphingobacteriaceae | Pedobacter | Pedobacter uncl. |
| OTU01367 | Bacteria | Proteobacteria | Gammaproteobacteria | Burkholderiales | Sutterellaceae | Sutterellaceae uncl. | Sutterellaceae uncl. |
| OTU01371 | Bacteria | unclassified | unclassified | unclassified | unclassified | unclassified | unclassified |
| OTU01375 | Bacteria | unclassified | unclassified | unclassified | unclassified | unclassified | unclassified |
| OTU01378 | Bacteria | Firmicutes | Clostridia | Lachnospirales | Lachnospiraceae | [Ruminococcus]_torques_group | [Ruminococcus]_torques_group uncl. |
| OTU01379 | Bacteria | Acidobacteriota | Acidobacteriae | Acidobacteriales | Acidobacteriaceae_(Subgroup_1) | Granulicella | Granulicella_paludicola |
| OTU01381 | Bacteria | Firmicutes | Clostridia | Oscillospirales | Oscillospiraceae | Oscillibacter | Oscillibacter uncl. |
| OTU01382 | Bacteria | Proteobacteria | Gammaproteobacteria | Enterobacterales | Orbaceae | Orbaceae uncl. | Orbaceae uncl. |
| OTU01385 | Bacteria | Acidobacteriota | Acidobacteriae | Acidobacteriales | Acidobacteriales uncl. | Acidobacteriales uncl. | Acidobacteriales uncl. |
| OTU01393 | Bacteria | Proteobacteria | Alphaproteobacteria | Rhizobiales | Rhizobiaceae | Rhizobiaceae uncl. | Rhizobiaceae uncl. |
| OTU01399 | Bacteria | unclassified | unclassified | unclassified | unclassified | unclassified | unclassified |
| OTU01402 | Bacteria | Desulfobacterota | Desulfobacteria | Desulfobacterales | Desulfobacteraceae | Desulfobacter | Desulfobacter uncl. |
| OTU01405 | Bacteria | Firmicutes | Clostridia | Lachnospirales | Lachnospiraceae | Lachnospiraceae_NK4A136_group | Lachnospiraceae_NK4A136_group uncl. |
| OTU01420 | Bacteria | Proteobacteria | Gammaproteobacteria | Pseudomonadales | Pseudomonadaceae | Pseudomonas | Pseudomonas uncl. |
| OTU01422 | Bacteria | Myxococcota | Polyangia | Polyangiales | Polyangiaceae | Pajaroellobacter | Pajaroellobacter uncl. |
| OTU01427 | Bacteria | unclassified | unclassified | unclassified | unclassified | unclassified | unclassified |
| OTU01429 | Bacteria | unclassified | unclassified | unclassified | unclassified | unclassified | unclassified |
| OTU01432 | Bacteria | Firmicutes | Clostridia | Oscillospirales | Oscillospiraceae | UCG-005 | UCG-005 uncl. |
| OTU01433 | Bacteria | unclassified | unclassified | unclassified | unclassified | unclassified | unclassified |
| OTU01434 | Bacteria | Armatimonadota | Armatimonadia | Armatimonadales | Armatimonadales uncl. | Armatimonadales uncl. | Armatimonadales uncl. |
| OTU01436 | Bacteria | Actinobacteriota | Actinobacteria | Frankiales | Geodermatophilaceae | Geodermatophilus | Geodermatophilus uncl. |
| OTU01438 | Bacteria | Deinococcota | Deinococci | Deinococcales | Deinococcaceae | Deinococcus | Deinococcus uncl. |
| OTU01441 | Bacteria | Proteobacteria | Gammaproteobacteria | Burkholderiales | Comamonadaceae | Tepidimonas | Tepidimonas uncl. |
| OTU01444 | Bacteria | unclassified | unclassified | unclassified | unclassified | unclassified | unclassified |
| OTU01445 | Bacteria | Actinobacteriota | Thermoleophilia | Solirubrobacterales | Solirubrobacteraceae | Solirubrobacteraceae uncl. | Solirubrobacteraceae uncl. |
| OTU01451 | Bacteria | unclassified | unclassified | unclassified | unclassified | unclassified | unclassified |
| OTU01462 | Bacteria | unclassified | unclassified | unclassified | unclassified | unclassified | unclassified |
| OTU01466 | Bacteria | Firmicutes | Clostridia | Oscillospirales | [Eubacterium]_coprostanoligenes_group | [Eubacterium]_coprostanoligenes_group uncl. | [Eubacterium]_coprostanoligenes_group uncl. |
| OTU01468 | Bacteria | Bacteroidota | Bacteroidia | Bacteroidales | Prevotellaceae | Prevotellaceae_UCG-003 | Prevotellaceae_UCG-003 uncl. |
| OTU01471 | Bacteria | Actinobacteriota | Coriobacteriia | Coriobacteriales | Eggerthellaceae | Enterorhabdus | Enterorhabdus uncl. |
| OTU01472 | Bacteria | Bacteroidota | Bacteroidia | Flavobacteriales | Flavobacteriaceae | Flavobacterium | Flavobacterium_sp_CHF1-5-1 |
| OTU01479 | Bacteria | Proteobacteria | Gammaproteobacteria | Pseudomonadales | Moraxellaceae | Perlucidibaca | Perlucidibaca uncl. |
| OTU01481 | Bacteria | Patescibacteria | Saccharimonadia | Saccharimonadales | Saccharimonadaceae | TM7a | TM7a uncl. |
| OTU01491 | Bacteria | Proteobacteria | Alphaproteobacteria | Acetobacterales | Acetobacteraceae | Acidiphilium | Acidiphilium uncl. |
| OTU01493 | Bacteria | Verrucomicrobiota | Verrucomicrobiae | Verrucomicrobiae uncl. | Verrucomicrobiae uncl. | Verrucomicrobiae uncl. | Verrucomicrobiae uncl. |
| OTU01496 | Bacteria | unclassified | unclassified | unclassified | unclassified | unclassified | unclassified |
| OTU01517 | Bacteria | unclassified | unclassified | unclassified | unclassified | unclassified | unclassified |
| OTU01519 | Bacteria | Proteobacteria | Alphaproteobacteria | Rhizobiales | Beijerinckiaceae | Methylobacterium-Methylorubrum | Methylorubrum_extorquens |
| OTU01524 | Bacteria | Actinobacteriota | Actinobacteria | Corynebacteriales | Mycobacteriaceae | Mycobacterium | Mycobacterium uncl. |
| OTU01534 | Bacteria | unclassified | unclassified | unclassified | unclassified | unclassified | unclassified |
| OTU01541 | Bacteria | Actinobacteriota | Thermoleophilia | Solirubrobacterales | Solirubrobacteraceae | Solirubrobacter | Solirubrobacter uncl. |
| OTU01554 | Bacteria | Actinobacteriota | Actinobacteria | Pseudonocardiales | Pseudonocardiaceae | Pseudonocardia | Pseudonocardia uncl. |
| OTU01555 | Bacteria | unclassified | unclassified | unclassified | unclassified | unclassified | unclassified |
| OTU01558 | Bacteria | unclassified | unclassified | unclassified | unclassified | unclassified | unclassified |
| OTU01560 | Bacteria | Verrucomicrobiota | Verrucomicrobiae | Opitutales | Opitutaceae | Opitutaceae uncl. | Opitutaceae uncl. |
| OTU01561 | Bacteria | Proteobacteria | Gammaproteobacteria | Pseudomonadales | Cellvibrionaceae | Cellvibrio | Cellvibrio uncl. |
| OTU01562 | Bacteria | Proteobacteria | Alphaproteobacteria | Acetobacterales | Acetobacteraceae | Rhodovastum | Rhodovastum uncl. |
| OTU01563 | Bacteria | Proteobacteria | Alphaproteobacteria | Sphingomonadales | Sphingomonadaceae | Sphingomonadaceae uncl. | Sphingomonadaceae uncl. |
| OTU01569 | Bacteria | Acidobacteriota | Acidobacteriae | Acidobacteriales | unidentified_Acidobacteriales | unidentified_Acidobacteriales | bacterium_Ellin5257 |
| OTU01595 | Bacteria | Firmicutes | Clostridia | Lachnospirales | Lachnospiraceae | Lachnospiraceae uncl. | Lachnospiraceae uncl. |
| OTU01617 | Bacteria | Firmicutes | Bacilli | Lactobacillales | Lactobacillaceae | Fructobacillus | Fructobacillus_tropaeoli |
| OTU01618 | Bacteria | Firmicutes | Bacilli | Entomoplasmatales | Spiroplasmataceae | Spiroplasma | Spiroplasma_citri |
| OTU01626 | Bacteria | unclassified | unclassified | unclassified | unclassified | unclassified | unclassified |
| OTU01651 | Bacteria | Firmicutes | Clostridia | Lachnospirales | Lachnospiraceae | Blautia | Blautia uncl. |
| OTU01694 | Bacteria | Actinobacteriota | Actinobacteria | Micrococcales | Microbacteriaceae | Microbacteriaceae uncl. | Microbacteriaceae uncl. |
| OTU01702 | Bacteria | Proteobacteria | Gammaproteobacteria | Enterobacterales | Enterobacterales uncl. | Enterobacterales uncl. | Enterobacterales uncl. |
| OTU01720 | Bacteria | unclassified | unclassified | unclassified | unclassified | unclassified | unclassified |
| OTU01734 | Bacteria | Actinobacteriota | Actinobacteria | Micrococcales | Dermabacteraceae | Brachybacterium | Brachybacterium uncl. |
| OTU01737 | Bacteria | unclassified | unclassified | unclassified | unclassified | unclassified | unclassified |
| OTU01739 | Bacteria | Actinobacteriota | Thermoleophilia | Solirubrobacterales | Solirubrobacteraceae | Solirubrobacteraceae uncl. | Solirubrobacteraceae uncl. |
| OTU01740 | Bacteria | unclassified | unclassified | unclassified | unclassified | unclassified | unclassified |
| OTU01756 | Bacteria | Bacteroidota | Bacteroidia | Flavobacteriales | Flavobacteriaceae | Flavobacterium | Flavobacterium uncl. |
| OTU01769 | Bacteria | Proteobacteria | Gammaproteobacteria | Pseudomonadales | Moraxellaceae | Acinetobacter | Acinetobacter uncl. |
| OTU01776 | Bacteria | Proteobacteria | Gammaproteobacteria | Enterobacterales | Enterobacterales uncl. | Enterobacterales uncl. | Enterobacterales uncl. |
| OTU01779 | Bacteria | Bacteroidota | Bacteroidia | Cytophagales | Spirosomaceae | Dyadobacter | Dyadobacter uncl. |
| OTU01786 | Bacteria | Actinobacteriota | Actinobacteria | Frankiales | Sporichthyaceae | Sporichthya | Sporichthya uncl. |
| OTU01792 | Bacteria | Proteobacteria | Alphaproteobacteria | Acetobacterales | Acetobacteraceae | Acidiphilium | Acidiphilium uncl. |
| OTU01800 | Bacteria | unclassified | unclassified | unclassified | unclassified | unclassified | unclassified |
| OTU01820 | Bacteria | Proteobacteria | Gammaproteobacteria | Salinisphaerales | Solimonadaceae | Nevskia | Nevskia uncl. |
| OTU01829 | Bacteria | Actinobacteriota | Actinobacteria | Streptomycetales | Streptomycetaceae | Streptomyces | Streptomyces uncl. |
| OTU01836 | Bacteria | Proteobacteria | Gammaproteobacteria | Xanthomonadales | Rhodanobacteraceae | Rhodanobacter | Rhodanobacter_sp |
| OTU01839 | Bacteria | Proteobacteria | Alphaproteobacteria | Rhizobiales | Beijerinckiaceae | Beijerinckiaceae uncl. | Beijerinckiaceae uncl. |
| OTU01849 | Bacteria | Actinobacteriota | Actinobacteria | Pseudonocardiales | Pseudonocardiaceae | Pseudonocardiaceae uncl. | Pseudonocardiaceae uncl. |
| OTU01858 | Bacteria | unclassified | unclassified | unclassified | unclassified | unclassified | unclassified |
| OTU01874 | Bacteria | Proteobacteria | Gammaproteobacteria | Enterobacterales | Morganellaceae | Providencia | Providencia_heimbachae |
| OTU01887 | Bacteria | Firmicutes | Clostridia | Peptostreptococcales-Tissierellales | Peptostreptococcaceae | Peptostreptococcaceae uncl. | Peptostreptococcaceae uncl. |
| OTU01892 | Bacteria | Actinobacteriota | Actinobacteria | Micrococcales | Cellulomonadaceae | Cellulomonas | Cellulomonas uncl. |
| OTU01912 | Bacteria | unclassified | unclassified | unclassified | unclassified | unclassified | unclassified |
| OTU01919 | Bacteria | Proteobacteria | Gammaproteobacteria | Pseudomonadales | Pseudomonadaceae | Pseudomonas | Pseudomonas_sp_R2A2 |
| OTU01934 | Bacteria | Proteobacteria | Gammaproteobacteria | Enterobacterales | Orbaceae | Gilliamella | Gilliamella uncl. |
| OTU01957 | Bacteria | Proteobacteria | Gammaproteobacteria | Burkholderiales | Comamonadaceae | Comamonadaceae uncl. | Comamonadaceae uncl. |
| OTU01968 | Bacteria | Proteobacteria | Gammaproteobacteria | Xanthomonadales | Xanthomonadaceae | Arenimonas | Arenimonas uncl. |
| OTU01972 | Bacteria | Actinobacteriota | Thermoleophilia | Solirubrobacterales | Solirubrobacteraceae | Conexibacter | Solirubrobacterales_bacterium |
| OTU01992 | Bacteria | Actinobacteriota | Actinobacteria | Frankiales | Acidothermaceae | Acidothermus | Acidothermus uncl. |
| OTU01993 | Bacteria | unclassified | unclassified | unclassified | unclassified | unclassified | unclassified |
| OTU01994 | Bacteria | Proteobacteria | Gammaproteobacteria | Burkholderiales | Neisseriaceae | Snodgrassella | Snodgrassella uncl. |
| OTU01995 | Bacteria | Proteobacteria | Alphaproteobacteria | Sphingomonadales | Sphingomonadaceae | Rhizorhapis | Sphingomonas_sp_YZ-8 |
| OTU02028 | Bacteria | Firmicutes | Clostridia | Christensenellales | Christensenellaceae | Christensenellaceae_R-7_group | Christensenellaceae_R-7_group uncl. |
| OTU02041 | Bacteria | Bacteroidota | Bacteroidia | Flavobacteriales | Weeksellaceae | Chryseobacterium | Chryseobacterium uncl. |
| OTU02071 | Bacteria | Firmicutes | Bacilli | Lactobacillales | Catellicoccaceae | Catellicoccus | Catellicoccus uncl. |
| OTU02088 | Bacteria | unclassified | unclassified | unclassified | unclassified | unclassified | unclassified |
| OTU02110 | Bacteria | unclassified | unclassified | unclassified | unclassified | unclassified | unclassified |
| OTU02147 | Bacteria | Actinobacteriota | Actinobacteria | Micrococcales | Microbacteriaceae | Microbacteriaceae uncl. | Microbacteriaceae uncl. |
| OTU02200 | Bacteria | unclassified | unclassified | unclassified | unclassified | unclassified | unclassified |
| OTU02218 | Bacteria | unclassified | unclassified | unclassified | unclassified | unclassified | unclassified |
| OTU02229 | Bacteria | Acidobacteriota | Acidobacteriae | Acidobacteriales | Acidobacteriaceae_(Subgroup_1) | Granulicella | Granulicella uncl. |
| OTU02243 | Bacteria | Proteobacteria | Alphaproteobacteria | Rhizobiales | Rhizobiaceae | Mesorhizobium | Mesorhizobium uncl. |
| OTU02255 | Bacteria | Firmicutes | Clostridia | Oscillospirales | Oscillospiraceae | Oscillospiraceae uncl. | Oscillospiraceae uncl. |
| OTU02270 | Bacteria | Proteobacteria | Gammaproteobacteria | Enterobacterales | Orbaceae | Orbaceae uncl. | Orbaceae uncl. |
| OTU02289 | Bacteria | Firmicutes | Bacilli | Lactobacillales | Lactobacillales uncl. | Lactobacillales uncl. | Lactobacillales uncl. |
| OTU02306 | Bacteria | unclassified | unclassified | unclassified | unclassified | unclassified | unclassified |
| OTU02317 | Bacteria | Proteobacteria | Gammaproteobacteria | Burkholderiales | Comamonadaceae | Comamonadaceae uncl. | Comamonadaceae uncl. |
| OTU02331 | Bacteria | Proteobacteria | Gammaproteobacteria | Enterobacterales | Enterobacterales uncl. | Enterobacterales uncl. | Enterobacterales uncl. |
| OTU02337 | Bacteria | Actinobacteriota | Actinobacteria | Corynebacteriales | Corynebacteriaceae | Corynebacterium | Corynebacterium uncl. |
| OTU02343 | Bacteria | Firmicutes | Bacilli | Bacillales | Planococcaceae | Planococcaceae uncl. | Planococcaceae uncl. |
| OTU02416 | Bacteria | Proteobacteria | Alphaproteobacteria | Sphingomonadales | Sphingomonadaceae | Sphingomonas | Sphingomonas_astaxanthinifaciens |
| OTU02428 | Bacteria | Proteobacteria | Alphaproteobacteria | Acetobacterales | Acetobacteraceae | Acetobacteraceae uncl. | Acetobacteraceae uncl. |
| OTU02453 | Bacteria | unclassified | unclassified | unclassified | unclassified | unclassified | unclassified |
| OTU02457 | Bacteria | Bacteroidota | Bacteroidia | Sphingobacteriales | Sphingobacteriaceae | Mucilaginibacter | Mucilaginibacter uncl. |
| OTU02465 | Bacteria | Firmicutes | Bacilli | Thermoactinomycetales | Thermoactinomycetaceae | Kroppenstedtia | Kroppenstedtia uncl. |
| OTU02466 | Bacteria | Proteobacteria | Gammaproteobacteria | Enterobacterales | Orbaceae | Gilliamella | Gilliamella uncl. |
| OTU02488 | Bacteria | Firmicutes | Bacilli | Entomoplasmatales | Spiroplasmataceae | Spiroplasma | Spiroplasma_endosymbiont_of_Curculio_elephas |
| OTU02508 | Bacteria | Proteobacteria | Gammaproteobacteria | Burkholderiales | Neisseriaceae | Snodgrassella | Snodgrassella uncl. |
| OTU02546 | Bacteria | Proteobacteria | Gammaproteobacteria | Burkholderiales | Neisseriaceae | Snodgrassella | Snodgrassella uncl. |
| OTU02556 | Bacteria | Firmicutes | Bacilli | Lactobacillales | Lactobacillaceae | Fructobacillus | Fructobacillus_fructosus |
| OTU02572 | Bacteria | Proteobacteria | Gammaproteobacteria | Enterobacterales | Orbaceae | Gilliamella | Gilliamella_apicola |
| OTU02586 | Bacteria | Proteobacteria | Gammaproteobacteria | Burkholderiales | Comamonadaceae | Comamonadaceae uncl. | Comamonadaceae uncl. |
| OTU02597 | Bacteria | Bacteroidota | Bacteroidia | Flavobacteriales | Weeksellaceae | Apibacter | Apibacter uncl. |
| OTU02623 | Bacteria | Proteobacteria | Gammaproteobacteria | Burkholderiales | Neisseriaceae | Neisseriaceae uncl. | Neisseriaceae uncl. |
| OTU02625 | Bacteria | Actinobacteriota | Actinobacteria | Bifidobacteriales | Bifidobacteriaceae | Bombiscardovia | Bombiscardovia uncl. |
| OTU02658 | Bacteria | Bacteroidota | Bacteroidia | Flavobacteriales | Weeksellaceae | Chryseobacterium | Chryseobacterium uncl. |
| OTU02703 | Bacteria | unclassified | unclassified | unclassified | unclassified | unclassified | unclassified |
| OTU02755 | Bacteria | Acidobacteriota | Acidobacteriae | Acidobacteriales | Acidobacteriaceae_(Subgroup_1) | Acidobacteriaceae_(Subgroup_1) uncl. | Acidobacteriaceae_(Subgroup_1) uncl. |
| OTU02769 | Bacteria | Proteobacteria | Gammaproteobacteria | Enterobacterales | Enterobacteriaceae | Enterobacteriaceae uncl. | Enterobacteriaceae uncl. |
| OTU02849 | Bacteria | Actinobacteriota | Actinobacteria | Bifidobacteriales | Bifidobacteriaceae | Bifidobacterium | Bifidobacterium uncl. |
| OTU02907 | Bacteria | unclassified | unclassified | unclassified | unclassified | unclassified | unclassified |
| OTU02909 | Bacteria | Proteobacteria | Gammaproteobacteria | Burkholderiales | Neisseriaceae | Snodgrassella | Snodgrassella uncl. |
| OTU02915 | Bacteria | Actinobacteriota | Actinobacteria | Frankiales | Frankiales uncl. | Frankiales uncl. | Frankiales uncl. |
| OTU02917 | Bacteria | Bacteroidota | Bacteroidia | Sphingobacteriales | Sphingobacteriaceae | Pedobacter | Pedobacter uncl. |
| OTU02928 | Bacteria | Bacteroidota | Bacteroidia | Cytophagales | Hymenobacteraceae | Hymenobacter | Hymenobacter uncl. |
| OTU02962 | Bacteria | Actinobacteriota | Actinobacteria | Propionibacteriales | Nocardioidaceae | Nocardioides | Nocardioides_simplex |
| OTU02981 | Bacteria | unclassified | unclassified | unclassified | unclassified | unclassified | unclassified |
| OTU03049 | Bacteria | Proteobacteria | Alphaproteobacteria | Rhodobacterales | Rhodobacteraceae | Paracoccus | Paracoccus uncl. |
| OTU03050 | Bacteria | Proteobacteria | Gammaproteobacteria | Burkholderiales | Neisseriaceae | Snodgrassella | Snodgrassella uncl. |
| OTU03077 | Bacteria | Proteobacteria | Alphaproteobacteria | Rhizobiales | Beijerinckiaceae | Microvirga | Microvirga uncl. |
| OTU03194 | Bacteria | unclassified | unclassified | unclassified | unclassified | unclassified | unclassified |
| OTU03209 | Bacteria | Proteobacteria | Gammaproteobacteria | Enterobacterales | Enterobacterales uncl. | Enterobacterales uncl. | Enterobacterales uncl. |
| OTU03220 | Bacteria | Bacteroidota | Bacteroidia | Cytophagales | Hymenobacteraceae | Hymenobacter | Hymenobacter uncl. |
| OTU03221 | Bacteria | Proteobacteria | Alphaproteobacteria | Sphingomonadales | Sphingomonadaceae | Sphingomonadaceae uncl. | Sphingomonadaceae uncl. |
| OTU03223 | Bacteria | Proteobacteria | Gammaproteobacteria | Burkholderiales | Neisseriaceae | Snodgrassella | Snodgrassella uncl. |
| OTU03250 | Bacteria | Proteobacteria | Gammaproteobacteria | Enterobacterales | Pectobacteriaceae | Sodalis | Sodalis uncl. |
| OTU03263 | Bacteria | Actinobacteriota | Actinobacteria | Pseudonocardiales | Pseudonocardiaceae | Pseudonocardia | Pseudonocardia uncl. |
| OTU03268 | Bacteria | Bacteroidota | Bacteroidia | Flavobacteriales | Flavobacteriaceae | Flavobacterium | Flavobacterium uncl. |
| OTU03286 | Bacteria | Proteobacteria | Gammaproteobacteria | Pseudomonadales | Moraxellaceae | Acinetobacter | Acinetobacter uncl. |
| OTU03287 | Bacteria | unclassified | unclassified | unclassified | unclassified | unclassified | unclassified |
| OTU03288 | Bacteria | Proteobacteria | Gammaproteobacteria | Salinisphaerales | Solimonadaceae | Alkanibacter | Alkanibacter uncl. |
| OTU03298 | Bacteria | Proteobacteria | Gammaproteobacteria | Pseudomonadales | Moraxellaceae | Acinetobacter | Acinetobacter uncl. |
| OTU03373 | Bacteria | unclassified | unclassified | unclassified | unclassified | unclassified | unclassified |
| OTU03383 | Bacteria | Firmicutes | Bacilli | Lactobacillales | Lactobacillaceae | Lactobacillaceae uncl. | Lactobacillaceae uncl. |
| OTU03440 | Bacteria | Proteobacteria | Gammaproteobacteria | Burkholderiales | Neisseriaceae | Snodgrassella | Snodgrassella uncl. |
| OTU03498 | Bacteria | Proteobacteria | Gammaproteobacteria | Pseudomonadales | Moraxellaceae | Acinetobacter | Acinetobacter uncl. |
| OTU03521 | Bacteria | Proteobacteria | Gammaproteobacteria | Pseudomonadales | Pseudomonadaceae | Pseudomonas | Pseudomonas uncl. |
| OTU03535 | Bacteria | Proteobacteria | Gammaproteobacteria | Enterobacterales | Orbaceae | Orbaceae uncl. | Orbaceae uncl. |
| OTU03544 | Bacteria | Firmicutes | Bacilli | Lactobacillales | Lactobacillaceae | Latilactobacillus | Latilactobacillus uncl. |
| OTU03564 | Bacteria | Actinobacteriota | Coriobacteriia | Coriobacteriales | Coriobacteriaceae | Collinsella | Collinsella uncl. |
| OTU03600 | Bacteria | Firmicutes | Bacilli | Lactobacillales | Streptococcaceae | Streptococcus | Streptococcus uncl. |
| OTU03620 | Bacteria | Bacteroidota | Bacteroidia | Flavobacteriales | Flavobacteriaceae | Flavobacterium | Flavobacterium uncl. |
| OTU03672 | Bacteria | Proteobacteria | Alphaproteobacteria | Sphingomonadales | Sphingomonadaceae | Sphingomonas | Sphingomonas_glacialis |
| OTU03681 | Bacteria | Firmicutes | Clostridia | Lachnospirales | Lachnospiraceae | Lachnospiraceae uncl. | Lachnospiraceae uncl. |
| OTU03697 | Bacteria | Actinobacteriota | Actinobacteria | Streptomycetales | Streptomycetaceae | Streptomyces | Streptomyces uncl. |
| OTU03704 | Bacteria | Actinobacteriota | Actinobacteria | Micrococcales | Microbacteriaceae | Microbacteriaceae uncl. | Microbacteriaceae uncl. |
| OTU03730 | Bacteria | Proteobacteria | Gammaproteobacteria | Enterobacterales | Enterobacterales uncl. | Enterobacterales uncl. | Enterobacterales uncl. |
| OTU03743 | Bacteria | Actinobacteriota | Actinobacteria | Corynebacteriales | Mycobacteriaceae | Mycobacterium | Mycobacterium uncl. |
| OTU03777 | Bacteria | Proteobacteria | Gammaproteobacteria | Pseudomonadales | Moraxellaceae | Moraxellaceae uncl. | Moraxellaceae uncl. |
| OTU03779 | Bacteria | Proteobacteria | Alphaproteobacteria | Rickettsiales | Anaplasmataceae | Wolbachia | alpha_proteobacterium_endosymbiont_of_Coelostomidia_montana |
| OTU03808 | Bacteria | Proteobacteria | Alphaproteobacteria | Acetobacterales | Acetobacteraceae | Saccharibacter | Saccharibacter uncl. |
| OTU03811 | Bacteria | Proteobacteria | Gammaproteobacteria | Enterobacterales | Orbaceae | Gilliamella | Gilliamella uncl. |
| OTU03823 | Bacteria | unclassified | unclassified | unclassified | unclassified | unclassified | unclassified |
| OTU03838 | Bacteria | Actinobacteriota | Actinobacteria | Frankiales | Frankiaceae | Jatrophihabitans | Jatrophihabitans uncl. |
| OTU03852 | Bacteria | Firmicutes | Bacilli | Lactobacillales | Lactobacillaceae | Bombilactobacillus | Bombilactobacillus uncl. |
| OTU03855 | Bacteria | Proteobacteria | Alphaproteobacteria | Rickettsiales | Anaplasmataceae | Wolbachia | Wolbachia uncl. |
| OTU03860 | Bacteria | unclassified | unclassified | unclassified | unclassified | unclassified | unclassified |
| OTU03864 | Bacteria | Actinobacteriota | Actinobacteria | Corynebacteriales | Nocardiaceae | Rhodococcus | Rhodococcus uncl. |
| OTU03866 | Bacteria | Proteobacteria | Alphaproteobacteria | Acetobacterales | Acetobacteraceae | Commensalibacter | Commensalibacter uncl. |
| OTU03896 | Bacteria | unclassified | unclassified | unclassified | unclassified | unclassified | unclassified |
| OTU03909 | Bacteria | Proteobacteria | Gammaproteobacteria | Burkholderiales | Neisseriaceae | Snodgrassella | Snodgrassella_alvi |
| OTU03917 | Bacteria | Proteobacteria | Alphaproteobacteria | Acetobacterales | Acetobacteraceae | Commensalibacter | Commensalibacter uncl. |
| OTU03933 | Bacteria | Proteobacteria | Gammaproteobacteria | Burkholderiales | Neisseriaceae | Snodgrassella | Snodgrassella uncl. |
| OTU03949 | Bacteria | Proteobacteria | Gammaproteobacteria | Enterobacterales | Enterobacterales uncl. | Enterobacterales uncl. | Enterobacterales uncl. |
| OTU03959 | Bacteria | Proteobacteria | Gammaproteobacteria | Enterobacterales | Enterobacteriaceae | Enterobacteriaceae uncl. | Enterobacteriaceae uncl. |
| OTU03960 | Bacteria | Proteobacteria | Gammaproteobacteria | Burkholderiales | Comamonadaceae | Comamonadaceae uncl. | Comamonadaceae uncl. |
| OTU03978 | Bacteria | Actinobacteriota | Actinobacteria | Propionibacteriales | Nocardioidaceae | Nocardioides | Nocardioides uncl. |
| OTU03983 | Bacteria | unclassified | unclassified | unclassified | unclassified | unclassified | unclassified |
| OTU04017 | Bacteria | Proteobacteria | Gammaproteobacteria | Burkholderiales | Neisseriaceae | Snodgrassella | Snodgrassella uncl. |
| OTU04035 | Bacteria | Actinobacteriota | Actinobacteria | Bifidobacteriales | Bifidobacteriaceae | Bifidobacteriaceae uncl. | Bifidobacteriaceae uncl. |
| OTU04052 | Bacteria | Proteobacteria | Alphaproteobacteria | Rhizobiales | Rhizobiales uncl. | Rhizobiales uncl. | Rhizobiales uncl. |
| OTU04090 | Bacteria | Proteobacteria | Alphaproteobacteria | Rhizobiales | Beijerinckiaceae | 1174-901-12 | 1174-901-12 uncl. |
| OTU04093 | Bacteria | Proteobacteria | Gammaproteobacteria | Enterobacterales | Orbaceae | Orbaceae uncl. | Orbaceae uncl. |
| OTU04147 | Bacteria | Proteobacteria | Alphaproteobacteria | Rhizobiales | Beijerinckiaceae | 1174-901-12 | 1174-901-12 uncl. |
| OTU04154 | Bacteria | Proteobacteria | Alphaproteobacteria | Rhizobiales | Devosiaceae | Devosia | Devosia uncl. |
| OTU04161 | Bacteria | unclassified | unclassified | unclassified | unclassified | unclassified | unclassified |
| OTU04174 | Bacteria | Firmicutes | Bacilli | Entomoplasmatales | Spiroplasmataceae | Spiroplasma | Spiroplasma_sp_MF1006 |
| OTU04215 | Bacteria | Proteobacteria | Gammaproteobacteria | Pseudomonadales | Pseudomonadaceae | Pseudomonas | Pseudomonas_sp_DY-1 |
| OTU04235 | Bacteria | Proteobacteria | Gammaproteobacteria | Burkholderiales | Neisseriaceae | Snodgrassella | Snodgrassella uncl. |
| OTU04240 | Bacteria | Bacteroidota | Bacteroidia | Sphingobacteriales | Sphingobacteriaceae | Sphingobacterium | Sphingobacterium uncl. |
| OTU04257 | Bacteria | Proteobacteria | Gammaproteobacteria | Burkholderiales | Neisseriaceae | Snodgrassella | Snodgrassella uncl. |
| OTU04284 | Bacteria | Proteobacteria | Gammaproteobacteria | Enterobacterales | Enterobacterales uncl. | Enterobacterales uncl. | Enterobacterales uncl. |
| OTU04358 | Bacteria | Bacteroidota | Bacteroidia | Flavobacteriales | Weeksellaceae | Chryseobacterium | Chryseobacterium_sp_T16E-39 |
| OTU04409 | Bacteria | Proteobacteria | Gammaproteobacteria | Pseudomonadales | Moraxellaceae | Acinetobacter | Acinetobacter uncl. |
| OTU04417 | Bacteria | Actinobacteriota | Actinobacteria | Micrococcales | Microbacteriaceae | Plantibacter | Plantibacter_flavus |
| OTU04430 | Bacteria | Proteobacteria | Alphaproteobacteria | Rhizobiales | Amb-16S-1323 | Amb-16S-1323 uncl. | Amb-16S-1323 uncl. |
| OTU04433 | Bacteria | Proteobacteria | Gammaproteobacteria | Burkholderiales | Comamonadaceae | Comamonas | Comamonas uncl. |
| OTU04448 | Bacteria | Proteobacteria | Gammaproteobacteria | Burkholderiales | Comamonadaceae | Comamonadaceae uncl. | Comamonadaceae uncl. |
| OTU04461 | Bacteria | Proteobacteria | Gammaproteobacteria | Burkholderiales | Neisseriaceae | Snodgrassella | Snodgrassella_alvi |
| OTU04482 | Bacteria | Proteobacteria | Alphaproteobacteria | Acetobacterales | Acetobacteraceae | Acetobacteraceae uncl. | Acetobacteraceae uncl. |
| OTU04498 | Bacteria | Actinobacteriota | Actinobacteria | Corynebacteriales | Corynebacteriales uncl. | Corynebacteriales uncl. | Corynebacteriales uncl. |
| OTU04502 | Bacteria | Proteobacteria | Gammaproteobacteria | Burkholderiales | Comamonadaceae | Comamonadaceae uncl. | Comamonadaceae uncl. |
| OTU04521 | Bacteria | Bacteroidota | Bacteroidia | Sphingobacteriales | Sphingobacteriaceae | Sphingobacteriaceae uncl. | Sphingobacteriaceae uncl. |
| OTU04530 | Bacteria | Actinobacteriota | Actinobacteria | Corynebacteriales | Nocardiaceae | Nocardia | Nocardia uncl. |
| OTU04538 | Bacteria | Proteobacteria | Alphaproteobacteria | Acetobacterales | Acetobacteraceae | Acetobacteraceae uncl. | Acetobacteraceae uncl. |
| OTU04558 | Bacteria | Proteobacteria | Alphaproteobacteria | Acetobacterales | Acetobacteraceae | Commensalibacter | Commensalibacter uncl. |
| OTU04600 | Bacteria | Proteobacteria | Gammaproteobacteria | Enterobacterales | Erwiniaceae | Erwiniaceae uncl. | Erwiniaceae uncl. |
| OTU04603 | Bacteria | Proteobacteria | Alphaproteobacteria | Rhizobiales | Rhizobiaceae | Rhizobiaceae uncl. | Rhizobiaceae uncl. |
| OTU04647 | Bacteria | Actinobacteriota | Actinobacteria | Corynebacteriales | Dietziaceae | Dietzia | Dietzia_timorensis |
| OTU04648 | Bacteria | Proteobacteria | Alphaproteobacteria | Acetobacterales | Acetobacteraceae | Commensalibacter | Commensalibacter uncl. |
| OTU04711 | Bacteria | Proteobacteria | Gammaproteobacteria | Enterobacterales | Orbaceae | Orbaceae uncl. | Orbaceae uncl. |
| OTU04719 | Bacteria | Actinobacteriota | Actinobacteria | Corynebacteriales | Nocardiaceae | Williamsia | Williamsia uncl. |
| OTU04731 | Bacteria | Proteobacteria | Gammaproteobacteria | Pseudomonadales | Moraxellaceae | Acinetobacter | Acinetobacter uncl. |
| OTU04797 | Bacteria | Bacteroidota | Bacteroidia | Cytophagales | Hymenobacteraceae | Hymenobacter | Hymenobacter uncl. |
| OTU04798 | Bacteria | Proteobacteria | Alphaproteobacteria | Acetobacterales | Acetobacteraceae | Acetobacteraceae uncl. | Acetobacteraceae uncl. |
| OTU04814 | Bacteria | unclassified | unclassified | unclassified | unclassified | unclassified | unclassified |
| OTU04835 | Bacteria | Proteobacteria | Gammaproteobacteria | Enterobacterales | Enterobacterales uncl. | Enterobacterales uncl. | Enterobacterales uncl. |
| OTU04840 | Bacteria | Bacteroidota | Bacteroidia | Sphingobacteriales | Sphingobacteriaceae | Pedobacter | Pedobacter_jejuensis |
| OTU04879 | Bacteria | Proteobacteria | Gammaproteobacteria | Pseudomonadales | Moraxellaceae | Acinetobacter | Acinetobacter uncl. |
| OTU04891 | Bacteria | Proteobacteria | Gammaproteobacteria | Pseudomonadales | Moraxellaceae | Alkanindiges | Alkanindiges uncl. |
| OTU04904 | Bacteria | Proteobacteria | Alphaproteobacteria | Acetobacterales | Acetobacteraceae | Commensalibacter | Commensalibacter uncl. |
| OTU04910 | Bacteria | Actinobacteriota | Actinobacteria | Pseudonocardiales | Pseudonocardiaceae | Pseudonocardia | Pseudonocardia_spinosispora |
| OTU04913 | Bacteria | Proteobacteria | Gammaproteobacteria | Enterobacterales | Enterobacterales uncl. | Enterobacterales uncl. | Enterobacterales uncl. |
| OTU04920 | Bacteria | Firmicutes | Bacilli | Lactobacillales | Lactobacillaceae | Bombilactobacillus | Bombilactobacillus uncl. |
| OTU04923 | Bacteria | Proteobacteria | Gammaproteobacteria | Enterobacterales | Enterobacterales uncl. | Enterobacterales uncl. | Enterobacterales uncl. |
| OTU04937 | Bacteria | Proteobacteria | Gammaproteobacteria | Pseudomonadales | Pseudomonadaceae | Pseudomonas | Pseudomonas uncl. |
| OTU04947 | Bacteria | Proteobacteria | Gammaproteobacteria | Enterobacterales | Orbaceae | Gilliamella | Gilliamella uncl. |
| OTU04977 | Bacteria | Proteobacteria | Gammaproteobacteria | Xanthomonadales | Xanthomonadaceae | Lysobacter | Lysobacter_soli |
| OTU04980 | Bacteria | Proteobacteria | Alphaproteobacteria | Acetobacterales | Acetobacteraceae | Commensalibacter | Commensalibacter_sp_AMU001 |
| OTU05006 | Bacteria | Firmicutes | Bacilli | Lactobacillales | Lactobacillaceae | Bombilactobacillus | Bombilactobacillus uncl. |
| OTU05019 | Bacteria | Proteobacteria | Alphaproteobacteria | Rickettsiales | Anaplasmataceae | Wolbachia | Wolbachia uncl. |
| OTU05033 | Bacteria | Proteobacteria | Gammaproteobacteria | Pseudomonadales | Pseudomonadaceae | Pseudomonas | Pseudomonas uncl. |
| OTU05050 | Bacteria | Proteobacteria | Gammaproteobacteria | Enterobacterales | Orbaceae | Gilliamella | Gilliamella uncl. |
| OTU05092 | Bacteria | Proteobacteria | Alphaproteobacteria | Sphingomonadales | Sphingomonadaceae | Sphingomonas | Sphingomonas uncl. |
| OTU05113 | Bacteria | Proteobacteria | Gammaproteobacteria | Pseudomonadales | Pseudomonadaceae | Pseudomonas | Pseudomonas uncl. |
| OTU05121 | Bacteria | Actinobacteriota | Actinobacteria | Pseudonocardiales | Pseudonocardiaceae | Saccharopolyspora | Saccharopolyspora uncl. |
| OTU05166 | Bacteria | Proteobacteria | Gammaproteobacteria | Burkholderiales | Neisseriaceae | Snodgrassella | Snodgrassella_alvi |
| OTU05186 | Bacteria | Proteobacteria | Alphaproteobacteria | Rickettsiales | Anaplasmataceae | Wolbachia | alpha_proteobacterium_endosymbiont_of_Coelostomidia_montana |
| OTU05194 | Bacteria | Proteobacteria | Gammaproteobacteria | Enterobacterales | Enterobacterales uncl. | Enterobacterales uncl. | Enterobacterales uncl. |
| OTU05197 | Bacteria | Proteobacteria | Alphaproteobacteria | Rhizobiales | Rhizobiaceae | Allorhizobium-Neorhizobium-Pararhizobium-Rhizobium | Neorhizobium_sp_NCHU2750 |
| OTU05211 | Bacteria | Chloroflexi | KD4-96 | KD4-96 uncl. | KD4-96 uncl. | KD4-96 uncl. | KD4-96 uncl. |
| OTU05216 | Bacteria | Bacteroidota | Bacteroidia | Flavobacteriales | Weeksellaceae | Apibacter | Apibacter_sp_wkB309 |
| OTU05232 | Bacteria | Proteobacteria | Gammaproteobacteria | Burkholderiales | Oxalobacteraceae | Massilia | Massilia uncl. |
| OTU05245 | Bacteria | Firmicutes | Bacilli | Entomoplasmatales | Spiroplasmataceae | Spiroplasma | Spiroplasma_endosymbiont_of_Curculio_elephas |
| OTU05265 | Bacteria | Proteobacteria | Alphaproteobacteria | Rickettsiales | Anaplasmataceae | Wolbachia | Wolbachia uncl. |
| OTU05268 | Bacteria | Firmicutes | Bacilli | Lactobacillales | Lactobacillaceae | Lactobacillus | Lactobacillus_apis |
| OTU05280 | Bacteria | Actinobacteriota | Actinobacteria | Frankiales | Nakamurellaceae | Nakamurella | Nakamurella uncl. |
| OTU05292 | Bacteria | Actinobacteriota | Actinobacteria | Bifidobacteriales | Bifidobacteriaceae | Bifidobacterium | Bifidobacterium_bombi |
| OTU05295 | Bacteria | Proteobacteria | Alphaproteobacteria | Acetobacterales | Acetobacteraceae | Acetobacteraceae uncl. | Acetobacteraceae uncl. |
| OTU05309 | Bacteria | Proteobacteria | Gammaproteobacteria | Enterobacterales | Enterobacterales uncl. | Enterobacterales uncl. | Enterobacterales uncl. |
| OTU05318 | Bacteria | Proteobacteria | Alphaproteobacteria | Rhizobiales | Beijerinckiaceae | Bosea | Bosea uncl. |
| OTU05319 | Bacteria | Firmicutes | Bacilli | Lactobacillales | Lactobacillaceae | Lactobacillus | Lactobacillus_apis |
| OTU05356 | Bacteria | unclassified | unclassified | unclassified | unclassified | unclassified | unclassified |
| OTU05377 | Bacteria | Proteobacteria | Gammaproteobacteria | Burkholderiales | Neisseriaceae | Neisseriaceae uncl. | Neisseriaceae uncl. |
| OTU05388 | Bacteria | Proteobacteria | Gammaproteobacteria | Pseudomonadales | Moraxellaceae | Acinetobacter | Acinetobacter uncl. |
| OTU05405 | Bacteria | Proteobacteria | Gammaproteobacteria | Burkholderiales | Comamonadaceae | Comamonadaceae uncl. | Comamonadaceae uncl. |
| OTU05462 | Bacteria | Firmicutes | Bacilli | Lactobacillales | Lactobacillaceae | Lactobacillus | Lactobacillus_helsingborgensis |
| OTU05480 | Bacteria | Firmicutes | Bacilli | Lactobacillales | Lactobacillaceae | Lactobacillus | Lactobacillus_helsingborgensis |
| OTU05488 | Bacteria | Firmicutes | Bacilli | Staphylococcales | Staphylococcaceae | Staphylococcus | Staphylococcus uncl. |
| OTU05521 | Bacteria | Proteobacteria | Gammaproteobacteria | Enterobacterales | Orbaceae | Gilliamella | Gilliamella uncl. |
| OTU05531 | Bacteria | Proteobacteria | Alphaproteobacteria | Sphingomonadales | Sphingomonadaceae | Sphingomonas | Sphingomonas uncl. |
| OTU05532 | Bacteria | unclassified | unclassified | unclassified | unclassified | unclassified | unclassified |
| OTU05549 | Bacteria | Firmicutes | Clostridia | Lachnospirales | Lachnospiraceae | Lachnospiraceae uncl. | Lachnospiraceae uncl. |
| OTU05553 | Bacteria | unclassified | unclassified | unclassified | unclassified | unclassified | unclassified |
| OTU05586 | Bacteria | Proteobacteria | Gammaproteobacteria | Burkholderiales | Oxalobacteraceae | Collimonas | Collimonas uncl. |
| OTU05612 | Bacteria | Firmicutes | Bacilli | Lactobacillales | Lactobacillaceae | Bombilactobacillus | Bombilactobacillus uncl. |
| OTU05615 | Bacteria | Proteobacteria | Gammaproteobacteria | Enterobacterales | Yersiniaceae | Serratia | Serratia_symbiotica |
| OTU05650 | Bacteria | Proteobacteria | Alphaproteobacteria | Rhizobiales | Rhizobiaceae | Rhizobiaceae uncl. | Rhizobiaceae uncl. |

Figure captions

**Figure S1:** Bacterial alpha diversity of bee species, expressed as number of observed bacterial Operational Taxonomic Units (OTUs); boxes represent second and third quartiles, whiskers are based on 1.5 ×inter-quartile ranges; bold lines show median; dots represent bee individuals.

**Alt text:** Box plots for all bees on species level.

**Figure S2:** Non-metric multidimensional scaling (NMDS) of bumble bees at different sites, distance measure = Bray-Curtis; dots represent Bsp individuals, label = species name + elevation of sampling (rounded to 50 m), Site 5 and Site 6 show different sampling rounds, Site 9-10 different sites.

**Alt text:** Three ordination plots of bumble bees at different sites and sampling rounds.

**Figure S3:** Linear discriminant analysis Effect Size of the bacterial genera that were most characteristic of the respective bee group; genera with an LDA (linear discriminant analysis) score less than 3 were excluded.

**Alt text:** Bar chart with one bar for every bacterial genus.

Figures


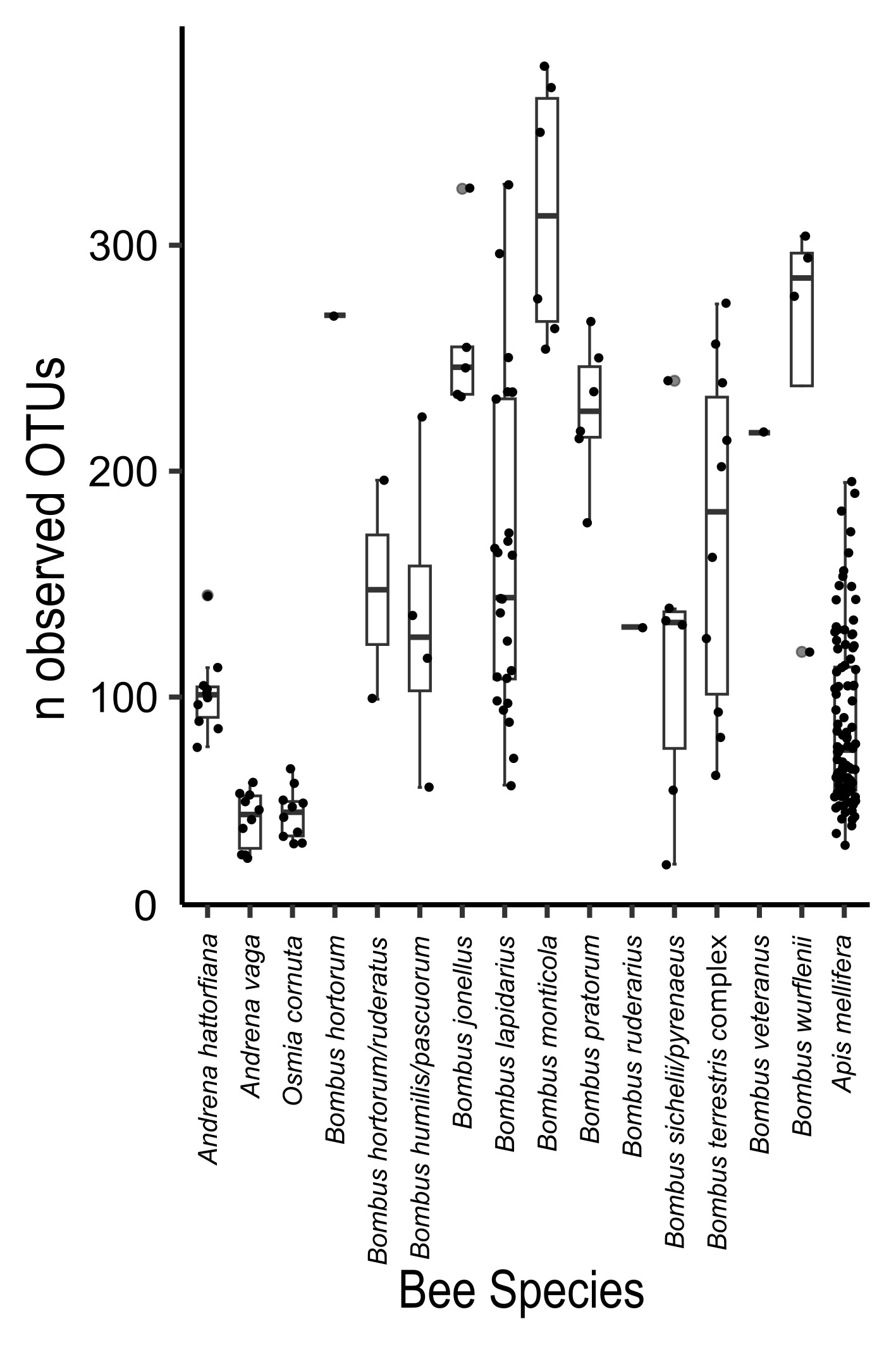


**Figure S1**


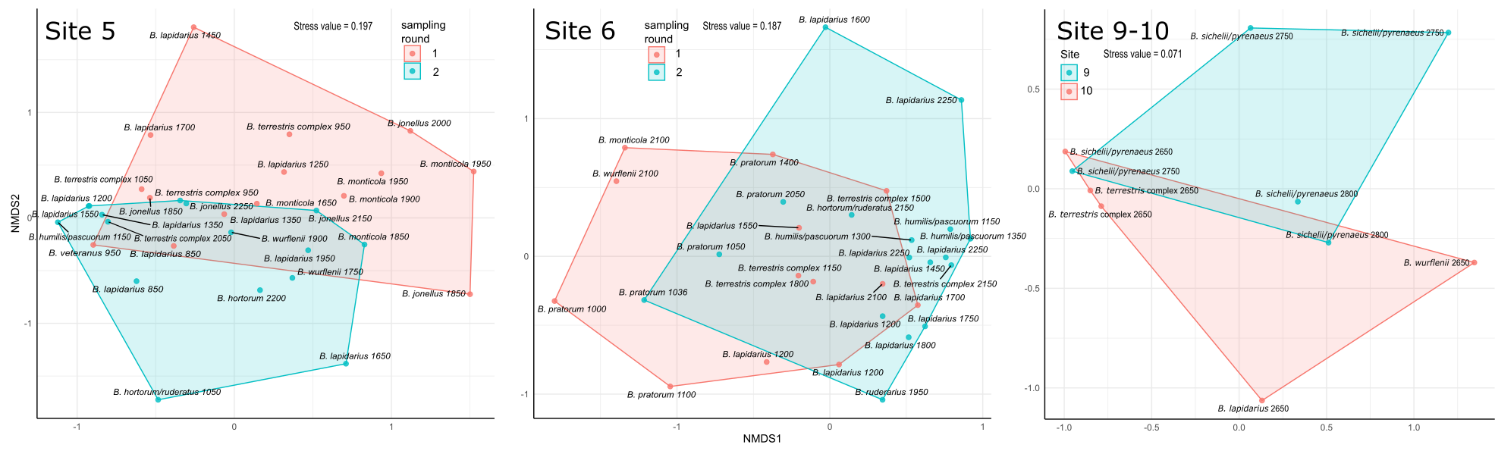
**Figure S2**

**
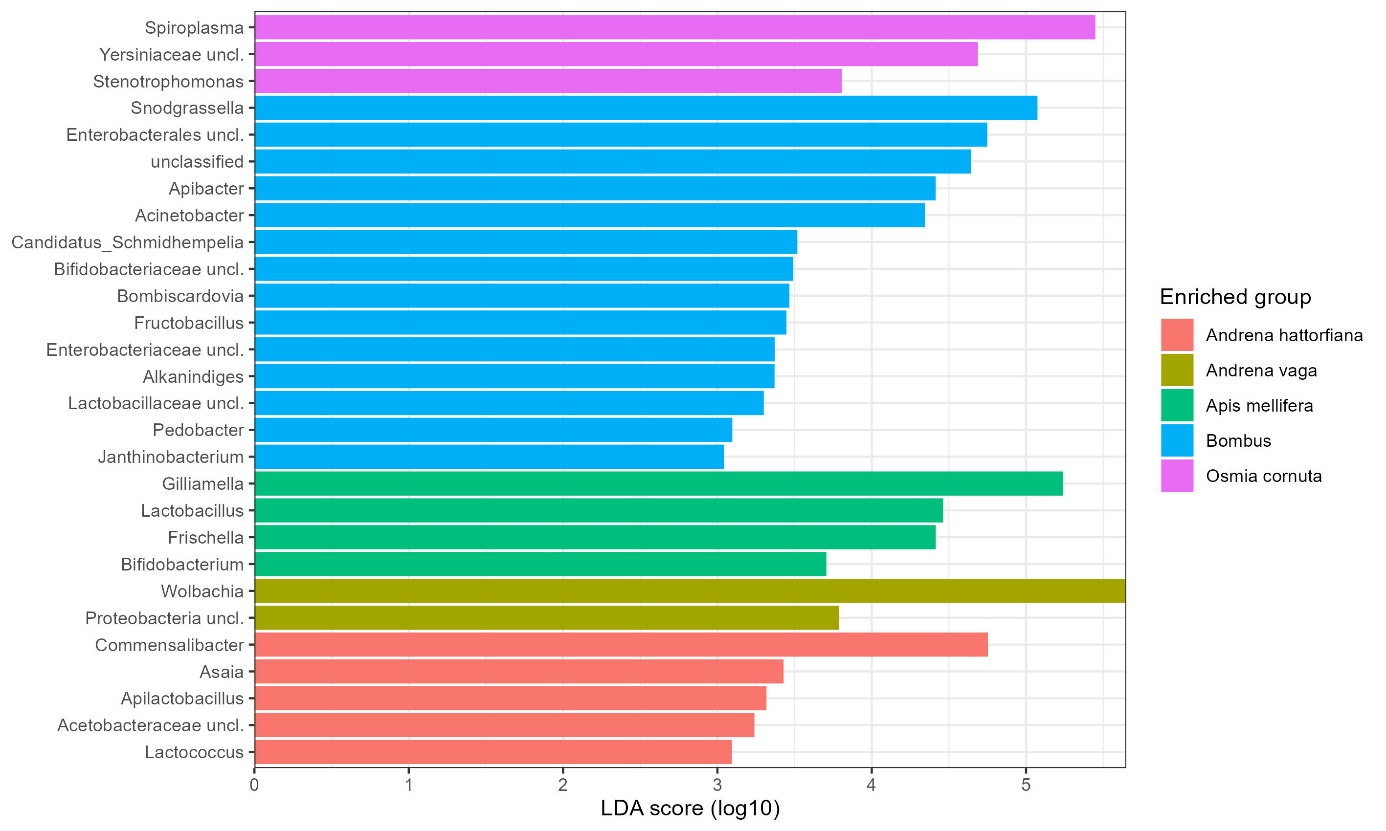
**

**Figure S3**

------------------END-----------------------
